# Annexin A3 potentiates lenvatinib resistance in hepatocellular carcinoma through multiple approaches amplified by a positive feedback loop

**DOI:** 10.1038/s41419-026-08735-9

**Published:** 2026-04-13

**Authors:** Yingqin Zhu, Yue Huang, Mengjia Song, Jieying Yang, Jingjing Zhao, Yan Tang, Dijun Ouyang, Chaopin Yang, Yuanyuan Chen, Qijing Wang, Yongqiang Li, Jia He, Hao Chen, Tong Xiang, Jianchuan Xia, Qiuzhong Pan

**Affiliations:** 1 State Key Laboratory of Oncology in South China, Guangdong Provincial Clinical Research Center for Cancer, Sun Yat-sen University Cancer Center, Guangzhou, China; 2 Department of Pancreato-Biliary Surgery, First Affiliated Hospital, Sun Yat-Sen University, Guangzhou, China; 3 Department of Medical Oncology, The Second Affiliated Hospital of Guangzhou Medical University, Guangzhou Medical University, Guangzhou, China; 4 Department of Pediatric Oncology, Sun Yat-sen University Cancer Center, Guangzhou, China; 5 Department of Biotherapy, Sun Yat-sen University Cancer Center, Guangzhou, China

**Keywords:** Cancer therapy, Target identification

## Abstract

Hepatocellular carcinoma (HCC) is one of the most lethal malignancies with limited treatment options. Lenvatinib is a first-line drug for advanced HCC. However, its effect on patient survival is limited and patients ultimately develop disease progression due to drug resistance. In this study, we established a lenvatinib-resistant orthotopic xenograft model and found a significant upregulation of ANXA3. Further researches revealed that ANXA3 facilitated lenvatinib resistance in vitro and in vivo. Mechanistically, ANXA3 activated the PI3K pathway and enhanced the transcription of PDGF-AA, thus promoting tumor angiogenesis. Besides, ANXA3 also promoted EMT and autophagy through the PI3K pathway. These three effects were comprehensively responsible for the roles of ANXA3 in facilitating lenvatinib resistance. What’s more, the secreted PDGF-AA could in turn activate the PI3K pathway, thus forming a positive feedback loop, which could amplify the effects driven by ANXA3. Alpelisib is a PI3K inhibitor approved for breast cancer treatment by FDA. We demonstrated that Alpelisib may synergistically improve the antitumor effects of lenvatinib without increasing side effects. This study reports ANXA3 as a biomarker o predict poor prognosis and lenvatinib resistance in HCC. The combined use of Alpelisib and lenvatinib may serve as a potential therapeutic strategy for lenvatinib-resistant HCC.

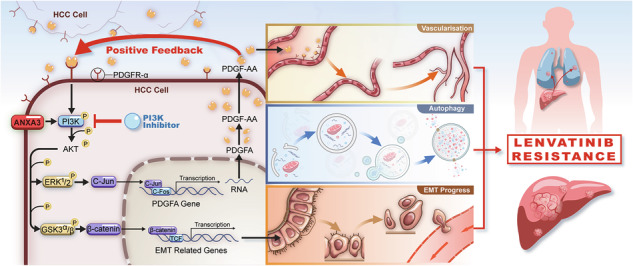

## Introduction

Hepatocellular carcinoma (HCC) is among the most aggressive malignancies worldwide and is associated with a dismal prognosis [1–3]. Owing to the insidious onset of the disease, the majority of patients are diagnosed at advanced stages, at which point curative treatments are no longer feasible and systemic therapies-such as sorafenib [4], lenvatinib [5] or immune oncology combination therapy [6, 7]- remain the primary therapeutic options. Since 2018, lenvatinib has been approved as a first-line treatment for advanced HCC in USA, EU, and China [8]. Mechanistically, the antitumor activity of lenvatinib is attributed to both potent inhibition of tumor angiogenesis and direct suppression of tumor cell proliferation [9]. However, despite initial therapeutic responses, lenvatinib rarely induces complete tumor regression, and the majority of patients eventually experience disease progression [10, 11]. Since the precise mechanisms underlying resistance to lenvatinib are barely understood, there is an urgent need to identify new reliable biomarkers that can predict the outcome of lenvatinib treatment and therapeutic strategies that can improve the efficacy of lenvatinib.

Our group has previously demonstrated the critical role of Annexin A3 (ANXA3) in promoting tumorigenesis, chemoresistance [12], immune microenvironment remodeling [13] and tumor stemness [14] in HCC. Beyond our own findings, aberrant ANXA3 expression has been widely reported to correlate with aggressive tumor phenotypes, including enhanced stemness [15] and metastatic potential [16–23]. Notably, Tong et al. revealed that the use of anti-ANXA3 mAb sensitized HCC to sorafenib and regorafenib [24]. In light of these interesting findings, we established a lenvatinib-resistant orthotopic xenograft model to further investigate the role of ANXA3 in therapeutic resistance. We found that the expression of ANXA3 was significantly upregulated in lenvatinib-resistant tumors, which was further confirmed with patients’ tumors. Intriguingly, we observed that CD34 MVD (microvessel density) raised in tandem with ANXA3 expression in lenvatinib-resistant HCC. Consistent with our observations, previous studies have reported that ANXA3 silencing suppresses lymphatic vascularization in pancreatic cancer cells [25] and reduces intratumoral vessel formation in triple-negative breast cancer xenografts [26], implicating ANXA3 as a putative pro-angiogenic factor. In our study, we innovatively explored the effect of ANXA3 in promoting lenvatinib resistance from multiple perspectives of angiogenesis, EMT-driven metastasis, and autophagy comprehensively. What’s more, the positive feedback loop mediated by phosphoinositide 3-kinase (PI3K) amplificated the effect driven by ANXA3.

The PI3K signaling pathway is one of the most frequently aberrantly-activated oncogenic pathways in cancer, and its dysregulation is widely recognized as a hallmark of cancer. Alpelisib is an orally administered PI3K inhibitor that was approved by the FDA to treat breast cancers in 2019 [27]. Previous studies have reported that Alpelisib is effective for treating HCC [28] and breast cancer [29]. Given that ANXA3 promotes lenvatinib resistance via PI3K signaling pathway, we further evaluated the therapeutic efficacy of combining Alpelisib with lenvatinib. Our study presented the first evidence effects that combined use of Alpelisib and lenvatinib has a synergistic efficacy in HCC, providing a potential adjuvant therapeutic strategy for HCC patients.

## Materials and methods

### Patients and specimens

All human samples were obtained from Sun Yat­sen University Cancer Center (SYSUCC). A total of 55 HCC samples were retrieved from HCC patients who underwent curative resection followed by lenvatinib treatment between November 2018 and December 2019. For testing the expression of ANXA3 in HCC, a total of 149 paraffin-embedded HCC samples were obtained from HCC patients who underwent hepatectomy between February 2002 and December 2004. For qPCR assays, 42 specimens of HCC tissues paired with non-tumorous liver tissues were collected from patients with primary HCC between December 2005 and May 2008. The diagnosis was histologically confirmed by pathological examination, and none of the patients had received any antitumor therapy before surgery. Written informed consent was obtained from all patients before sample collection, and ethics approval for the use of specimens was provided by the Internal Review and the Ethics Boards of the Sun Yat-Sen University Cancer Center (Approval No. G2022-172-01). All experiments involving humans were carried out in accordance with the Code of Ethics of the World Medical Association (Declaration of Helsinki).

### Cell lines and animals

HepG2, Hep3B, SK-Hep-1, 293T cell lines and human umbilical vein endothelial cells (HUVECs) were purchased from the Cell Bank of the Chinese Academy of Sciences (Shanghai, China). All mice were obtained from GemPharmatech Co., Ltd. (Nanjing, China) and maintained under specific pathogen-free conditions. The animal experiments were approved by the Institutional Animal Care and Use Committee of the Sun Yat-Sen University Cancer Center (Approval No. L102022022004A). All animals received humane care according to the criteria outlined in the “Guide for the Care and Use of Laboratory Animals”.

Additional information on methods is available in the Supplementary materials and methods.

## Results

### High expression of ANXA3 is closely correlated with lenvatinib resistance and poor prognosis in HCC

**Fig. 1:**
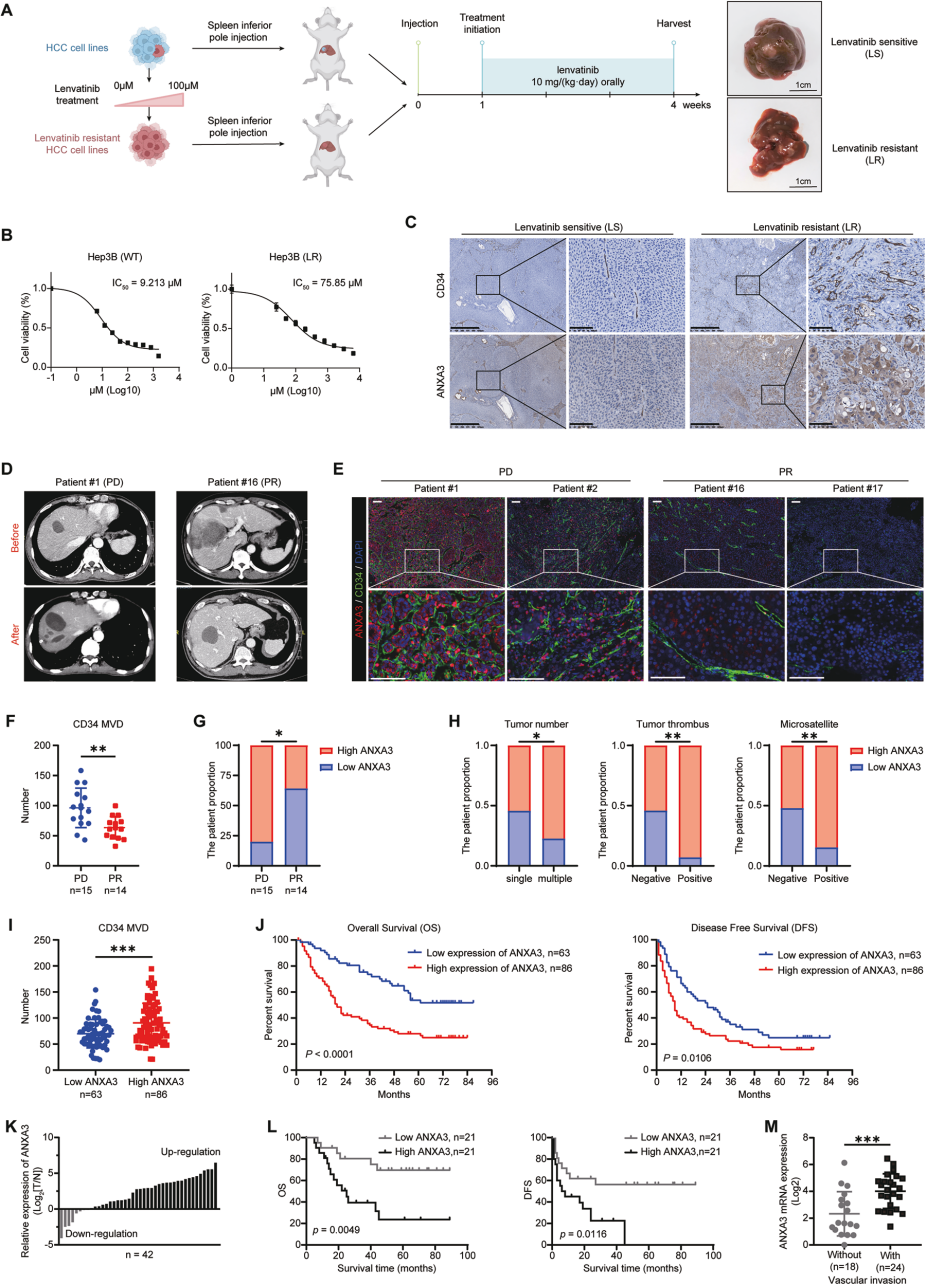
High expression of ANXA3 is closely correlated with lenvatinib resistance and poor prognosis in HCC. Diagram showing the construction of lenvatinib-resistant orthotopic xenograft models of HCC. Scale bars, 1 cm. **A** The IC_50_ value of Hep3B wild-type (WT) and Hep3B lenvatinib resistant (LR). **B** Typical IHC images of CD34 and ANXA3 expression status in orthotopic xenograft models of HCC (*n* = 5). Scale bars, 625 μm or 100 μm. **C** Typical CT photos of patients who developed partial response (PR) or progressive disease (PD). **D** mIHC images showing the co-expression pattern of ANXA3 (red) and CD34 (green) in patients who developed PR (*n* = 14) or PD (*n* = 15). DAPI (bule). Scale bars, 100 μm. **E** Comparison of CD34 MVD between lenvatinib-resistant and -sensitive HCC tissues. **F** The percentages of ANXA3 expression status in lenvatinib-resistant and -sensitive HCC tissues. **G** Comparison of the percentages of ANXA3 expression status according to tumor number, tumor thrombus, microsatellite, *n* = 149. **H** Comparison of CD34 MVD according to ANXA3 expression status, *n* = 149. **I** Kaplan–Meier analysis of Overall Survival (OS) and Disease Free Survival (DFS) according to ANXA3 expression status in 149 HCC patients. **J** qPCR analysis of ANXA3 mRNA expression levels in the validation cohort (*n* = 42). **K** Kaplan–Meier analysis of OS and DFS according to relative ANXA3 mRNA levels (*n* = 42). **L** Comparison of ANXA3 mRNA expression in patients with or without vascular invasion (*n* = 42). **M** The results represent three independent experiments. Error bars represent the mean ± SD. *P < 0.05; **P < 0.01; ***P < 0.001 according to Student’s *t* test.

We first established lenvatinib-resistant HCC cell lines and orthotopic xenograft models (Fig. 1A, B) to further investigate the role of ANXA3. ANXA3 expression was markedly upregulated in lenvatinib-resistant xenografts. Notably, tumors resistant to lenvatinib exhibited significantly increased vascular density (Fig. 1C). To evaluate the clinical relevance of ANXA3, we analyzed tumor samples from 55 HCC patients who received lenvatinib treatment. According to Response Evaluation Criteria in Solid Tumors (RECIST 1.1), 15 patients achieved partial response (PR), whereas 14 developed progressive disease (PD) (Fig. 1D). Multiplex immunohistochemistry (mIHC) staining revealed that both CD34-defined microvessel density (MVD) and ANXA3 expression were significantly elevated in lenvatinib-resistant HCC tumors (Fig. 1E, F). Moreover, patients with low ANXA3 expression were more likely to achieve PR following lenvatinib treatment than those with high ANXA3 expression (Fig. 1G).

We next examined ANXA3 expression in a larger cohort comprising 149 HCC tissue specimens (Fig. S1A). ANXA3 expression was strongly associated with tumor number, tumor thrombus, microsatellite formation, tumor size, and TNM stage (Figs. 1H and S1B and Table S3). In addition, a significant positive correlation was observed between ANXA3 expression and CD34 MVD (Fig. 1I), suggesting a potential role of ANXA3 in promoting tumor angiogenesis in HCC. Kaplan–Meier survival analysis demonstrated that patients with high ANXA3 expression had significantly shorter overall survival (OS) and disease-free survival (DFS) (Fig. 1J). Multivariate Cox regression analysis further identified ANXA3 expression as an independent prognostic factor for OS (Tables S4 and 5).

These findings were validated in an independent cohort of 42 paired HCC and adjacent normal liver tissues at the RNA level (Fig. 1K, L). Notably, tumors with vascular invasion exhibited significantly higher ANXA3 expression than those without vascular invasion (Fig. 1M). Taken together, these results indicate that ANXA3 is a clinically relevant molecule associated with angiogenesis, tumor progression, and resistance to lenvatinib in HCC.

### ANXA3 promotes lenvatinib resistance in HCC both in vitro and in vivo

**Fig. 2:**
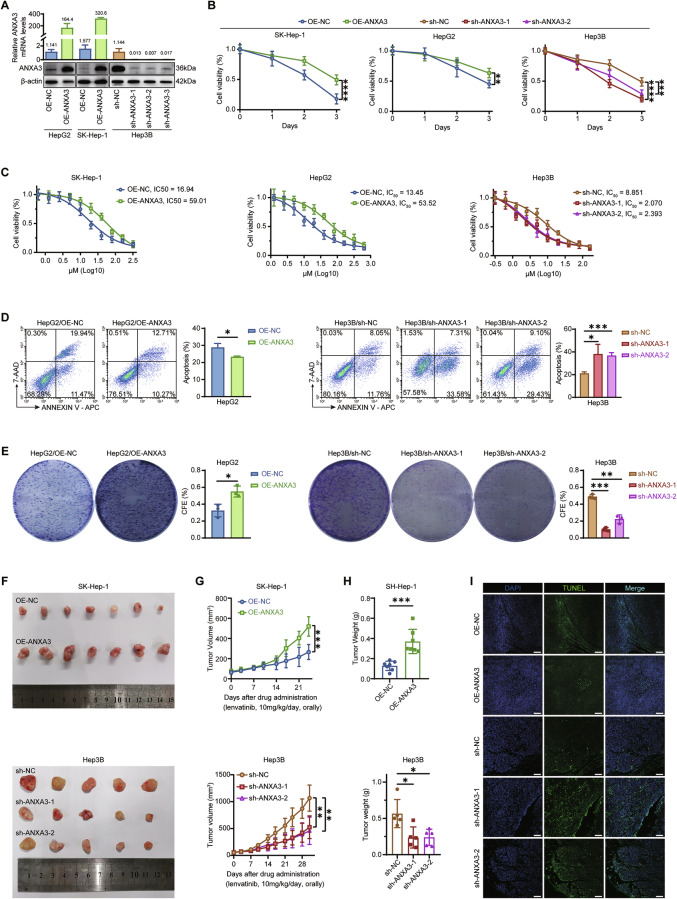
ANXA3 promotes lenvatinib resistance in HCC both in vitro and in vivo. **A** qPCR (top) and Western blotting (bottom) results validated the regulation of ANXA3 in HepG2, SK-Hep-1, and Hep3B. **B** The indicated cells were treated with lenvatinib for different durations and cell viability was measured by the CCK-8 assays. **C** The IC_50_ value of HCC cells with different levels of ANXA3. **D** The cell apoptotic rates of the indicated HCC cells which were treated with lenvatinib for 72 h. **E** Colony formation of the indicated HCC cells following lenvatinib treatment. **F** Mice harboring xenografts derived from the indicated cells were treated with lenvatinib (10 mg/(kg·d), orally). The pictures showing the dissected tumors from each group (*n* = 5). **G** Graphs showing the growth curve of xenografts from each group (*n* = 5). **H** Graphs showing the tumor weight of xenografts from each group (*n* = 5). **I** Representative images of TUNEL staining of xenografts from each group (*n* = 5). Scale bars, 100 μm. The results represent three independent experiments. Error bars represent the mean ± SD. *P < 0.05; **P < 0.01; ***P < 0.001 according to Student’s *t* test.

Next, we investigated the role of ANXA3 in regulating lenvatinib resistance in HCC. ANXA3 expression was first examined at both the mRNA and protein levels by qRT–PCR (Fig. S2A) and Western blotting (Fig. S2B), respectively, in a panel of commonly used HCC cell lines, including HepG2, Hep3B, SK-Hep-1, Huh7, and PLC/PRF/5. ANXA3 expression was highest in Hep3B cells, whereas relatively low expression levels were observed in HepG2 and SK-Hep-1 cells. Based on this expression pattern, ANXA3 was silenced in Hep3B cells and ectopically overexpressed in HepG2 and SK-Hep-1 cells for subsequent functional analyses (Fig. 2A). Cell Counting Kit-8 (CCK-8) assays demonstrated that ANXA3 significantly enhanced HCC cell viability following lenvatinib treatment (Fig. 2B) and markedly increased the half-maximal inhibitory concentration (IC_50_) of lenvatinib (Fig. 2C). Consistently, ANXA3-proficient cells exhibited a significantly lower apoptotic rate (Figs. 2D and S3A) and increased colony- and sphere-forming capacities following lenvatinib treatment compared with ANXA3-deficient cells (Figs. 2E and S3B, C).

To further assess the impact of ANXA3 on lenvatinib sensitivity in vivo, subcutaneous xenograft models were established and treated with lenvatinib (10 mg/(kg·d), orally). ANXA3 expression markedly attenuated the antitumor efficacy of lenvatinib in vivo, as evidenced by increased tumor growth in ANXA3-expressing xenografts (Fig. 2F–H). Accordingly, a significantly higher proportion of apoptotic cells was observed in tumors with low ANXA3 expression (Fig. 2I). Collectively, these results demonstrate that ANXA3 reduces the sensitivity of HCC cells to lenvatinib both in vitro and in vivo.

### ANXA3 reduces lenvatinib sensitivity by modulating autophagy and EMT

**Fig. 3:**
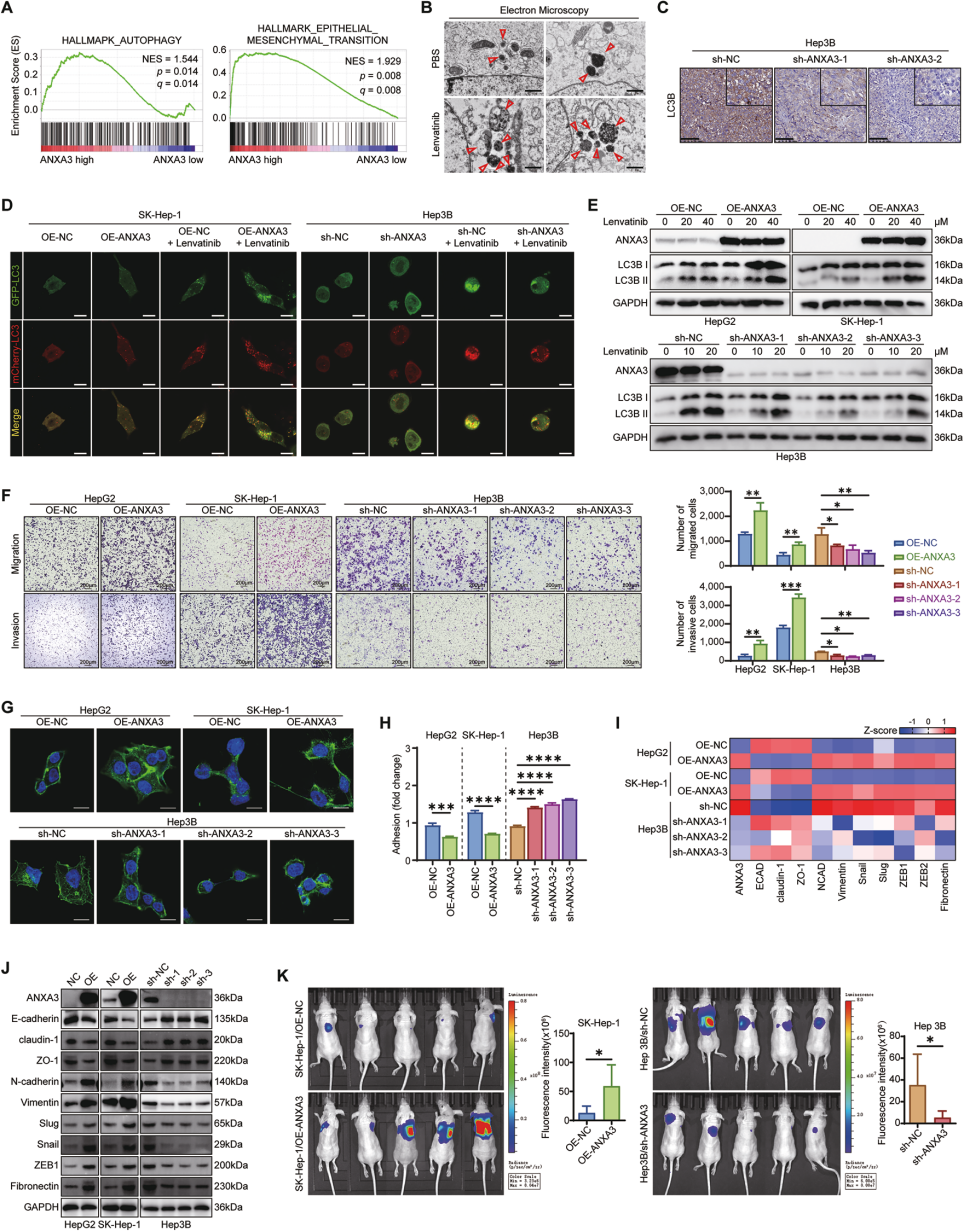
ANXA3 reduces lenvatinib sensitivity by modulating autophagy and EMT. **A** GSEA showing that the expression level of ANXA3 is positively correlated with autophagy and EMT. **B** Representative electron micrographs of autophagic vesicles in Hep3B cells treated with or without lenvatinib. Scale bar, 1 μm. **C** Representative images of LC3B staining in xenografts from mice treated with Lenvatinib (*n* = 5). Scale bar, 100 μm. **D** The effect of ANXA3 on autophagy flux were analyzed by mCherry-GFP-LC3 reporter assay. Scale bars, 10 μm. **E** Western blot analysis showing the expression of LC3B in the indicated cells treated with increasing concentration of lenvatinib. **F** Transwell assays showing the migratory and invasive capacities of the indicated cells following lenvatinib treatment. Scale bars, 200 μm. **G** IF assays for phalloidin (green) staining in the indicated HCC cells. DAPI (blue). Scale bars, 10 μm. **H** Cell adhesion assays performed in the indicated HCC cells. **I** qPCR array showing the expression of EMT-related genes. **I** Western blotting showing the expression of EMT-related genes. **J** Representative bioluminescent images and photo flux of lung metastatic tumors in the tested mice (*n* = 5). **K** The results represent three independent experiments. Error bars represent the mean ± SD. *P < 0.05; **P < 0.01; ***P < 0.001 according to Student’s *t* test.

Gene set enrichment analysis (GSEA) of The Cancer Genome Atlas (TCGA) dataset revealed that ANXA3 expression was positively correlated with autophagy and epithelial–mesenchymal transition (EMT) in HCC (Figs. 3A and S4A). We first investigated the effect of ANXA3 on autophagy. Transmission electron microscopy (TEM) confirmed the abundant presence of vesicular structures morphologically resembling autophagosomes and autolysosomes in Hep3B cells treated with lenvatinib (Fig. 3B). IHC staining of mouse xenografts further demonstrated a significant decrease in LC3B in tumors with ANXA3 knockdown (Fig. 3C). Autophagic flux analysis indicated that the number of LC3 puncta co-expressing mCherry and GFP increased upon ANXA3 overexpression and decreased upon ANXA3 depletion (Fig. 3D). Western blotting further revealed that lenvatinib treatment induced a markedly stronger accumulation of LC3B-II in ANXA3-proficient cells (Fig. 3E). To assess the functional role of autophagy in ANXA3-mediated lenvatinib resistance, we treated ANXA3-overexpressing HCC cells with the autophagy inhibitor hydroxychloroquine (HCQ). Apoptosis assays (Fig. S4B) and IC_50_ analysis (Fig. S4C) showed that inhibition of autophagy significantly increased the sensitivity of liver cancer cells to lenvatinib. These results indicate that ANXA3-induced autophagy functions as a cytoprotective mechanism rather than a cell death pathway in the context of lenvatinib treatment.

Next, we investigated the role of ANXA3 in EMT of HCC cells. ANXA3 overexpression enhanced the migratory and invasive capacities of HCC cells under lenvatinib treatment, whereas ANXA3 knockdown markedly impaired these abilities (Fig. 3F). Moreover, ANXA3 markedly increased the formation of spike-like protrusions and mesenchymal-like morphology at the cell edges (Fig. 3G), and reduced cell adhesion (Fig. 3H). Consistently, a panel of EMT-related genes, including N-cadherin (NCAD), vimentin, MMP1, and fibronectin, was significantly upregulated in cells with high ANXA3 expression, while E-cadherin (ECAD) expression was downregulated (Fig. 3I, J). To further assess the in vivo relevance, a lung metastasis model demonstrated that high ANXA3 expression significantly increased the number of metastatic nodules in the lungs (Figs. 3K and S4D). Collectively, these results indicate that ANXA3 promotes both autophagy and EMT in HCC, which likely contribute to lenvatinib resistance.

### ANXA3 facilitates angiogenesis of HCC by upregulating the expression of PDGF-AA

**Fig. 4:**
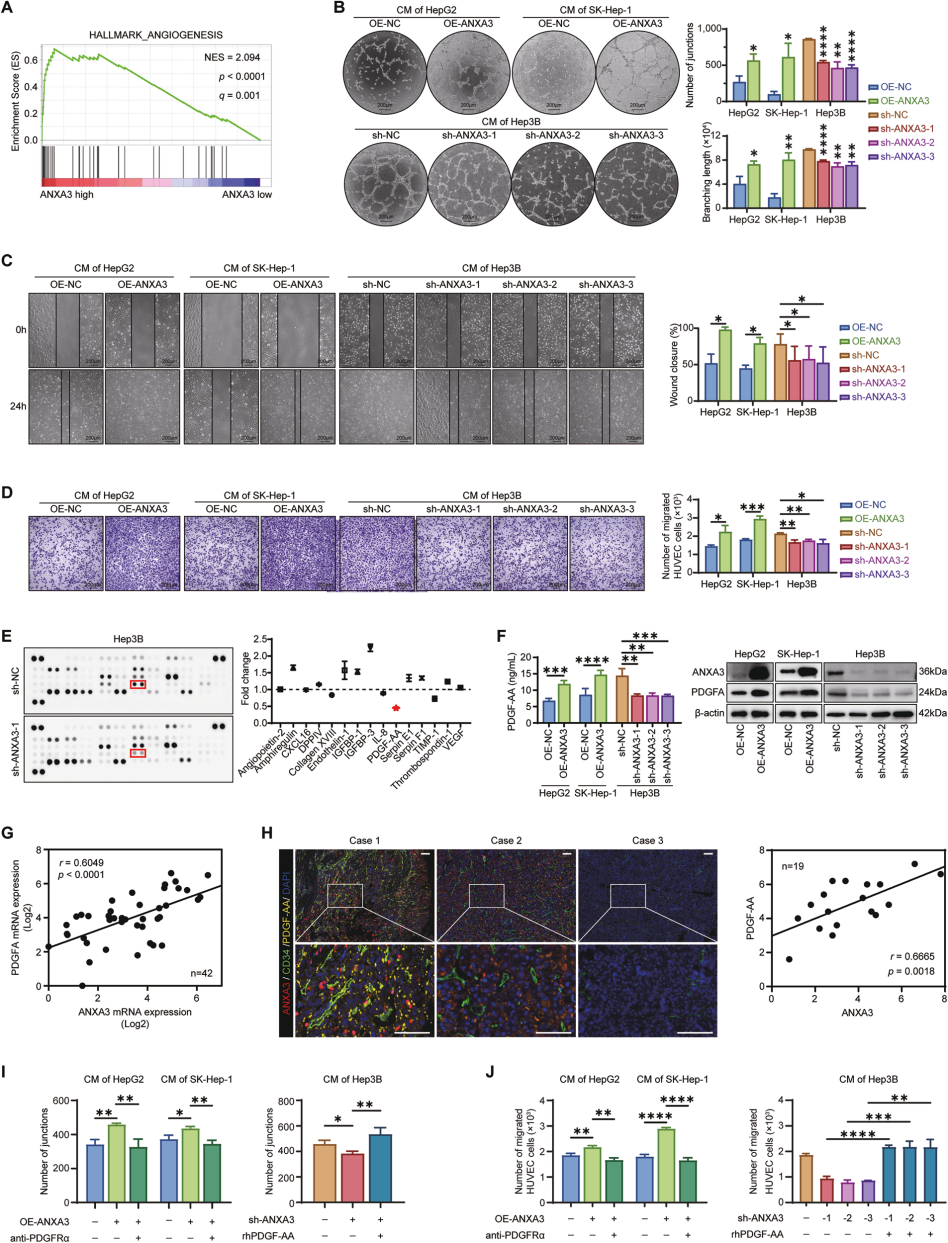
ANXA3 facilitates angiogenesis of HCC by upregulating the expression of PDGF-AA. **A** GSEA showing that the expression level of ANXA3 is positively correlated with angiogenesis. **B**–**D** Tube formation assays (**B**), wound healing assays (**C**) and transwell assays (**D**) were performed on HUVECs using the conditioned media (CM) derived from the indicated cells. Scale bars, 200 μm. **C** Proteome angiogenesis array showing the expression changes of angiogenesis-related soluble proteins in the ANXA3-knockout Hep3B cells. **E** The expression of PDGF-AA in the indicated cells was detected by ELISA (left), and Western blotting (right). **F** qPCR analysis showing the correlation between *ANXA3* and *PDGFA* mRNA levels in HCC tissues (*n* = 42). **G** Typical mIHC images showing the expression of ANXA3 (red), CD34 (green), PDGF-AA (yellow) and DAPI (blue) in HCC tissues (*n* = 19). Scale bars, 100 μm (left). Statistical analysis showing the correlation between PDGF-AA and ANXA3 expression levels (right). **H** The CM of HepG2 and SK-Hep-1 cells with ANXA3-overexpressing were applied to perform tube formation assays on HUVECs with or without anti-PDGFRα (left). The CM of Hep3B cells with ANXA3-knockout were applied to perform tube formation assays on HUVECs in the absence or presence of rhPDGF-AA (right). **I** The CM of HepG2 and SK-Hep-1 cells with ANXA3-overexpressing were applied to perform transwell assays on HUVECs with or without anti-PDGFRα (left). **J** The CM of Hep3B cells with ANXA3-knockout were applied to perform transwell assays on HUVECs in the absence or presence of rhPDGF-AA (right). The results represent three independent experiments. Error bars represent the mean ± SD. *P < 0.05; **P < 0.01; ***P < 0.001 according to Student’s *t* test.

The antitumor effect of lenvatinib relies not only on the direct killing of tumor cells but also on its ability to inhibit tumor angiogenesis. Further GSEA analysis revealed that ANXA3 expression was positively correlated with angiogenesis (Figs. 4A and S5A). To evaluate the role of ANXA3 in angiogenesis, we performed tube formation, wound healing, and transwell assays in HUVECs using conditioned media (CM) derived from the indicated cells. HUVECs incubated with CM from ANXA3-overexpressing cells exhibited enhanced angiogenic and migratory capacities, whereas CM from ANXA3-knockout cells significantly attenuated these capacities (Fig. 4B–D). To investigate the underlying mechanism, the supernatant of ANXA3-deficient HCC cells was analyzed using a Proteome Profiler Human Angiogenesis Array Kit. Platelet-derived growth factor AA (PDGF-AA) was identified as the most strongly downregulated protein (Fig. 4E). qPCR (Fig. S5B), enzyme-linked immunosorbent assay (ELISA), and Western blotting (Fig. 4F) confirmed that PDGF-AA expression was markedly upregulated by ANXA3 overexpression and downregulated upon ANXA3 depletion. Furthermore, qPCR and mIHC analysis revealed a positive correlation between ANXA3 and PDGF-AA expression in human HCC samples (Fig. 4G, H). Finally, functional assays demonstrated that blocking PDGFRα abrogated the promoting effects of ANXA3 on tube formation and migration of HUVECs, whereas recombinant human PDGF-AA (rhPDGF-AA) rescued the tube formation and migration capacities inhibited by ANXA3 depletion (Figs. 4I, J and S5C, D). Collectively, these findings indicate that PDGF-AA mediates ANXA3-induced angiogenesis in HCC.

### The activation of PI3K signaling pathway is responsible for ANXA3-mediated lenvatinib resistance

**Fig. 5:**
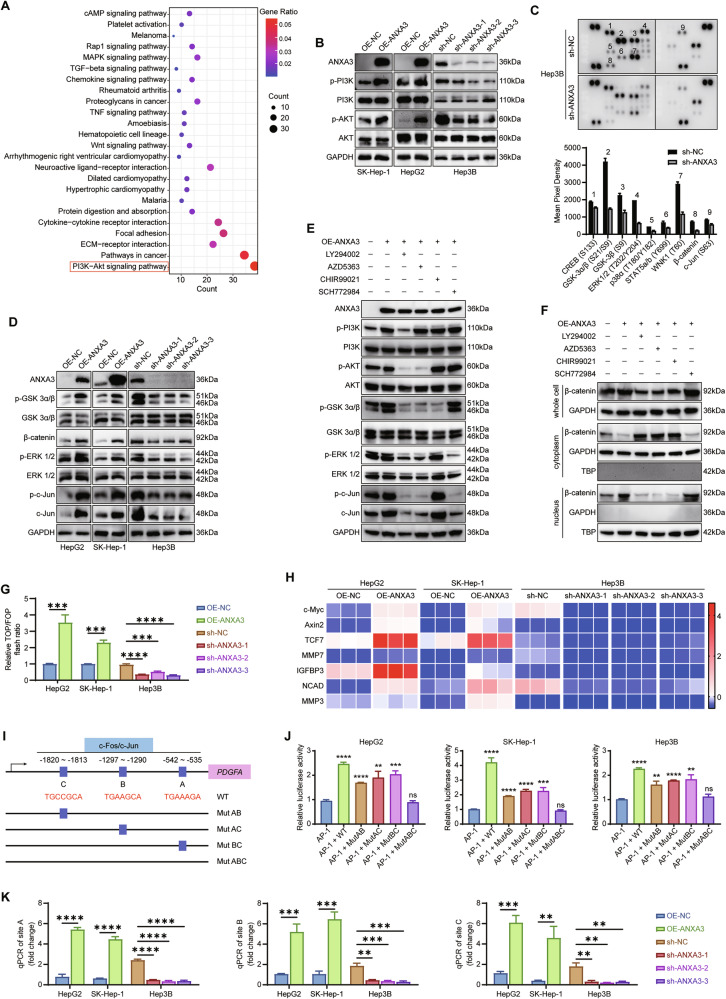
The activation of PI3K signaling pathway is responsible for ANXA3-mediated lenvatinib resistance. **A** KEGG pathway enrichment analysis shows the pathways activated by ANXA3 high expression. **B** Western blotting analysis of the expression of p-PI3K, total PI3K, p-AKT and total AKT. **C** Proteome Phospho-Kinase Array shows the relative levels of human protein kinase phosphorylation in the indicated cells. **D** Western blotting showing the expression of p-GSK 3α/β, β-catenin, p-ERK 1/2, and p-c-Jun in the indicated cells. **E** HepG2/OE-ANXA3 cells were treated with DMSO, LY294002 (PI3K inhibitor, 10 μM, 24 h), AZD5363 (Akt inhibitor, 0.5 μM, 24 h), CHIR99021 (GSK3α/β inhibitor, 5 μM, 24 h), or SCH772984 (ERK1/2 inhibitor, 0.5 μM, 24 h). Western blotting shows the expression of the indicated proteins in the whole cell. **F** Western blotting of the expression of β-catenin in the whole cell, cytoplasm and nucleus. **G** Luciferase assays showing the transcriptional activity of TCF/LEF in the indicated cells. **H** qPCR assays showing the mRNA expression of TCF/LEF targeted genes involved in EMT. **I** Diagram illustrating the promoter region of *PDGFA* with the putative AP-1 (c-Fos/c-Jun) binding motifs, as well as luciferase reporters with the wild-type (WT) motifs or mutant motifs (Mut AB, Mut AC, Mut BC and Mut ABC). **J** Relative luciferase activity of the indicated promoter vectors in HCC cells co-transfected with c-Fos and c-Jun plasmids. **K** ChIP assays were performed using c-Jun antibodies, followed by qPCR analysis in the indicated cells. The results represent three independent experiments. Error bars represent the mean ± SD. *P < 0.05; **P < 0.01; ***P < 0.001 according to Student’s *t* test.

Kyoto Encyclopedia of Genes and Genomes (KEGG) pathway enrichment analysis of the TCGA dataset revealed a significant association between ANXA3 and the PI3K–AKT signaling pathway (Fig. 5A). Consistently, RNA-seq analysis of Hep3B cells with ANXA3 knockdown indicated that ANXA3 was linked to autophagy, angiogenesis, and the PI3K signaling pathway (Fig. S6A, B). Western blotting confirmed that ANXA3 markedly enhanced the phosphorylation of PI3K and AKT (Fig. 5B). In parallel, analysis using a Proteome Profiler Human Phospho-Kinase Array Kit demonstrated that ANXA3 knockdown in Hep3B cells significantly reduced the phosphorylation of GSK3α/β, ERK1/2, and c-Jun, as well as the expression of β-catenin (Fig. 5C), which was subsequently confirmed by Western blotting (Fig. 5D). To dissect the signaling cascades downstream of ANXA3, specific inhibitors of PI3K (LY294002), AKT (AZD5363), GSK3α/β (CHIR99021), and ERK1/2 (SCH772984) were applied. The results indicated that ANXA3 simultaneously activated the PI3K–AKT-GSK3-β-catenin and the PI3K–AKT-ERK1/2-c-Jun signaling cascades (Figs. 5E, F and S7A, B). Notably, none of these inhibitors affected ANXA3 expression.

β-Catenin translocation from the cytosol to the nucleus enables interaction with the T-cell factor/lymphoid enhancer factor (TCF/LEF) transcription factors, thereby activating the transcription of genes involved in tumor metastasis [30, 31]. We next investigated whether ANXA3 modulates TCF/LEF transcriptional activity. Dual-luciferase assays revealed that ANXA3 significantly increased relative TOP/FOP luciferase activities (Fig. 5G). Consistently, qPCR analysis of TCF/LEF target genes involved in EMT demonstrated that ANXA3 positively regulated the mRNA expression of MYC, AXIN2, TCF7, MMP7, IGFBP3, and NCAD (Fig. 5H).

To investigate the transcriptional regulatory mechanisms of *PDGFA*, the putative promoter region of *PDGFA* was analyzed using the UCSC, JAPAR, and PROMO bioinformatic tools (Table S6). Activator protein-1 (AP-1), composed of c-Fos and c-Jun, was predicted as the top-scoring transcription factor for *PDGFA*, and three AP-1–binding motifs were identified at positions −535 to −542, −1290 to −1297, and −1813 to −1820 (Fig. 5I). Dual-luciferase assays demonstrated that AP-1 could promote *PDGFA* transcription even when sites A and B, sites A and C, or sites B and C were mutated; however, mutation of all three binding sites abolished AP-1–mediated transcriptional activity (Fig. 5J). Chromatin immunoprecipitation (ChIP) assays confirmed that AP-1 directly bound to all three c-Fos/c-Jun motifs in HCC cells, and that ANXA3 enhanced this transcriptional activity (Fig. 5K).

Collectively, these results indicate that ANXA3 activates the PI3K–AKT signaling pathway, which further promotes EMT in HCC cells through the GSK3-β-catenin-TCF/LEF signaling axis and accelerates *PDGFA* transcription via the ERK1/2-c-Jun signaling pathway (schematic diagram shown in Fig. S8).

### Blocking the PI3K/PDGF-AA positive feedback loop abolished ANXA3-mediated effects

**Fig. 6:**
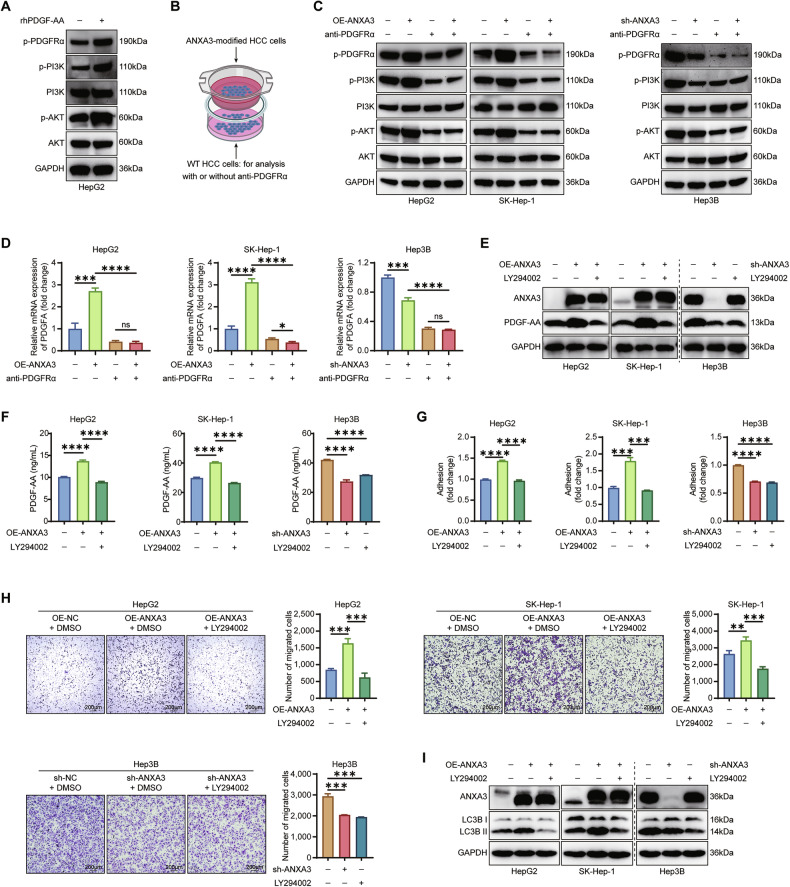
Blocking the PI3K/PDGF-AA positive feedback loop abolished ANXA3-mediated effects. **A** Western blotting showing the expression of the indicated proteins in HepG2 cells treated with or without rhPDGF-AA. **B** Diagram depicting the co-culture of wild-type HCC cells and the ANXA3-modified HCC cells. **C** Western blotting showing the expression of the indicated proteins in wild-type HCC cells co-cultured with the ANXA3-modified HCC cells in the presence or absence of anti-PDGFRα. **D** qPCR assays showing the mRNA expression of *PDGFA* in wild-type HCC cells co-cultured with the indicated cells in the absence or presence of anti-PDGFRα. **E** Western blotting showing the expression of PDGF-AA in the indicated cells treated with or without LY294002. **F** ELISA showing the levels of PDGF-AA in the CM derived from the indicated cells treated with or without LY294002. **G** Cell adhesion assays of the indicated cells in the presence or absence of LY294002. **H** Cell migration assays of the indicated cells in the presence or absence of LY294002, Scale bars, 200 μm. **I** Western blot analysis for the expression of LC3B in the indicated cells treated with lenvatinib in the presence or absence of LY294002. The results represent three independent experiments. Error bars represent the mean ± SD. *P < 0.05; **P < 0.01; ***P < 0.001 according to Student’s *t* test.

It is well established that PDGF-AA/PDGFRα binding induces PDGFRα phosphorylation and subsequently activates the PI3K–AKT signaling pathway [32]. In this study, treatment of wild-type HepG2 cells with rhPDGF-AA resulted in increased levels of p-PDGFRα, p-PI3K, and p-AKT (Fig. 6A). Functional assays confirmed that rhPDGF-AA promoted the migration of HCC cells (Fig. S9A) but had no significant effect on autophagy (Fig. S10). We next co-cultured wild-type HCC cells with ANXA3-overexpressing or ANXA3-knockout cells (Fig. 6B). The results demonstrated that ANXA3 activated the PI3K–AKT signaling pathway and enhanced *PDGFA* mRNA expression in a paracrine manner, which was abrogated by pretreatment with anti-PDGFRα (Fig. 6C, D). Collectively, these findings suggest that secreted PDGF-AA activates the PI3K–AKT signaling pathway in both HUVECs and HCC cells through paracrine and autocrine mechanisms, thereby establishing a positive feedback loop comprising PDGF-AA–PDGFRα–PI3K–AKT–ERK1/2–c-Jun–*PDGFA*.

We next investigated whether the PI3K inhibitor LY294002 could block ANXA3-mediated effects. Western blotting (Fig. 6E) and ELISA (Fig. 6F) demonstrated that ANXA3 markedly increased PDGF-AA expression, which was reversed by LY294002. Similarly, the effects of ANXA3 on cell adhesion (Fig. 6G) and migration (Fig. 6H) were abolished by PI3K inhibition. In addition, LY294002 acted as an autophagy suppressor, reducing ANXA3-induced autophagy (Fig. 6I). Overall, these results indicate that PI3K inhibition disrupts the PI3K/PDGF-AA positive feedback loop and abrogates the pro-angiogenic, pro-EMT, and pro-autophagic effects of ANXA3, thereby contributing to lenvatinib resistance in HCC.

### Alpelisib synergistically improves the antitumor effect of lenvatinib

**Fig. 7:**
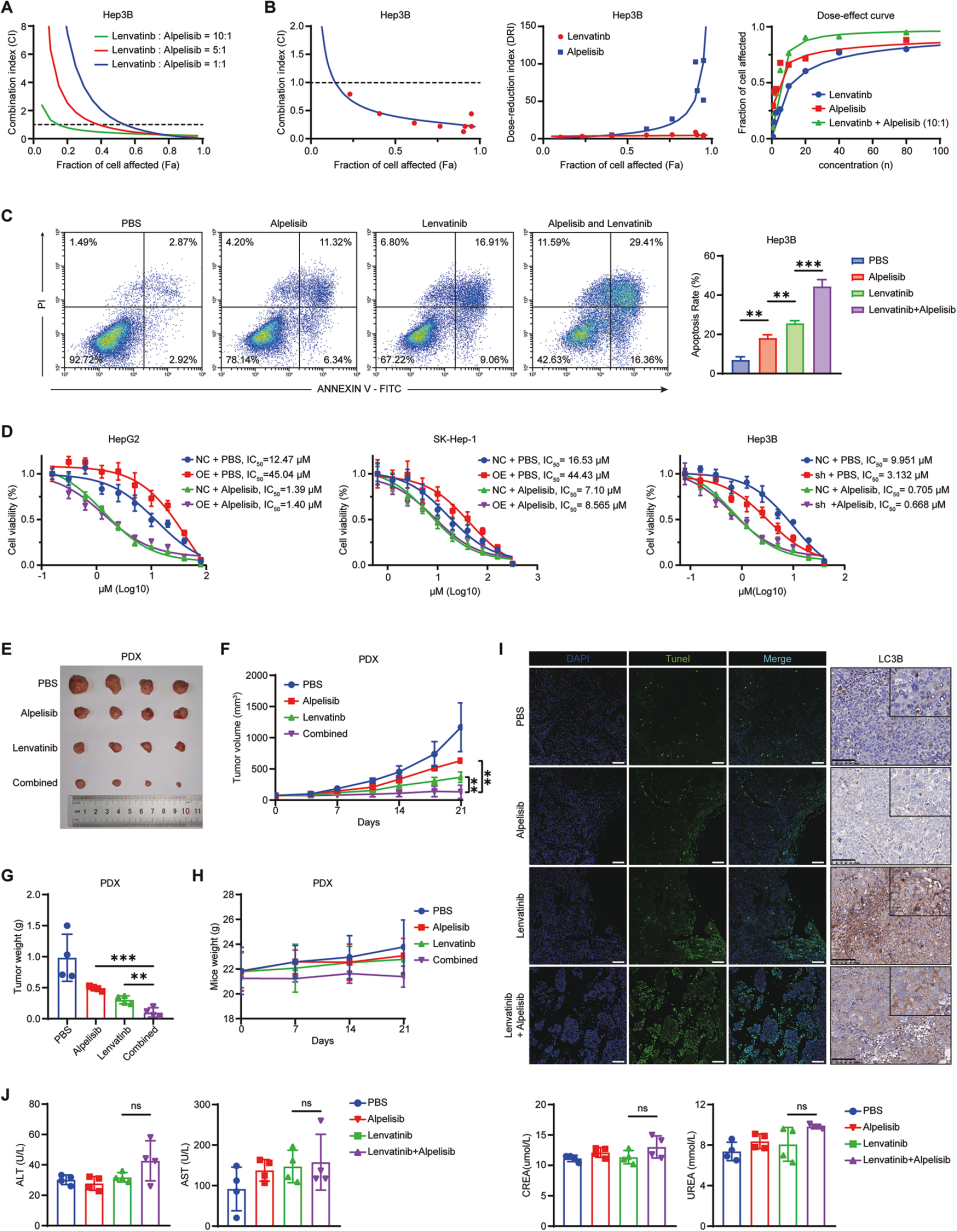
Alpelisib synergistically improves the antitumor effect of lenvatinib. **A** The combination index (CI) of lenvatinib and Alpelisib at different ratio in Hep3B cells. **B** The combination index (left), dose-reduction index (middle) and dose-effect curve (right) of lenvatinib and Alpelisib in Hep3B cells. **C** Cell apoptosis assays of Hep3B cells treated with PBS, Alpelisib, lenvatinib or combination of Alpelisib and lenvatinib. **D** The indicated cells were treated with lenvatinib at different concentrations in the presence or absence of Alpelisib for 72 h and cell viability was then measured by the CCK-8 assay. **E**–**G** Mice harboring PDX were treated with PBS, Alpelisib, lenvatinib or combination of Alpelisib and lenvatinib. The images (**E**), growth curves (**F**) and tumor weights (**G**) of the PDX from each group (*n* = 4). **H** Body weight of the mice was measured once a week (*n* = 4). **I** Representative images of TUNEL and LC3B staining in PDX in each group (*n* = 4). Scale bars, 100 μm. **J** Statistical analysis of serum ALT, AST, CREA, UREA of mice in each group (*n* = 4). The results represent three independent experiments. Error bars represent the mean ± SD. *P < 0.05; **P < 0.01; ***P < 0.001 according to Student’s *t* test.

We next investigated whether PI3K inhibition could enhance the efficacy of lenvatinib. LY294002, the first synthetic PI3K inhibitor, is widely used in in vitro studies; however, its limited clinical efficacy and poor tolerability have precluded further clinical development. Therefore, we selected Alpelisib, a specific PI3K inhibitor approved by the FDA for breast cancer treatment, to evaluate the feasibility of combination therapy in vitro. Combination index (CI) analysis revealed that Alpelisib exerted a significant synergistic effect with lenvatinib at a low dose (lenvatinib:Alpelisib = 10:1), which was superior to higher ratios (5:1 or 1:1, Figs. 7A, B and S11A–C). Furthermore, the combined treatment inhibited HCC cell migration (Fig. S11D), suppressed autophagy (Fig. S11E), and enhanced apoptosis (Fig. 7C). Subsequent assays demonstrated that Alpelisib could overcome ANXA3-induced lenvatinib resistance (Fig. 7D). Collectively, these results indicate a synergistic effect of Alpelisib and lenvatinib in vitro.

We next evaluated the effect of combined treatment with lenvatinib and Alpelisib in vivo. The patient-derived xenograft (PDX) model preserves the heterogeneity of primary tumors and the composition of the tumor microenvironment, making it a suitable model for studying tumor resistance. We established five HCC-derived PDX models and measured ANXA3 expression in each (Fig. S12). Among them, PDX-02 exhibited the highest level of ANXA3 and was selected for subsequent treatment studies. PDX-02 was derived from an HCC patient who began receiving lenvatinib treatment (8 mg QD) in April 2019. Three months later, tumor progression was observed, indicating the presence of intrinsic or acquired lenvatinib resistance.

Mice bearing HCC PDX tumors were treated with PBS, Alpelisib, lenvatinib, or a combination of Alpelisib and lenvatinib. Notably, the combination therapy exhibited the strongest antitumor effect compared with either monotherapy (Fig. 7E–G). Analysis of tumor tissues revealed increased apoptosis and decreased LC3B expression in the combination group relative to monotherapy (Fig. 7I). Moreover, combined treatment had no significant effect on body weight (Fig. 7H) or hepatic and renal toxicity (Fig. 7J). Taken together, these results suggest that the PI3K inhibitor Alpelisib synergistically enhances the antitumor efficacy of lenvatinib without exacerbating toxicity, highlighting its potential as an adjuvant therapeutic strategy for HCC patients receiving lenvatinib.

## Discussion

Lenvatinib is a multi-target tyrosine kinase inhibitor that targets vascular endothelial growth factor receptors (VEGFR1–3), platelet-derived growth factor receptor α (PDGFRα), and fibroblast growth factor receptors (FGFR1–4), as well as the proto-oncogenes RET and Kit [33]. Since 2018, lenvatinib has been approved as an alternative first-line therapy to sorafenib for patients with advanced HCC [8]. However, the clinical efficacy and durability of response to lenvatinib are frequently compromised by intrinsic or acquired resistance [34–36]. Therefore, there is an urgent need to identify reliable biomarkers that can predict therapeutic response and to develop effective strategies to enhance the efficacy of lenvatinib.

We established an orthotopic xenograft model of lenvatinib-resistant HCC, which closely recapitulates the biological characteristics of human tumors. Building upon our previous investigations of ANXA3, we found that ANXA3 was markedly upregulated in lenvatinib-resistant tumors, a finding that was further validated in a large cohort of clinical HCC specimens. Our previous studies have systematically characterized the biological functions of ANXA3 in HCC, demonstrating its roles in promoting tumorigenesis and cancer stemness [14], remodeling the immune microenvironment [13], and inducing chemoresistance [12]. Consistently, accumulating evidence from other groups has also implicated ANXA3 in tumor stemness [15], metastasis [37, 38] and autophagy [24]- processes that are closely associated with therapeutic resistance. In particular, Tong et al. reported that targeting ANXA3 with a monoclonal antibody sensitized HCC cells to sorafenib and regorafenib, primarily through p38 MAPK-mediated dysregulated autophagy [24]. However, this study mainly focused on tumor cell-intrinsic drug sensitivity and did not address the anti-angiogenic properties of multi-kinase inhibitors. It is well recognized that the antitumor efficacy of lenvatinib depends not only on its direct suppression of tumor cell growth, but also on its potent inhibition of tumor angiogenesis. Unlike previous studies, our work extends beyond tumor cell-autonomous mechanisms and comprehensively elucidates the role of ANXA3 in lenvatinib resistance from multiple pharmacological dimensions. Mechanistically, we demonstrate that ANXA3 activates the PI3K–AKT signaling pathway, thereby promoting EMT through the GSK3-β-catenin-TCF/LEF axis, while simultaneously enhancing *PDGFA* transcription via the ERK1/2-c-Jun signaling pathway. In addition, we confirmed that ANXA3 plays a critical role in facilitating lenvatinib-induced autophagy. Given that the molecular mechanism by which ANXA3 regulates autophagy has been previously elucidated [24], we did not reiterate these findings. Instead, our results further establish that ANXA3-mediated autophagy functions as a cytoprotective mechanism that partially contributes to lenvatinib resistance. Collectively, our findings indicate that ANXA3 confers lenvatinib resistance through a coordinated triad of pro-angiogenic, pro-EMT, and pro-autophagic effects, highlighting ANXA3 as a pivotal regulator of lenvatinib resistance despite not being a direct molecular target of the drug.

The PI3K–AKT signaling pathway is constitutively activated in nearly all cancer types [39–41]. In the present study, we identified the PI3K–AKT pathway as a central signaling hub that integrates multiple oncogenic effects mediated by ANXA3 in HCC. Specifically, ANXA3 activates PI3K–AKT signaling, thereby promoting EMT through the GSK3-β-catenin-TCF/LEF axis and accelerating *PDGFA* transcription via the ERK1/2–c-Jun pathway. In addition, the PI3K pathway is well recognized as a critical regulator of autophagy, and LY294002 has been widely used as a potent PI3K-dependent autophagy inhibitor. Moreover, PDGF-AA accumulated in the tumor microenvironment can bind to PDGFRα on both HUVECs and HCC cells in paracrine and autocrine manners. In HCC cells, activation of the PDGF-AA/PDGFRα axis in turn reactivates the PI3K–AKT signaling pathway, further enhancing *PDGFA* transcription. Collectively, our study identifies a previously unrecognized ANXA3-modulated positive feedback loop involving PDGF-AA–PDGFRα–PI3K–AKT–ERK1/2–c-Jun–PDGF-AA. This self-amplifying circuit markedly potentiates the role of ANXA3 in promoting tumor angiogenesis and conferring resistance to lenvatinib.

Given that activation of the PI3K signaling pathway underlies ANXA3-mediated lenvatinib resistance in HCC, we next explored whether pharmacological inhibition of PI3K could enhance the therapeutic efficacy of lenvatinib. To date, clinical trials of PI3K inhibitors have demonstrated substantial antitumor activity in chronic leukemia [42, 43], lymphoma [44–49], breast cancer [50–52], and epithelial ovarian cancer [53]. However, to our knowledge, no clinical trials have yet evaluated PI3K inhibitors in HCC. Although LY294002 is the first synthetic PI3K inhibitor and has been widely used in in vitro studies, its poor tolerability has severely limited further clinical development [54]. In contrast, Alpelisib is a selective PI3K inhibitor that has demonstrated a manageable safety profile and encouraging antitumor activity in patients with solid tumors [55, 56], and has been approved by the FDA for the treatment of breast cancer [27]. Previous studies have also reported the potential application of Alpelisib in HCC [28]. Therefore, we selected Alpelisib to evaluate the efficacy and safety of combined PI3K inhibition and lenvatinib treatment. We observed a pronounced synergistic effect between Alpelisib and lenvatinib in vitro, with a combination index (CI) < 0.3. To further validate these findings in vivo, we established a PDX model, which preserves the heterogeneity of primary tumors and the complexity of the tumor microenvironment and is widely regarded as an optimal model for studying drug resistance. Consistent with our in vitro results, combined treatment with Alpelisib and lenvatinib significantly enhanced antitumor efficacy compared with either monotherapy. Notably, Alpelisib exerted the strongest synergistic effect with lenvatinib at a relatively low dose ratio (lenvatinib:Alpelisib = 10:1), suggesting that effective tumor suppression can be achieved while minimizing Alpelisib dosage. This strategy may help reduce PI3K inhibitor–related toxicity without compromising therapeutic efficacy. Collectively, our findings provide preclinical evidence supporting the development of Alpelisib as a potential adjuvant to lenvatinib therapy in HCC, particularly in patients with high ANXA3 expression. Importantly, this work offers a rational combinatorial strategy with translational relevance and lays the groundwork for future clinical evaluation.

## Conclusion

In conclusion, we delineate the triple roles of ANXA3 in facilitating lenvatinib resistance, which are further amplified by a PI3K-mediated positive feedback loop. The PI3K inhibitor Alpelisib may synergistically enhance the antitumor efficacy of lenvatinib without increasing systemic toxicity. Our study identifies ANXA3 as a biomarker predictive of poor prognosis and lenvatinib resistance in HCC. Collectively, the combined use of Alpelisib and lenvatinib represents a promising therapeutic strategy for lenvatinib-resistant HCC.

## Supplementary information

Supplemental material

original data

original data2

## Data Availability

The datasets used and/or analyzed during the current study are available from the corresponding author on reasonable request.
