# Supplementary material for: Annexin A3 potentiates lenvatinib resistance in hepatocellular carcinoma through multiple approaches amplified by a positive feedback loop
Source: Cell Death Dis. 2026 Apr 13;17(1):478. doi: 10.1038/s41419-026-08735-9 (PMC13183909; doi:10.1038/s41419-026-08735-9)
Supplement: Supplementary file 1 — Supplemental material [file 41419_2026_8735_MOESM1_ESM.docx]

**Supplemental** **materials and methods**

**Bioinformatics analysis**

The TCGA dataset for liver hepatocellular carcinoma (LIHC) was downloaded from the UCSC Cancer Browser (<https://genome-cancer.ucsc.edu>). The mRNA expression of ANXA3 was divided into two groups according to the median value. Gene Set Enrichment Analysis (GSEA) was performed with GSEA 2.0.9 (<http://www.broadinstitute.org/gsea/>). Significantly enriched pathways were identified using Kyoto Encyclopedia of Genes and Genomes (KEGG) pathway enrichment analysis. We extracted the strong correlation signaling pathway of the ANXA3 gene in HCC according to the *P* value and false discovery rate (FDR), which were calculated by Fisher’s exact test and multiple comparison test.

**Lentivirus transduction**

The 3×Flag-tagged ANXA3 expressing and empty plasmids were purchased from Umine Biotechnology Co., Ltd. (Guangzhou, China). Three ANXA3 specific short hairpin RNA (shRNA) expression vectors and the scrambled shRNA were purchased from GenePharma Co., Ltd. (Shanghai, China). The target sequences of the three shRNAs were as follows: sh-ANXA3-1(5’-CTATGAAATCACACTCTTA-3’), shANXA3-2(5’- CAGCAGTCTTTGATGCAAA -3’), and sh-ANXA3-3(5’-GGGCACGGATGAAGACAAA-3’). The plasmids were respectively co-transfected into 293T cells with two lentiviral packaging plasmids, psPAX2 and pMD2.G, using Lipofectamine 3000 Transfection Reagent (Thermo Fisher Scientific Inc.) according to the manufacturer’s instructions. The lentivirus-containing supernatants were collected for infection to establish stably overexpressed or knockout ANXA3 HCC cells. The cells were then selected by treatment with 2 μg/mL of puromycin. The stably ANXA3-overexpressing and related control cell lines were named HepG2/OE-ANXA3, HepG2/OE-NC, SK-Hep-1/OE-ANXA3, and SK-Hep-1/OE-NC. The stable ANXA3-knockdown and related control cell lines were named Hep3B/sh-ANXA3-1, Hep3B/sh-ANXA3-2, Hep3B/sh-ANXA3-3, and Hep3B/sh-NC.

**Quantitative real-time polymerase chain reaction (qRT-PCR)**

Total RNA of HCC cells and tissues was extracted using the RNA Quick Purification Kit (ESscience, Shanghai, China) according to the manufacturer’s guideline. Reverse transcription was conducted with 1 μg total RNA using the Fast Reverse Transcription kit (ESscience). Finally, quantitative real-time PCR was performed to measure the mRNA expression with the Super SYBR Green qPCR Master Mix (ESscience). The primers applied in this study were downloaded from the PrimerBank (<https://pga.mgh.harvard.edu/primerbank/>) and verified at NCBI Primer-Blast (<https://www.ncbi.nlm.nih.gov/tools/primer-blast/index.cgi?LINK_LOC=BlastHome>). The primer sequences are listed in Table S1.

**Western Blot analysis**

Western Blot assays were performed as previously described^1^. Briefly, protein samples (20 μg) were separated by standard sodium dodecyl sulfate/polyacrylamide gel electrophoresis (SDS/PAGE) and transferred to a polyvinylidene difluoride (PVDF) membrane (Millipore Sigma). PVDF membranes were then blocked with 5% bovine serum albumin (BSA) in Tris-buffered saline containing 0.05% Tween-20 (TBST) and probed by the respective antibodies. The primary antibodies used in this experiment are listed in Table S2.

**Immunohistochemistry (IHC) and multiplex immunohistochemistry (mIHC) analysis**

The procedure of IHC was performed as previously described^1^. Multiplex immunohistochemistry (mIHC) analysis was performed using the PANO 4-plex IHC kit (Panovue, Beijing, China) according to the manufacturer’s protocol. Tissue sections were examined for the expression of ANXA3, CD34, PDGF-AA and LC3B. Images of mIHC staining were taken using the Vectra® Polaris™ Automated Quantitative Pathology Imaging System (Akoya Biosciences, Inc. Marlborough, MA, USA). The staining intensity (SI) was evaluated by multiplying the staining value (0, negative; 1, weak; 2, moderate; 3, intense) by the percentage of stained cells (0, 0%; 1, 1–25%; 2, 26–75%; 3, 75-100%). The SI level of ANXA3 was divided into the following four groups: negative (0 ≤ SI < 3), low (3≤ SI <5), medium (5≤ SI <7), and high (7≤ SI ≤9). We defined the specimens in the negative and low groups as having low ANXA3 expression, while those in the medium and high group as having high ANXA3 expression. The CD34 MVD was calculated according to the method reported by Laforga^2^. The scores were determined independently by two experienced pathologists.

Terminal deoxynucleotidyl transferase dUTP nick-end labelling (TUNEL) assays were conducted to compare the apoptosis cells using the *in situ* Cell Death Detection Kit (Millipore Sigma) according to the manufacturer’s instructions.

**ELISA**

The level of PDGF-AA in the culture medium of HCC cells was measured by the Human PDGF-AA ELISA Kit (MultiSciences, Hangzhou, China) according to the manufacturer’s instructions.

**Transwell migration and invasion assays**

Falcon® transwell chambers (Corning, New York, USA) were inserted into 24-well Falcon® Cell Culture Insert Companion Plates (Corning) and directly used for transwell migration assay. Corning® Matrigel® Invasion Chamber 24-Well Plate (Corning) was used for transwell invasion assay after rehydration with serum-free culture medium at 37℃ for 2h. Further, 5×10^4^ cells in 200 μL of serum-free culture medium were seeded into the upper chambers. The lower chamber was filled with culture medium containing 10% FBS or CM derived from the indicated HCC cells. After 12-24h of incubation, cells that migrated through the filters and adhered to the lower membrane surface were fixed with 4% paraformaldehyde, stained with 1% crystal violet, and washed with PBS. The cells in the upper chamber were gently removed using a cotton swab. The number of migrated cells was counted (five random fields per well; 100× magnification) under a phase contrast microscope and presented as the mean number of cells per field of view.

**Wound healing assay**

HUVECs were harvested and seeded into 6-well plates and incubated in a humidified incubator at 37℃ in 5% CO_2_ until 90% confluence was reached. After scratching with a 200 μL pipette tip to induce wounds, the cells were washed with PBS three times and then incubated in the CM derived from the indicated HCC cells. The wounds were photographed immediately and 24h later under an inverted microscope (Nikon ECLIPSE Ti2) and assessed by Image J software.

**Phalloidin Immunofluorescence**

HCC cells (about 5×10^5^ cells/dish) were implanted in the confocal appropriative dish and cultured in a humidified incubator at 37℃ in 5% CO_2_ for 24h. The cells were then washed three times with cold PBS, fixed in 4% paraformaldehyde, re-washed three times with cold PBS, and then incubated with the Flash Phalloidin™ Green 488 antibody (Biolegend, San Diego, CA, USA) in the dark at room temperature for 20 min. After washing with PBS, the cells were incubated with DAPI for 10 min to stain the nucleus and observed under a LSM880 with Airyscan confocal microscopy (Zeiss, Oberkochen, Germany)

**mCherry-GFP-LC3 reporter assay**

The mCherry-GFP-LC3 reporter plasmid which expressed LC3 fused with mCherry and GFP was transfected into HCC cells using Lipofectamine 3000 Transfection Reagent (Thermo Fisher Scientific Inc.). Then the cells were treated with lenvatinib for 48 h and fixed for imagination. The green fluorescent signal of GFP is sensitive to the acidic environment of lysosomes, while the red fluorescent signal of mCherry is more stable. Therefore, the yellow fluorescent puncta showing the colocalization of GFP and mCherry fluorescence indicated an autophagosome, and detection of mCherry but not GFP (red puncta) indicated an autolysosome.

**Cell adhesion assay**

Fibronectin (10 μg/mL, Sigma-Aldrich) was seeded into 96-well plate (60 μL/well). The plate was placed at 4℃ overnight for balance, hydrated with PBS, at 37℃ for 10 min, and blocked using 2% BSA in PBS at 37℃ for 30 min. Then the HCC cells (about 2.5×10^4^/well in 100 μL) were added to the pre-coated 96-well plate and cultured for 30 min at 37℃, 5% CO_2_ atmosphere. The relative adhesion level of HCC cells was analyzed by CCK-8.

**Tube formation assay**

Matrigel (10 μL/well, BD Biosciences, San Jose, CA, USA) was placed into the inner well of μ-Slide Angiogenesis (ibidi GmbH, Planegg, Germany) plates and solidified in a humidified incubator at 37℃ for 30 min. Then, HUVECs were separately suspended in CM of the indicated HCC cells at a density of 4×10^5^ cells/mL and seeded into the pre-coated Matrigel wells (50 μL/well). After continuous incubation for 6h, tube formation was observed with an inverted microscope (Nikon), and the tube formation ability of HUVECs was assessed by Image J Angiogenesis Analyzer.

**Apoptosis assay**

Cell apoptosis was analyzed using the Annexin V-APC/7-AAD Apoptosis kits (ESscience) according to the manufacturer’s instructions. Briefly, after exposure to DMSO or lenvatinib for 48h, cells were harvested and stained with Annexin V-APC and 7-AAD at 4℃ for 15 min in the dark. Apoptosis rates were analyzed by flow cytometry within 1h.

**Human angiogenesis array**

The Proteome Profiler Human Angiogenesis Array Kit (ARY007, R&D Systems, Inc. USA & Canada) was used to detect the relative levels of 55 human angiogenesis-related proteins according to the manufacturer’s instructions. Briefly, culture medium derived from Hep3B cells with or without ANXA3 knockdown was mixed with a cocktail of biotinylated detection antibodies and then incubated with capture antibodies spotted in duplicate on nitrocellulose membranes. Captured proteins were visualized using chemiluminescent detection reagents. The spot signals were quantified using ImageJ software.

**Human phospho-kinase antibody array**

Phosphorylation of 43 human kinases and total amounts of 2 related proteins in Hep3B cells with or without ANXA3 knockdown were detected using the Proteome Profiler Human Phospho-Kinase Array Kit (ARY003C). Briefly, 500 μg of cell lysate was incubated with the array membrane, which was spotted in duplicate, with capture antibodies to specific target proteins at 4℃ overnight. Phosphorylation of captured proteins was detected using biotinylated phospho-specific detection antibodies and then visualized with chemiluminescent detection reagents. The spot signals were quantified using ImageJ software.

**Dual luciferase reporter assay**

For TOP/FOP luciferase reporter assay, cells (1×10^5^ cells/well) were seeded in 96-well plates and co-transfected with pRL-TK Renilla plasmid and either TOP-Flash or FOP-Flash luciferase reporter. For luciferase reporter assay that detected the transcription activity of c-Fos/c-Jun on the promoter of *PDGFA*, cells were transfected pcDNA3.1-c-Fos, pcDNA3.1-c-Jun, and pGL4.10-*PDGFA*-promoter together with pRL-TK as an internal control. After 48h, the luciferase activity was measured with the Dual-Luciferase Reporter Assay System (Promega, CA, USA) according to the manufacturer’s instructions.

**Chromatin immunoprecipitation (ChIP) assay**

The ChIP assay was performed using the SimpleChIP® Enzymatic Chromatin IP Kit (Cell Signaling Technology, Danvers, Massachusetts, USA) according to the manufacturer’s instructions. Anti-c-Fos and anti-c-Jun (Cell Signaling Technology) antibodies were used to immunoprecipitate the chromatin in HCC cells. qPCR was performed using the following primers identified for the three c-Fos/c-Jun binding sites in the PDGF promoter region: Site A forward 5’- GAGAGACGTGGGGAGGGGG-3’ and reverse 5’- TTGGGTGAGCAGCGGAGAA-3’; Site B forward 5’- GATGCCTGCACCGCTTTCG-3’ and reverse 5’- TGCTTCCCTCGGCTCCCCT-3’; Site C forward 5’- ACCGACTAATAGCGGCTGGAA -3’and reverse 5’- TGGGGGGATTGGGCGTTGAAA -3’.

**Combination index**

The effects of drug combinations were evaluated by calculating the combination index (CI) using CompuSyn software (ComboSyn Inc, NJ, USA). According to previous reports, combination effects were defined as follows: CI ≤ 0.7, synergistic effect; 0.7 < CI < 1.0, slight synergistic/additive effect; and CI > 1.0, antagonistic effect.

**Animal models**

We established an orthotopic xenograft model of lenvatinib-resistant HCC by spleen inferior pole injection. Specifically, we generated lenvatinib-resistant HCC cells by exposing the HCC cell lines to increasing concentrations of lenvatinib. After about 6 months of culture, we obtained lenvatinib-resistant HCC cells that could grow stably and passage under 100 μM of lenvatinib. Testing of the half maximal inhibitory concentration (IC_50_) value was used to confirm the successful construction of lenvatinib-resistant cell lines. Then we randomly assigned 10 BALB/c nude mice into 2 groups and injected the prepared HCC cell suspension into the inferior pole of the mouse’s spleen. After 1 weeks, we started to treat the mice with lenvatinib (10mg/(kg·d), orally) for another 3 weeks. All mice were euthanized and their livers were collected for imagination, tumor counting and IHC analysis.

For the subcutaneous tumor model, BALB/c nude mice were randomly assigned into 4 groups of 7 mice per group and then subcutaneously injected with the indicated cells (3×10^6^) suspended in 100 μL of PBS containing 50% Matrigel Basement Membrane Matrix (BD Biosciences). Tumor volumes were measured twice a week using a vernier caliper and calculated using the following formula: volume (mm^3^) = 0.5 × L × W^2^ with L representing the largest and W representing the smallest diameters, respectively. When the tumors reached 25 mm^3^ in size, all mice were treated with lenvatinib (10 mg/(kg·d), orally). All mice were finally euthanized and the tumors were embedded in paraffin. Serial 4.0 mm sections were obtained and analyzed by mIHC analysis with DAPI, anti-ANXA3, and anti-CD34 antibodies, as well as TUNEL assays.

For construction of the metastasis model, BALB/c nude mice were randomly assigned into 4 groups of 5 mice per group and then injected with the indicated cells (1× 10^6^ cells in 100 μL PBS) that carrying bioluminescence via tail vein injection. Tumor metastasis was monitored at day 42 using an *in vivo* imaging system (PerkinElmer, IVISLumina Series III, USA) and all mice were sacrificed after that. The number of metastatic nodules in each lung were counted. H&E staining was performed to detect the number of micro-metastatic pulmonary nodules.

In order to evaluate the synergistic effect of Alpelisib and lenvatinib in *vivo*, we constructed PDX models of HCC. Specifically, surgical specimens of HCC patients were cut into 3mm^3^ pieces and transplanted into 4-week-old NCG mice. The mice were sacrificed when the transplanted tumors reached 1cm^3^. The subcutaneous transplanted tumors were collected, cut into small pieces, and transplanted into the second batch mice. Drug administration began when the tumors reached 25mm^3^ in size, at which point mice were randomized for treatment with PBS, Alpelisib (1mg/(kg·d), orally), lenvatinib (10mg/(kg·d), orally) or combination of Alpelisib (1mg/(kg·d), orally) and lenvatinib (10mg/(kg·d), orally). Tumor volumes were recorded twice a week and mouse weights were measured every week. All mice were euthanized at 3 weeks after drug administration and the tumors were harvested, photographed, weighed. We also measured the concentrations of aspartate aminotransferase (AST), creatinine (CREA), alanine aminotransferase (ALT) and blood urea nitrogen (BUN) in the serum.

**Statistical analysis**

Sample size estimation: The sample sizes for animal experiments (n = 5 per group) were determined based on previous similar studies in our laboratory and preliminary experiments, which indicated that this number would provide adequate power (80%) to detect a 30% difference in tumor volume with α = 0.05. For in vitro experiments, triplicate wells were used in each experiment, and all assays were repeated independently at least three times.

Randomization and blinding: Animals were randomly assigned to experimental groups using a random number generator. No formal blinding was performed during the experiments or outcome assessment due to the exploratory nature of this study.

Data were expressed as the mean ± standard deviation (SD). Survival analysis was conducted using the Kaplan-Meier method. The χ2 test was performed to analyze the relationship between ANXA3 expression and the clinicopathological characteristics. Based on the variables selected by univariate analysis, the multivariate Cox proportional hazards model was used to determine the independent prognostic factors of HCC. The association between ANXA3 and CD34 MVD, or PDGF-AA expression in HCC tissues was calculated using the Pearson correlation test. The differences between the groups were evaluated using the student’s two-tailed t test and one-way analysis of variance (ANOVA). *P<*0.05 was considered statistically significant. The statistical analysis and figure generation were performed using the Prism 8.0 software (GraphPad Software, Inc., San Diego, CA, USA) and SPSS version 23.0 (IBM Corporation, Armonk, NY, USA).

In the figures, the error bars represent the mean ± SD. **P* < 0.05; ***P* < 0.01; ****P* < 0.001; *****P* < 0.0001; ns means non-significant.

**References**

1. Huang Y, Zhu Y, Yang J, Pan Q, Zhao J, Song M*, et al.* CMTM6 inhibits tumor growth and reverses chemoresistance by preventing ubiquitination of p21 in hepatocellular carcinoma. *Cell Death Dis* 2022, **13**(3)**:** 251.

2. Laforga JB, Aranda FI. Angiogenic Index: A New Method for Assessing Microvascularity in Breast Carcinoma with Possible Prognostic Implications. *Breast J* 2000, **6**(2)**:** 103-107.

**Supplemental Figures**


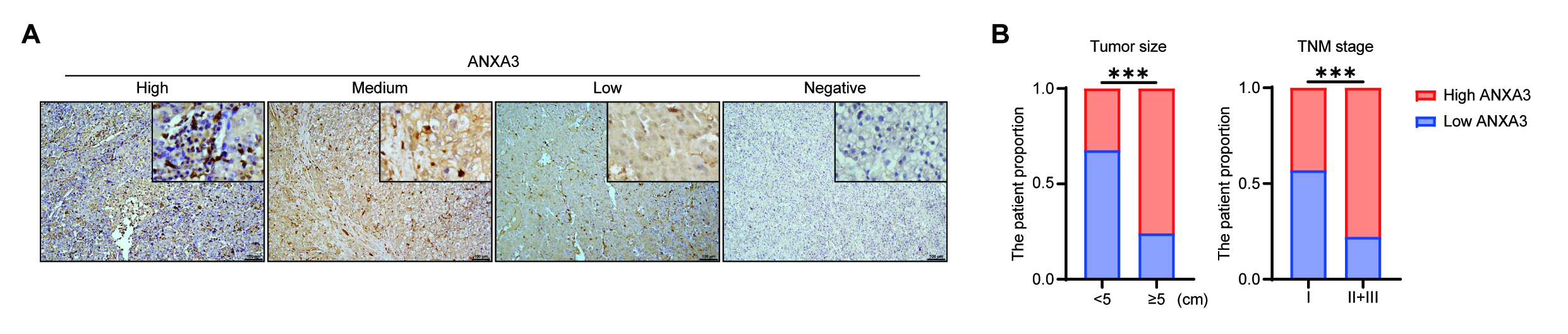


**Fig S1. A.** Typical IHC images of ANXA3 expression status in 149 HCC tissues. Scale bars, 100 μm. **B.** Comparison of the percentages of ANXA3 expression status according to tumor sise and TNM stage (n=149).


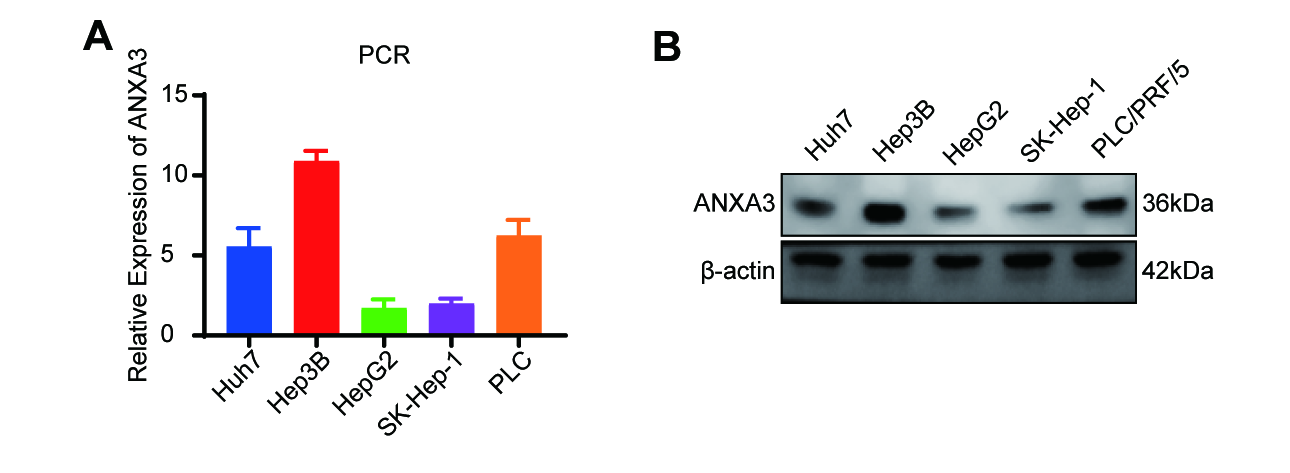


**Fig S2**. **A.** qRT–PCR analysis of ANXA3 expression in HCC cells. **B.** Western Blot analysis of ANXA3 expression in HCC cells. The results represent three independent experiments. Error bars represent the mean ± SD.


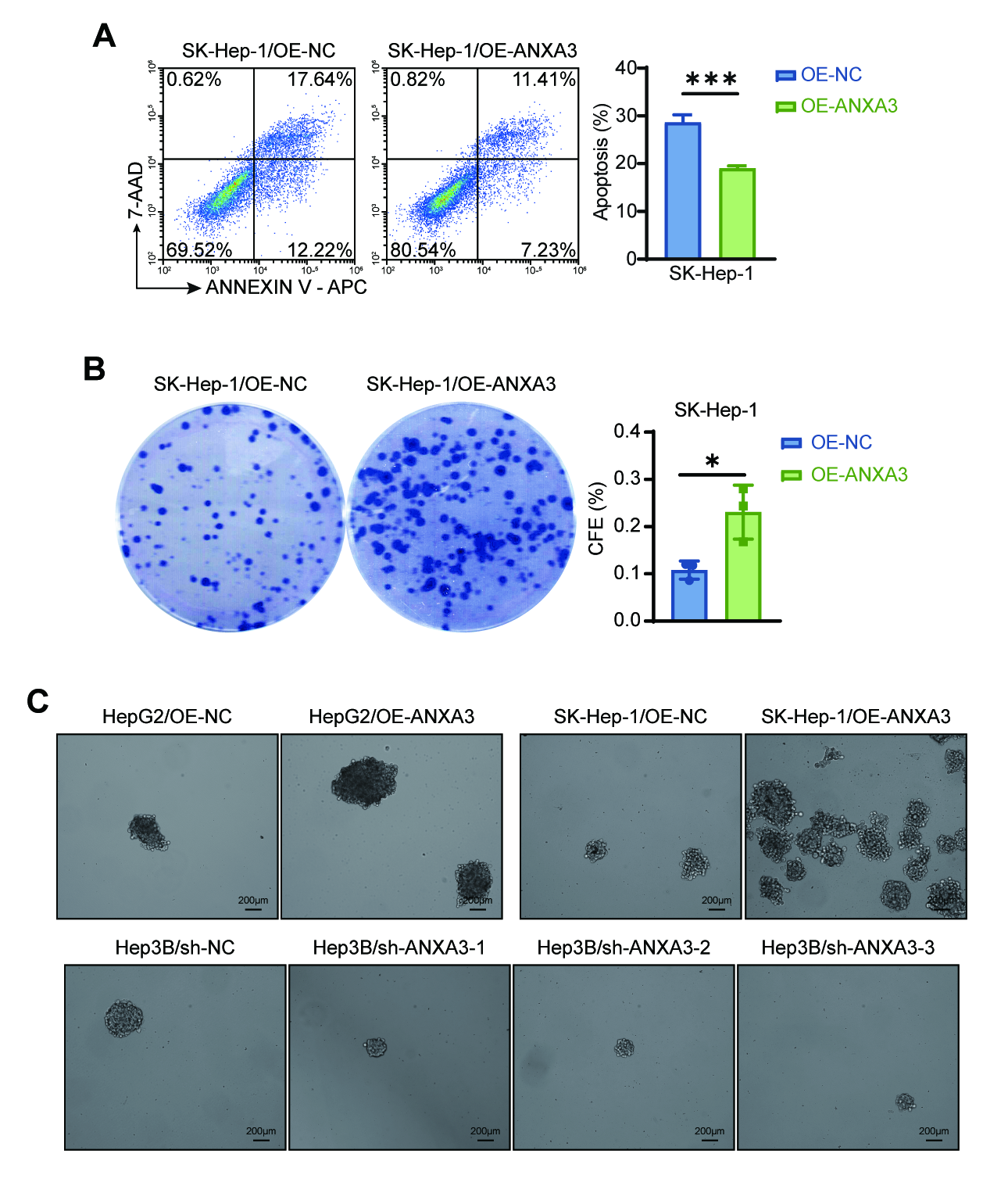


**Fig S3**. **A.** The cell apoptosis rates of the indicated HCC cells which were treated with lenvatinib for 72h. **B**. Colony formation of the indicated HCC cells following lenvatinib treatment. **C.** Sphere formation of the indicated HCC cells following lenvatinib treatment. Scale bars, 200 μm. The results represent three independent experiments. Error bars represent the mean ± SD. *p < 0.05; **p < 0.01; ***p < 0.001 according to Student’s t-test.


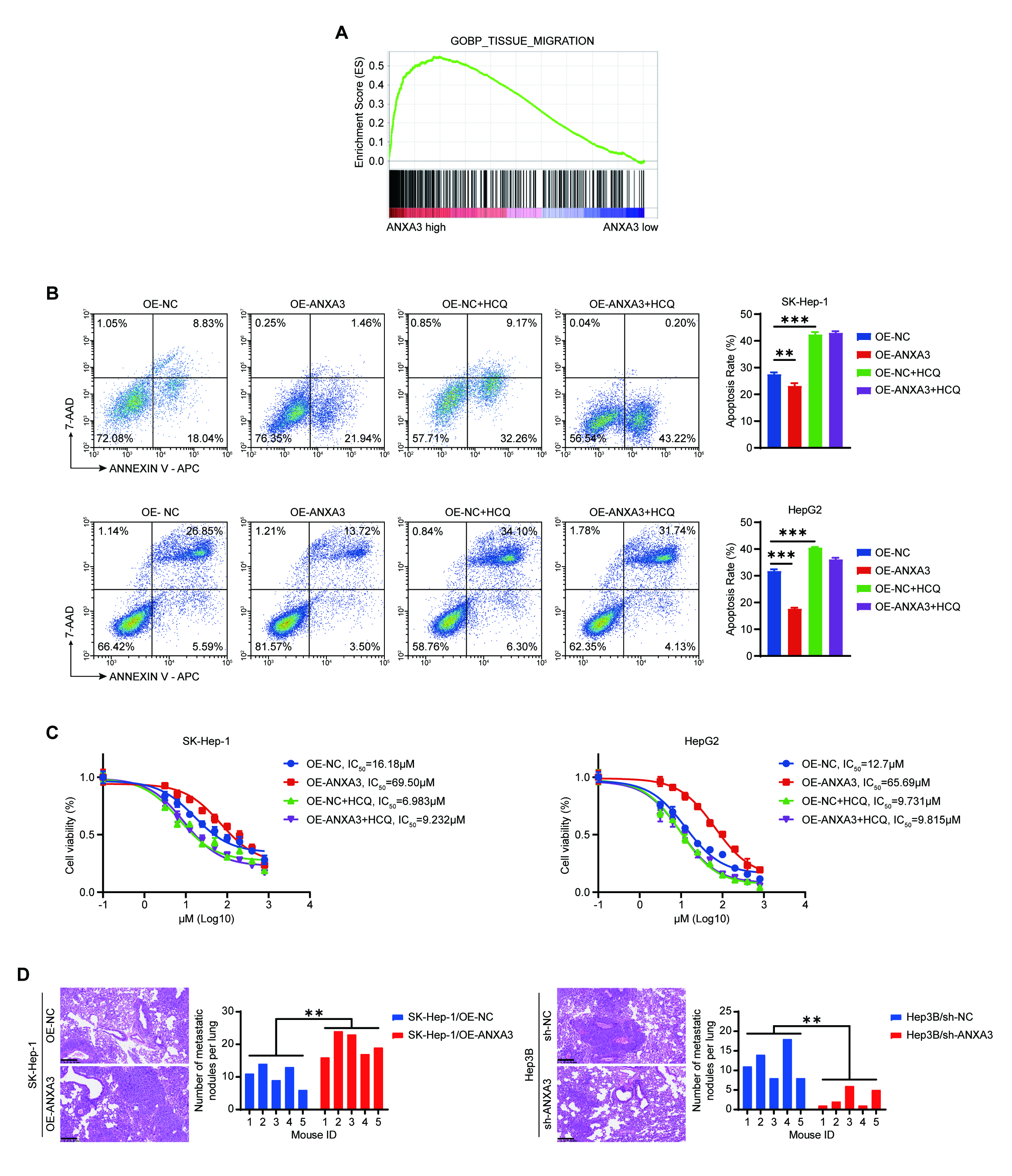


**Fig S4**. **A.** GSEA showing that ANXA3 expression is positively correlated with tissue migration.

**B.** The cell apoptosis rates of the indicated HCC cells after lenvatinib treatment for 72 h in the presence or absence of the hydroxychloroquine (HCQ).

**C.** The IC_50_ value of the indicated HCC cells in the presence or absence of HCQ.

**D.** H&E staining and tumor number statistical analysis of lung metastatic tumors in the tested mice. Scale bars, 200 μm (n=5).

The results represent three independent experiments. Error bars represent the mean ± SD. *p < 0.05; **p < 0.01; ***p < 0.001 according to Student’s t-test.
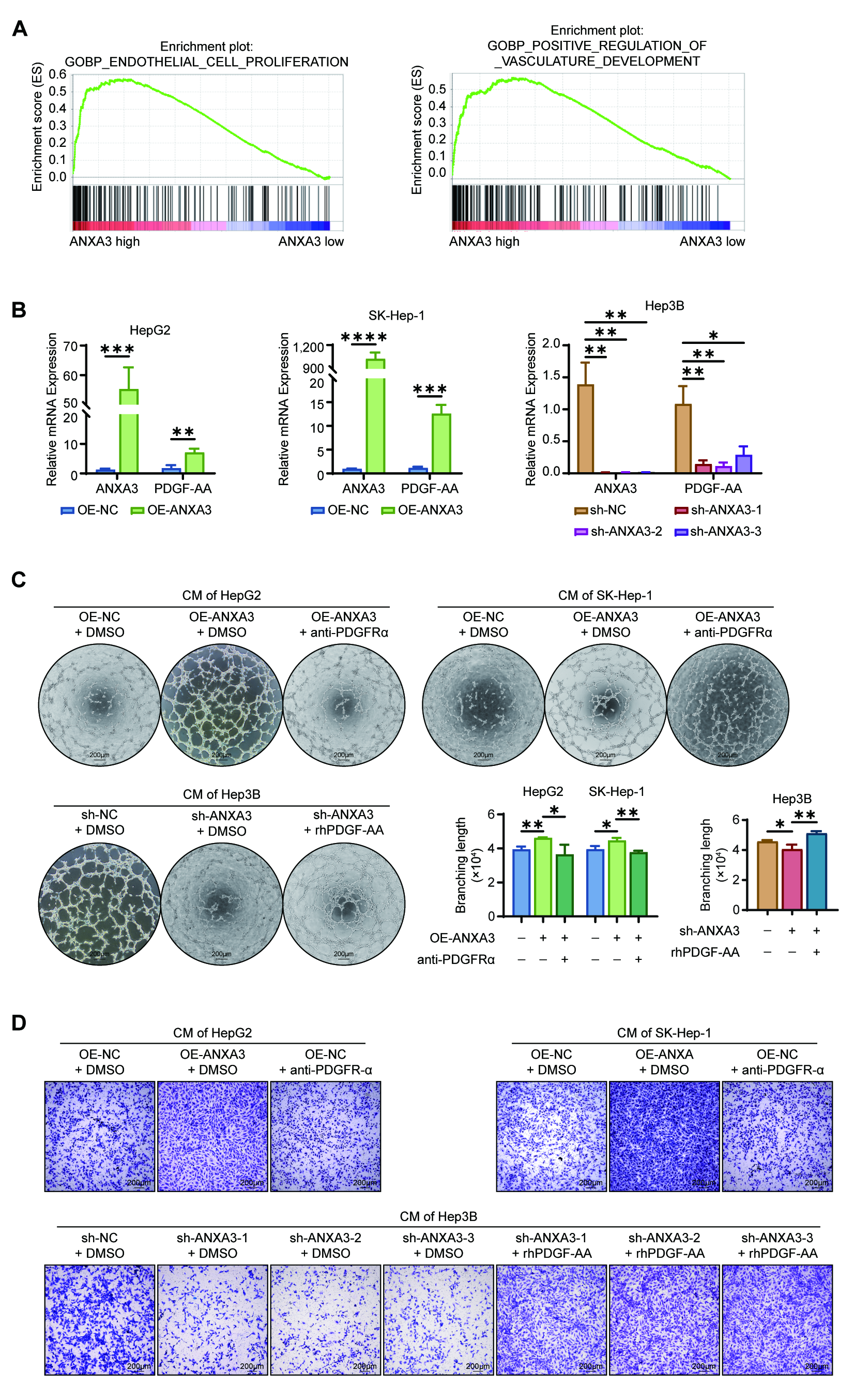


**Fig S5**. **A.** GSEA showing that ANXA3 expression is positively correlated with endothelial cell proliferation and positive regulation of vasculature development.

**B.** qPCR assays showing the influence of ANXA3 regulation on the PDGFA mRNA expression.

**C.** The CM of ANXA3-overexpressing cells were applied to perform tube formation assays on HUVECs with or without anti-PDGFRα (top). The CM of ANXA3-knockout cells were applied to perform tube formation assays on HUVECs in the absence or presence of rhPDGF-AA (bottom). Scale bars, 200 μm.

**D.** The CM of HepG2 and SK-Hep-1 cells with ANXA3-overexpressing were applied to perform transwell assays on HUVECs with or without anti-PDGFRα (top). The CM of Hep3B cells with ANXA3-knockout were applied to perform transwell assays on HUVECs in the absence or presence of rhPDGF-AA (bottom). Scale bars, 200 μm.

The results represent three independent experiments. Error bars represent the mean ± SD. *p < 0.05; **p < 0.01; ***p < 0.001 according to Student’s t-test.


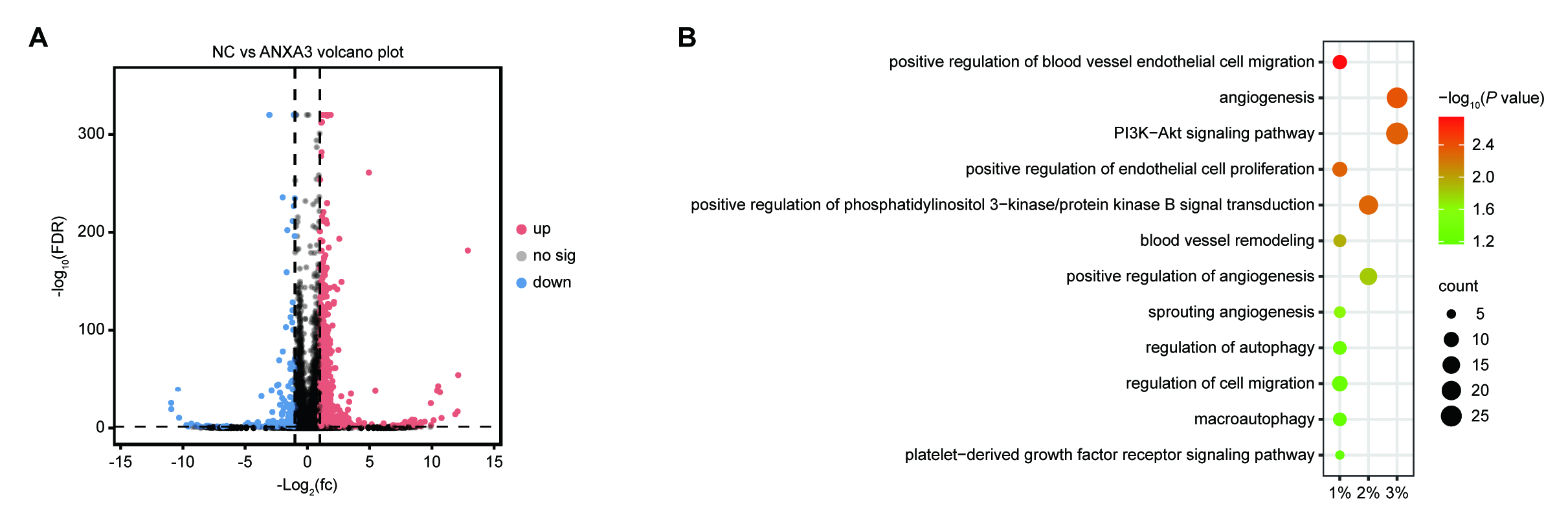


**Fig S6**. **A.** Volcano plot of differentially expressed genes identified by RNA sequencing in Hep3B cells following ANXA3 knockdown. **B.** Pathway enrichment analysis revealing the association of ANXA3 with angiogenesis, autophagy, metastasis, and the PI3K signaling pathway.


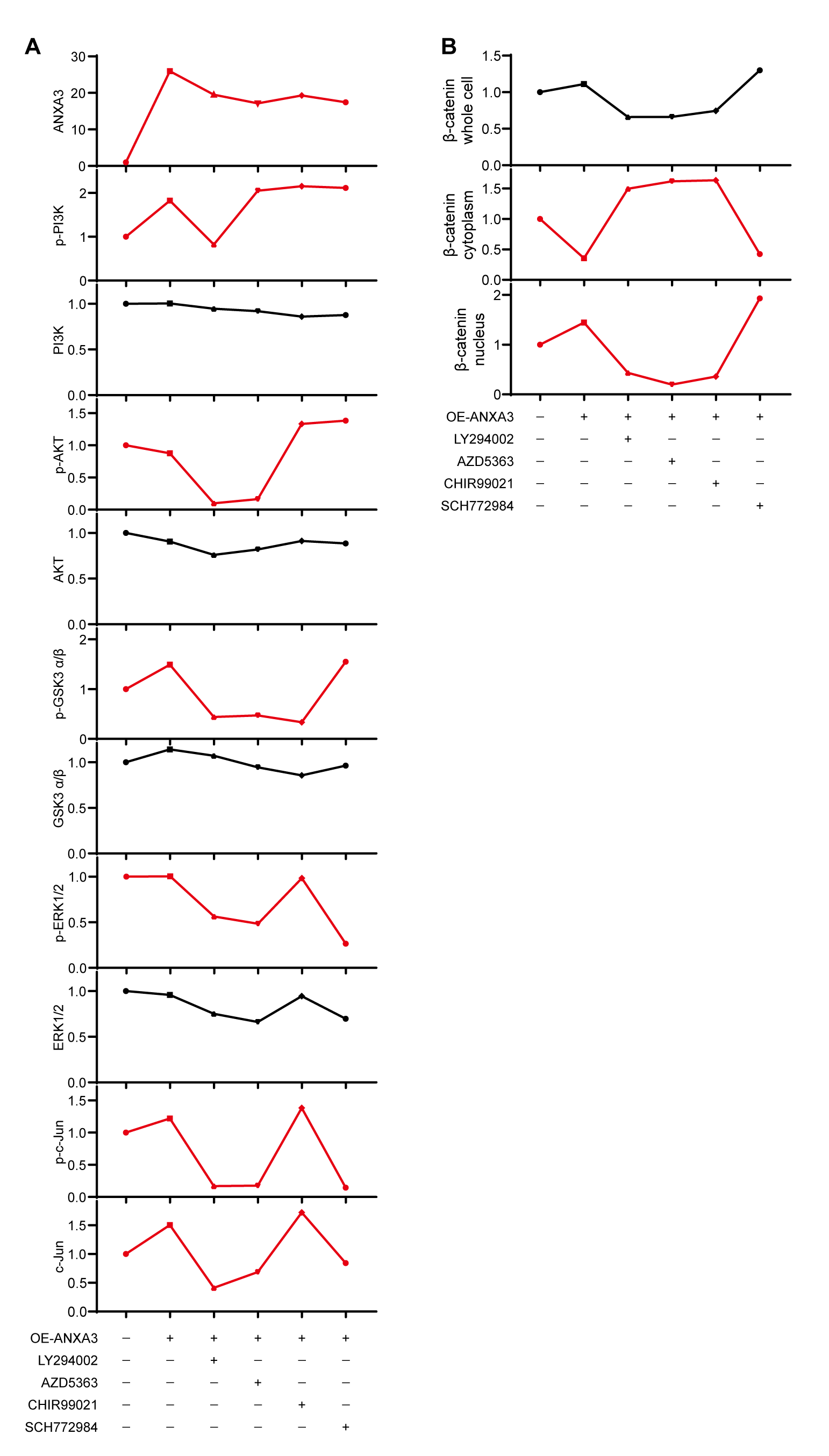


**Fig S7**. **A.** Line charts showing the expression of the indicated proteins in whole-cell lysates of HepG2/OE-ANXA3 cells treated with DMSO, LY294002 (PI3K inhibitor), AZD5363 (Akt inhibitor), CHIR99021 (GSK3α/β inhibitor), or SCH772984 (ERK1/2 inhibitor). **B.** Line charts showing the expression of β-catenin in whole-cell, cytoplasmic, and nuclear fractions.


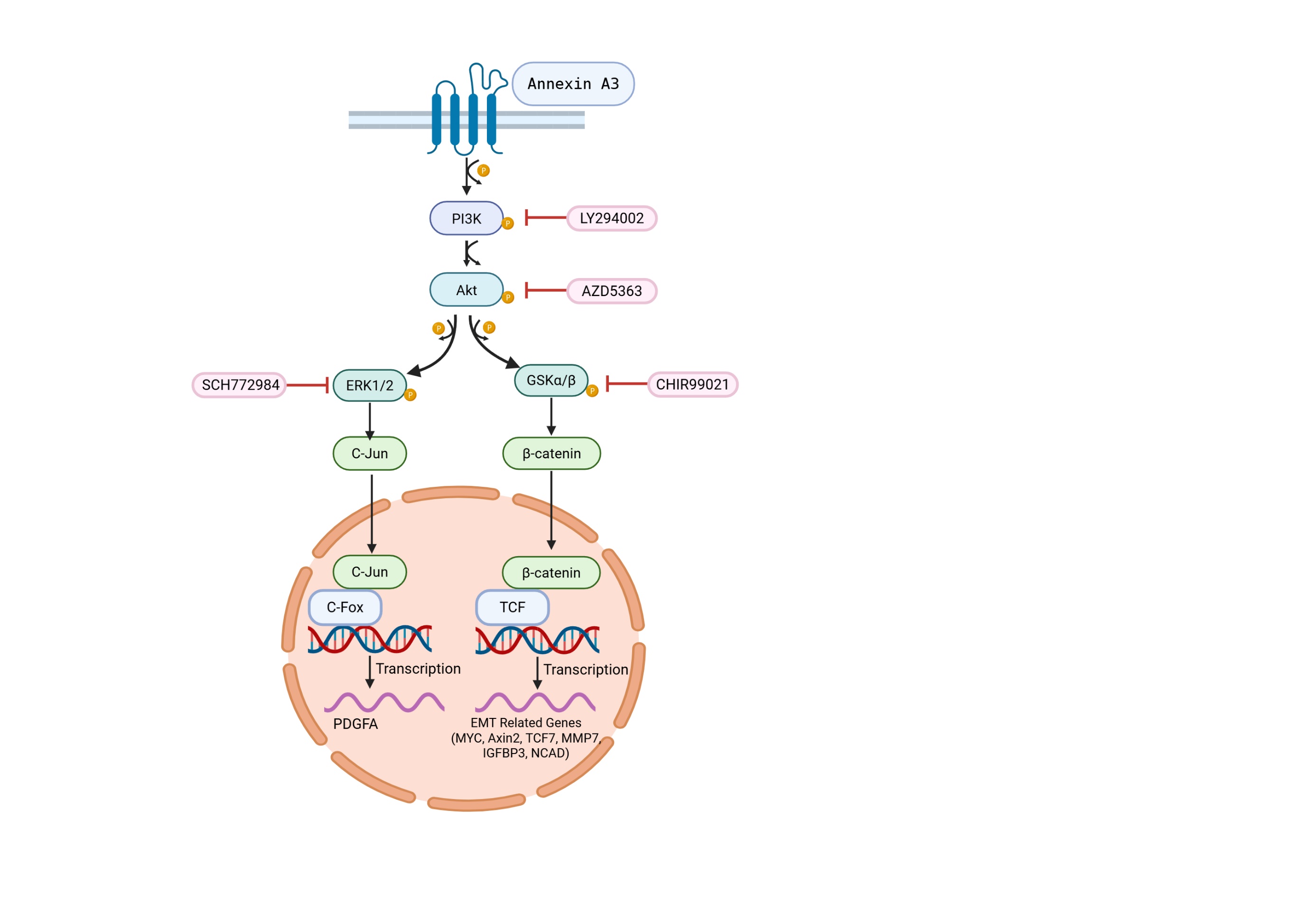


**Fig S8**. Schematic illustration of the molecular mechanism by which ANXA3 promotes transcription of PDGF-AA and EMT-related genes.


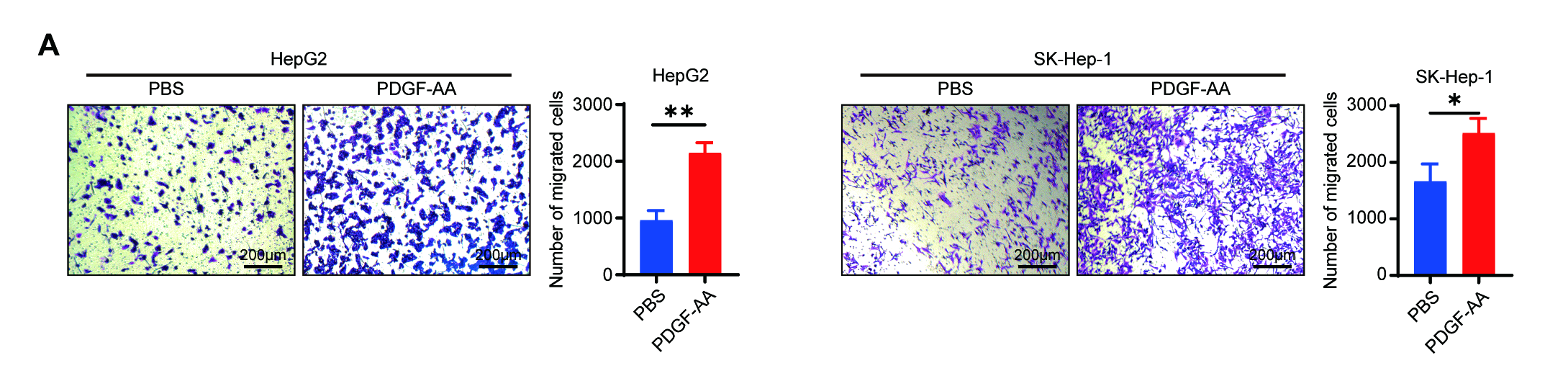


**Fig S9**. **A.** Transwell assays showing the migratory capacities of the indicated cells following lenvatinib treatment in the presence or absence of PDGF-AA. Scale bars, 200 μm. The results represent three independent experiments. Error bars represent the mean ± SD. *p < 0.05; **p < 0.01; ***p < 0.001 according to Student’s t-test.


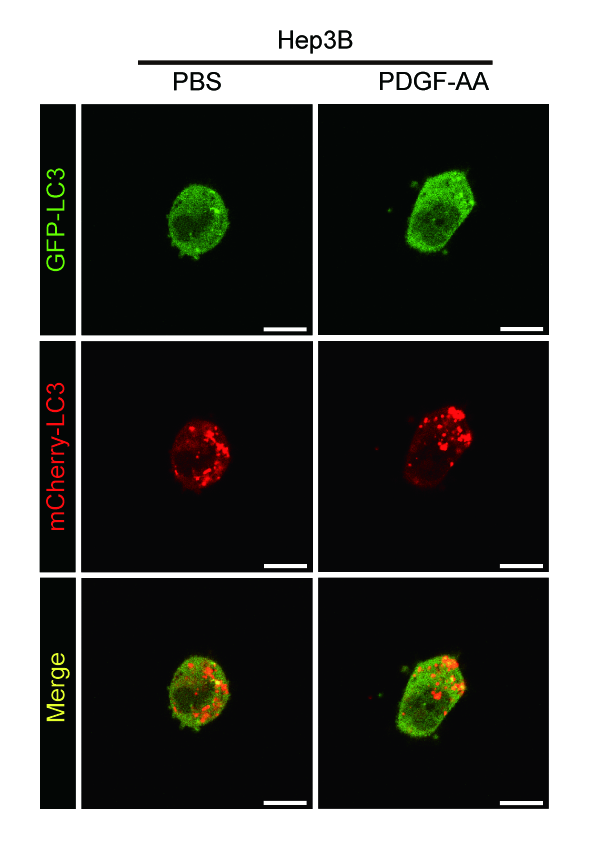


**Fig S10**. Immunofluorescence analysis showing autophagic flux in the indicated cells following lenvatinib treatment in the presence or absence of PDGF-AA. Scale bars, 10 μm. The results represent three independent experiments.


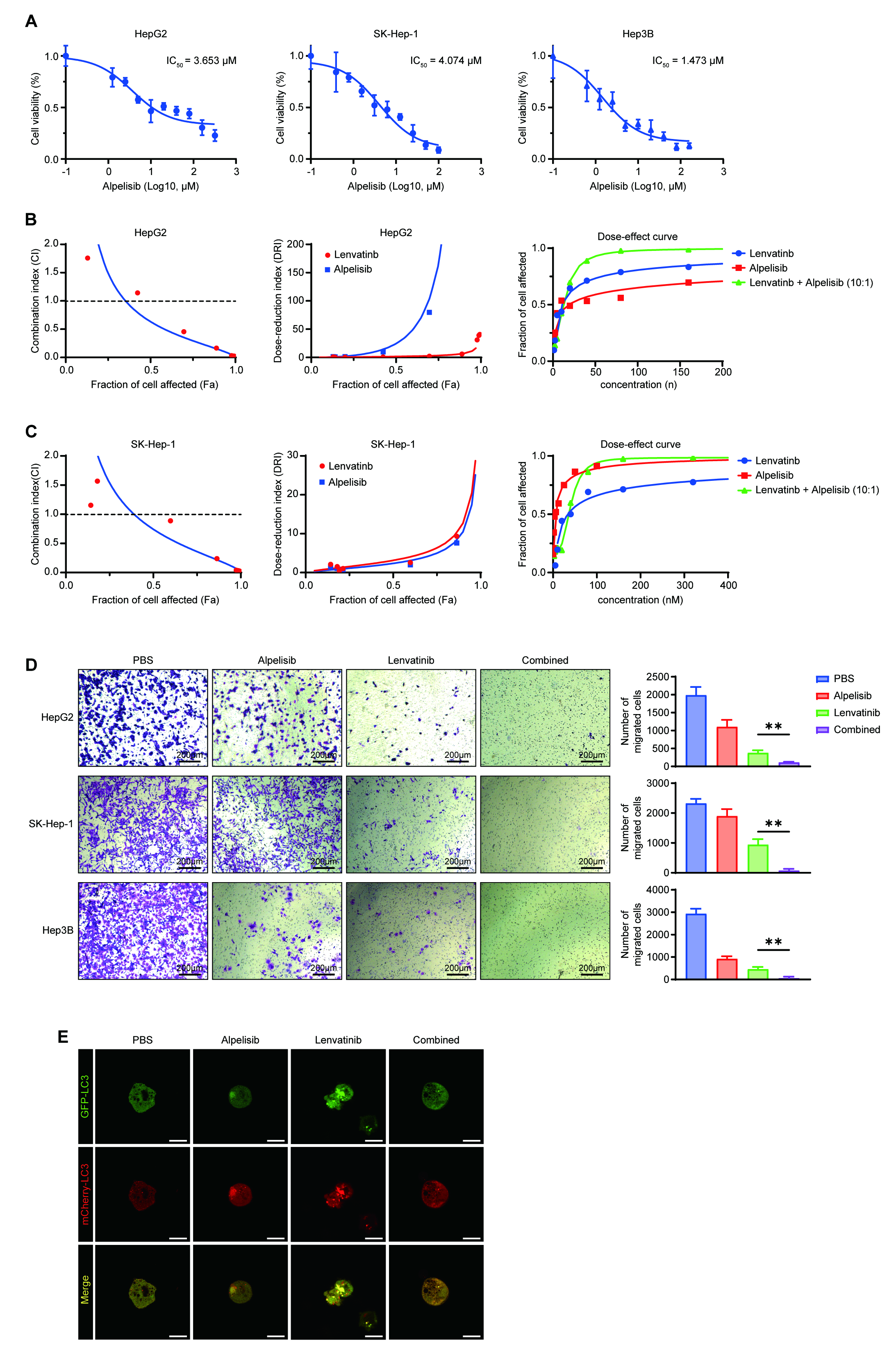


**Fig S11**. **A.** IC₅₀ values of Alpelisib in HepG2, SK-Hep-1, and Hep3B cells.

**B.** Combination index (CI), dose-reduction index (DRI), and dose–effect curves of lenvatinib and alpelisib in HepG2 cells.

**C.** Combination index (CI), dose-reduction index (DRI), and dose–effect curves of lenvatinib and alpelisib in SK-Hep-1 cells.

**D.** Transwell assays showing the migratory capacity of the indicated cells following different treatments. Scale bars, 200 μm.

**E**. Immunofluorescence analysis showing autophagic flux in the indicated cells following different treatments. Scale bars, 10 μm.

The results represent three independent experiments. Error bars represent the mean ± SD. *p < 0.05; **p < 0.01; ***p < 0.001 according to Student’s t-test.


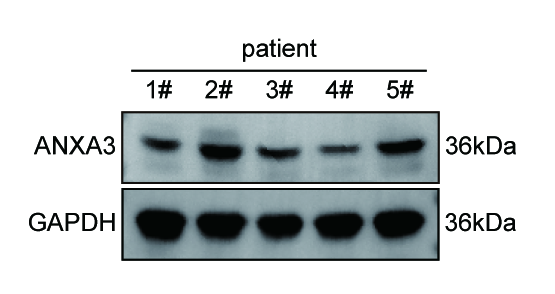


**Fig S12**. Western blotting of ANXA3 in PDX models established from HCC patient tumors.

**Supplemental Tables**

Table S1. The sequences of the primers used in this study

| Primer |  | 5’-3’ Sequence |
| --- | --- | --- |
| ANXA3 | F | TTAGCCCATCAGTGGATGCTG |
|  | R | CTGTGCATTTGACCTCTCAGT |
| PDGF-AA | F | GCAAGACCAGGACGGTCATTT |
|  | R | GGCACTTGACACTGCTCGT |
| E-cadherin | F | CGAGAGCTACACGTTCACGG |
|  | R | GGGTGTCGAGGGAAAAATAGG |
| claudin-1 | F | CCTCCTGGGAGTGATAGCAAT |
|  | R | GGCAACTAAAATAGCCAGACCT |
| ZO-1 | F | ACCAGTAAGTCGTCCTGATCC |
|  | R | TCGGCCAAATCTTCTCACTCC |
| N-cadherin | F | TGCGGTACAGTGTAACTGGG |
|  | R | GAAACCGGGCTATCTGCTCG |
| Vimentin | F | AGTCCACTGAGTACCGGAGAC |
|  | R | CATTTCACGCATCTGGCGTTC |
| Snail | F | ACTGCAACAAGGAATACCTCAG |
|  | R | GCACTGGTACTTCTTGACATCTG |
| Slug | F | TGTGACAAGGAATATGTGAGCC |
|  | R | TGAGCCCTCAGATTTGACCTG |
| ZEB1 | F | CAGCTTGATACCTGTGAATGGG |
|  | R | TATCTGTGGTCGTGTGGGACT |
| ZEB2 | F | GCGATGGTCATGCAGTCAG |
|  | R | CAGGTGGCAGGTCATTTTCTT |
| Fibronectin | F | GAGAATAAGCTGTACCATCGCAA |
|  | R | CGACCACATAGGAAGTCCCAG |
| c-Myc | F | GTCAAGAGGCGAACACACAAC |
|  | R | TTGGACGGACAGGATGTATGC |
| Axin2 | F | TACACTCCTTATTGGGCGATCA |
|  | R | TTGGCTACTCGTAAAGTTTTGGT |
| TCF7 | F | TTGATGCTAGGTTCTGGTGTACC |
|  | R | CCTTGGACTCTGCTTGTGTC |
| MMP7 | F | GAGTGAGCTACAGTGGGAACA |
|  | R | CTATGACGCGGGAGTTTAACAT |
| IGFBP3 | F | AGACACACTGAATCACCTGAAGT |
|  | R | AGGGCGACACTGCTTTTTCTT |
| MMP3 | F | CTGGACTCCGACACTCTGGA |
|  | R | CAGGAAAGGTTCTGAAGTGACC |
| PDGFRα | F | TGCGGGTGGACTCTGATAATGC |
|  | R | GTGGAACTACTGGAACCTGTCTCG |
| PDGFRβ | F | AGACACGGGAGAATACTTTTGC |
|  | R | AGTTCCTCGGCATCATTAGGG |
| VEGFR-1 | F | TTTGCCTGAAATGGTGAGTAAGG |
|  | R | TGGTTTGCTTGAGCTGTGTTC |
| VEGFR-2 | F | AACGTGTCACTTTGTGCAAGA |
|  | R | TTCCATGAGACGGACTCAGAA |
| VEGFR-3 | F | TGCACGAGGTACATGCCAAC |
|  | R | GCTGCTCAAAGTCTCTCACGAA |
| FGFR-1 | F | GGCTACAAGGTCCGTTATGCC |
|  | R | GATGCTGCCGTACTCATTCTC |
| FGFR-2 | F | GGTGGCTGAAAAACGGGAAG |
|  | R | AGATGGGACCACACTTTCCATA |
| FGFR-3 | F | TGCGTCGTGGAGAACAAGTTT |
|  | R | GCACGGTAACGTAGGGTGTG |
| FGFR-4 | F | CCATAGGGACCCCTCGAATAG |
|  | R | CAGCGGAACTTGACGGTGT |

Table S2. The primary antibodies used in this study

| Name of the antibody | Catalog number | Application | Dilution | Brand of the antibody |
| --- | --- | --- | --- | --- |
| anti-ANXA3 | 11804-1-AP | WB | 1:1000 | Proteintech |
| anti-ANXA3 | TA502058 | IHC, mIHC | 1:200 | Origene |
| anti-GAPDH | 10494-1-AP | WB | 1:5000 | Proteintech |
| anti-β-actin | 66009-1-IG | WB | 1:5000 | Proteintech |
| anti-CD34 | ab81289 | IHC, mIHC | 1:3000 | Abcam |
| Alexa Fluor 488 Phalloidin | 8878 | IF | 1:20 | Cell Signaling Technology |
| DAPI | C0065-10 | IF | 1:1 | Solarbio |
| anti-E-cadherin | 3195 | WB | 1:1000 | Cell Signaling Technology |
| anti-N-cadherin | 13116 | WB | 1:1000 | Cell Signaling Technology |
| anti-Vimentin | 5741 | WB | 1:1000 | Cell Signaling Technology |
| anti-LC3B | 3868 | WB | 1:1000 | Cell Signaling Technology |
| anti-LC3B | 14600-1-AP | IHC | 1:200 | Proteintech |
| anti-PDGF-AA | ab203911 | WB | 1:1000 | Abcam |
| anti-PDGF-AA | sc-9974 | mIHC | 1:50 | Santa Cruz |
| anti-PI3K | 4292 | WB | 1:1000 | Cell Signaling Technology |
| anti-phospho-PI3K | 17366 | WB | 1:1000 | Cell Signaling Technology |
| anti-AKT | 4685 | WB | 1:1000 | Cell Signaling Technology |
| anti-phospho-AKT | 4060 | WB | 1:1000 | Cell Signaling Technology |
| anti-GSK 3α/β | 5676 | WB | 1:1000 | Cell Signaling Technology |
| anti-phospho-GSK 3α/β | 8566 | WB | 1:1000 | Cell Signaling Technology |
| anti-ERK 1/2 | 4695 | WB | 1:1000 | Cell Signaling Technology |
| anti-phospho-ERK 1/2 | 4370 | WB | 1:1000 | Cell Signaling Technology |
| anti-c-Jun | 9165 | WB | 1:1000 | Cell Signaling Technology |
| anti-phospho-c-Jun | 91952 | WB | 1:1000 | Cell Signaling Technology |
| anti-β-catenin | 8480 | WB | 1:1000 | Cell Signaling Technology |
| anti-TBP | 44059 | WB | 1:1000 | Cell Signaling Technology |
| anti-PDGFRA | MAB322 | WB, | 1:500 | R&D SYSTEMS |
|  |  | FC | 1:50 |  |
|  |  | Neutralization | 1:50 |  |

Table S3. Association between ANXA3 expression and the clinicopathological features of HCC

| Clinicopathologic variables |  | ANXA3 expression | | *X^2^* | *P* value |
| --- | --- | --- | --- | --- | --- |
|  |  | low | high |  |  |
| All case |  | 63 | 86 |  |  |
| Gender | Male | 54 | 65 | 2.322 | 0.128 |
|  | Female | 9 | 21 |  |  |
| Age | ＜50 | 39 | 47 | 0.784 | 0.376 |
|  | ≥50 | 24 | 39 |  |  |
| HBsAg | Positive | 58 | 75 | 0.894 | 0.344 |
|  | Negative | 5 | 11 |  |  |
| AFP (ng/mL) | ≤400 | 34 | 51 | 0.422 | 0.516 |
|  | ＞400 | 29 | 35 |  |  |
| Cirrhosis | Negative | 55 | 68 | 1.711 | 0.191 |
|  | Positive | 8 | 18 |  |  |
| Tumor size(cm) | ＜5 | 42 | 20 | 28.204 | 0.000 |
|  | ≥5 | 21 | 66 |  |  |
| Tumor number | single | 58 | 69 | 4.044 | 0.044 |
|  | multiple | 5 | 17 |  |  |
| Tumor thrombus | Negative | 62 | 73 | 7.818 | 0.005 |
|  | Positive | 1 | 13 |  |  |
| Micro satellite | Negative | 59 | 64 | 9.337 | 0.002 |
|  | Positive | 4 | 22 |  |  |
| Histological grade | 1 | 15 | 21 | 2.101 | 0.350 |
|  | 2 | 39 | 45 |  |  |
|  | 3 | 9 | 20 |  |  |
| Metastasis | Negative | 63 | 82 | 3.011 | 0.083 |
|  | Positive | 0 | 4 |  |  |
| T stage | I | 49 | 37 | 17.997 | 0.000 |
|  | II+III | 14 | 49 |  |  |

Table S4. Univariate and multivariate analyses of factors associated with overall survival (OS)

|  | Univariate analysis | | | Multivariate analysis | | |
| --- | --- | --- | --- | --- | --- | --- |
|  | HR | 95%CI | *P* value | HR | 95%CI | *P* value |
| ANXA3 expression | 2.487 | 1.583~3.907 | 0.000 | 2.249 | 1.383~3.658 | 0.001 |
| Gender | 1.167 | 0.679~2.008 | 0.576 |  |  |  |
| Age | 1.267 | 0.833~1.925 | 0.268 |  |  |  |
| HBsAg | 0.901 | 0.452~1.796 | 0.767 |  |  |  |
| AFP | 0.733 | 0.473~1.136 | 0.165 |  |  |  |
| Cirrhosis | 0.941 | 0.540~1.641 | 0.831 |  |  |  |
| Tumor size | 1.583 | 1.030~2.435 | 0.036 | 1.137 | 0.718~1.803 | 0.584 |
| Tumor number | 1.547 | 0.899~2.661 | 0.115 |  |  |  |
| Tumor thrombus | 1.800 | 0.901~3.595 | 0.096 |  |  |  |
| Micro satellite | 1.839 | 1.091~3.101 | 0.022 | 1.428 | 0.836~2.442 | 0.192 |
| Histological grade | 1.321 | 0.958~1.821 | 0.089 |  |  |  |
| Metastasis | 2.497 | 0.909~6.855 | 0.076 |  |  |  |

Table S5. Univariate and multivariate analyses of factors associated with disease free survival (DFS)

|  | Univariate analysis | | | Multivariate analysis | | |
| --- | --- | --- | --- | --- | --- | --- |
|  | HR | 95%CI | *P* value | HR | 95%CI | *P* value |
| ANXA3 expression | 1.623 | 1.107~2.378 | 0.013 | 1.327 | 0.878~2.004 | 0.179 |
| Gender | 0.894 | 0.565~1.416 | 0.633 |  |  |  |
| Age | 1.513 | 1.041~2.197 | 0.030 | 1.599 | 1.091~2.344 | 0.016 |
| HBsAg | 0.665 | 0.364~1.217 | 0.186 |  |  |  |
| AFP | 1.078 | 0.739~1.571 | 0.697 |  |  |  |
| Cirrhosis | 0.840 | 0.511~1.379 | 0.490 |  |  |  |
| Tumor size | 1.542 | 1.052~2.260 | 0.026 | 1.453 | 0.958~2.202 | 0.078 |
| Tumor number | 1.425 | 0.860~2.363 | 0.169 |  |  |  |
| Tumor thrombus | 1.393 | 0.726~2.670 | 0.319 |  |  |  |
| Micro satellite | 1.778 | 1.099~2.875 | 0.019 | 1.506 | 0.913~2.485 | 0.109 |
| Histological grade | 1.288 | 0.969~1.713 | 0.081 |  |  |  |
| Metastasis | 1.643 | 0.604~4.470 | 0.331 |  |  |  |

Table S6. The putative promoter region of *PDGFA*

>NC_000007.14:c522668-520569 Homo sapiens chromosome 7, GRCh38.p13 Primary Assembly

AGCATGGGGTGCGAATGTTTCGGGGGCGGCGCGCGGGGCGCGTCTGCCTCCCGCACAAAGCCAGGCTCTGTCCCGGGGTCAGCGCCTCTGTCTCCCGGGCCTGGGCGGGGACGCCCACGCCTCCTCCGACGCGGGAGTATATTTAGCCTCATTATCAAATGTTTGCAGTGAACTGTGTGAACCGACTAATAGCGGCTGGAAGTGAAACTCGATTCCTGGCCAGAAAGAAGCATGTGACCGCCCCCGTCTGCAAGGATCGCGGCAGGGCGGGCGGGGCAGGTGCCGCAGCCTCCGCGCGCAAAACCCGCCGGCGGGGCCCGGGTCGCACCGTCCCCTCCTGGGCCGAGCACCTGAAACCCACCTCGTCCCTCCGTCCCCCTCCCCAGGGGCTGCAATGAGGACGCTGGGAGTTTGAATGGAAACGAAGGCCGGGGAGGGGGGTCCGCGCGGGCGTGGGTTTTTTATCCAAGTCGGTGACCTCTTGGTTAAGTCAGCCCTGCGGTTTCAACGCCCAATCCCCCCAAAAAAGCCTGTGGGGGTCCACGTCTCCTTGTGTCTTCCGGGGATCCGGCGGAGAATGTGGCGGGGCCGGGCAGGGGGGTCCCAGTCCCACGCCGGGTGCGTCGTGCCCCGAGTCTCCCCAGAAGGCGGCTGTCGCGCGGCTGCACAAGTTTTCGCCTCCCTCGCGCGGCCGCTGTTGTTGTGGTCGCCATGGCGACGGGTCGCGGTTTTATTTTTAATAGCGGCCGGCGATTAGAGAGATGCCTGCACCGCTTTCGCCTAAGCTCTGCGTCCCCCGCGGGTGAAGCACCCACGCCCCGCCCCAGCCCCCGCCTCGCTCCAGCGCCCCTTGCACGTCCCCAGGTGGCCCTAAACTCTAGCTGGGCGCACCCGTAGCTCCAGGCCGGGCAGGGGAGCCGAGGGAAGCAGCCATCTAGGCCCTCAGAGGCGGCGAGGACCCCCAGGATCCCTCAAGCTGGGAGGGACGGGTACCCCATCCCCAATACAACGCGACCTTCCTCCTTGCCTTCCACACCGTCTTACCGAGTCCTCTAACCCCACAGAGGTGTCCTCGGGCCCCCTCTGCGCGCGAGCAGTCCGAGGCCCAGGAAGACAGGATGCGCGCCACTCCGGGATAACTGGCCGAAGAATGAGGCCGGGGTCCGGCCTGCGTCCTGGCCCTGCGAGTCCGGCTCTTTCCAGAAGCCATGCAGTGGGTGTCCCCTCCCAAGCCGCAAGTTCCCCCTGGAGGGACAAGGCCTGGCTTTAAGGGGCTCCTCCACGTTTCCAAGGCTCCCCTGGTTCTGTCCCGGTCCCGCTTGGTCCTGACCACCCAGAGGGCATGCTTGACCGCGGGGGTCGTGGTGTGGGGCTGCGGCCAACGGAAGGGAGAGACGTGGGGAGGGGGCCTGCAGGTGTGTGGAAGGGCGCGTGTAGACGCGACCCACTGGGAGCGGAGGGAGAGGCGTCGCTCCCCAAATATTTGGGGGAAGGGGAGAGCCCCCGAAGCGCTCTCCGGATTCGGCCTTTGAAATGCTAGAGCTGGCCTAGCTGGGGATGAAAGACCGCCCGCGGGGCTGCGCCTTAACGGCCTTTGTCTGGAGGGAGGGCGACCGCGACGGGGGCAGGGGTTTAATCGACAGGGTTGGGGGTGACGGCTGGGCCGGGCGGGATTCTCCGCTGCTCACCCAAGGAAGCGTCTCCTGGGCTCCAGGGGCCTAGGCCGTCTAGCCTCGGGGTCTGCACAGCACGCCACCCCGCTGGACCCGGTACCAGGACGCGTAGACCCCCTGGCAGGGCACTGACGAGCACCTGGACCTGTAGTCAGGGTCCAATCCCTCCTGGTCTAGGGAGGCGAAATCCATCCAGCCCAGGCGCACCCGCCCTTTCTCGGCGTGGGAGCCGTCTAGCCACGCTCAGACCTCGGCCTCCGCGCGCGTTCCTCCCCCGCATCGCGCTCTGAGCTGCGCCTCGGCCGGAGGTGGCGCTCTGTGCGCCCCACGGATCCCGACGCGGGAGGCTCAGAACCCGGGGGACCCTCACCGGTGGCTCCTTTCCCTTCCATCCCCTCGACTTCCCGGCTCAGGGCGCGGCCCTGCC
