# Supplementary material for: Annexin A3 potentiates lenvatinib resistance in hepatocellular carcinoma through multiple approaches amplified by a positive feedback loop
Source: Cell Death Dis. 2026 Apr 13;17(1):478. doi: 10.1038/s41419-026-08735-9 (PMC13183909; doi:10.1038/s41419-026-08735-9)
Supplement: Supplementary file 2 — original data [file 41419_2026_8735_MOESM2_ESM.docx]

Fig2 A

Left:

ANXA3


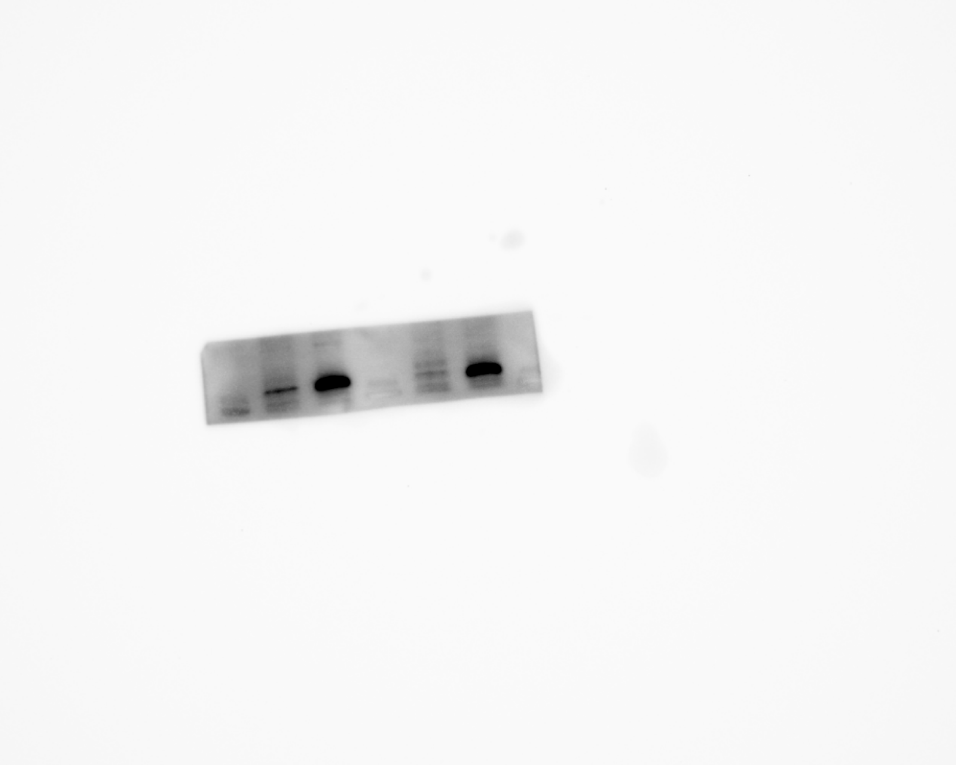


β-actin


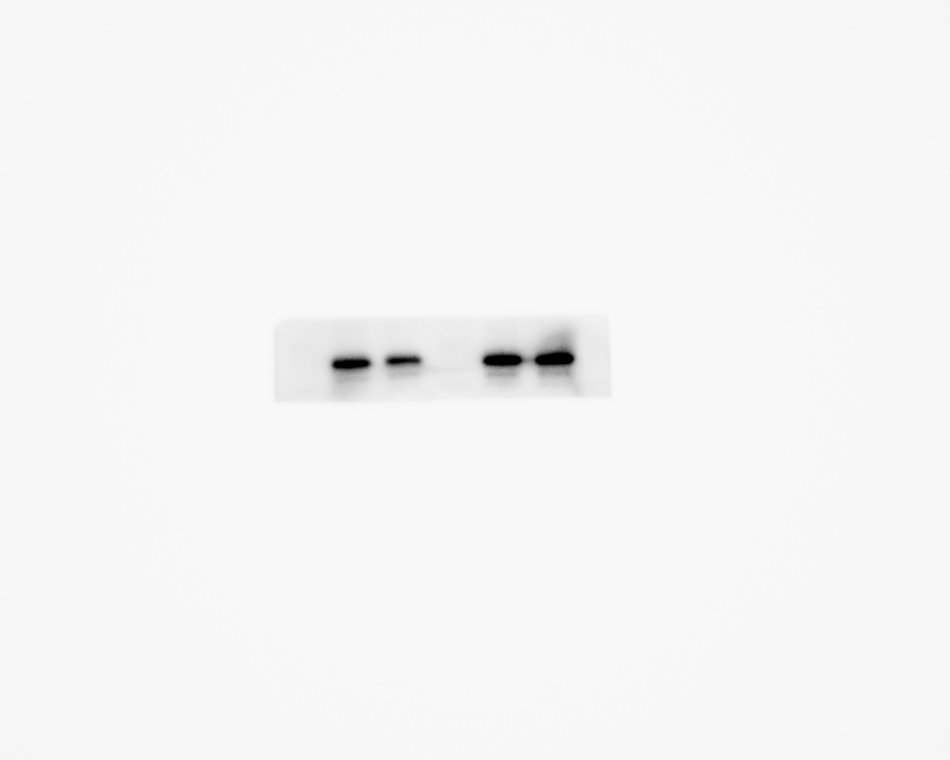


Right:

ANXA3


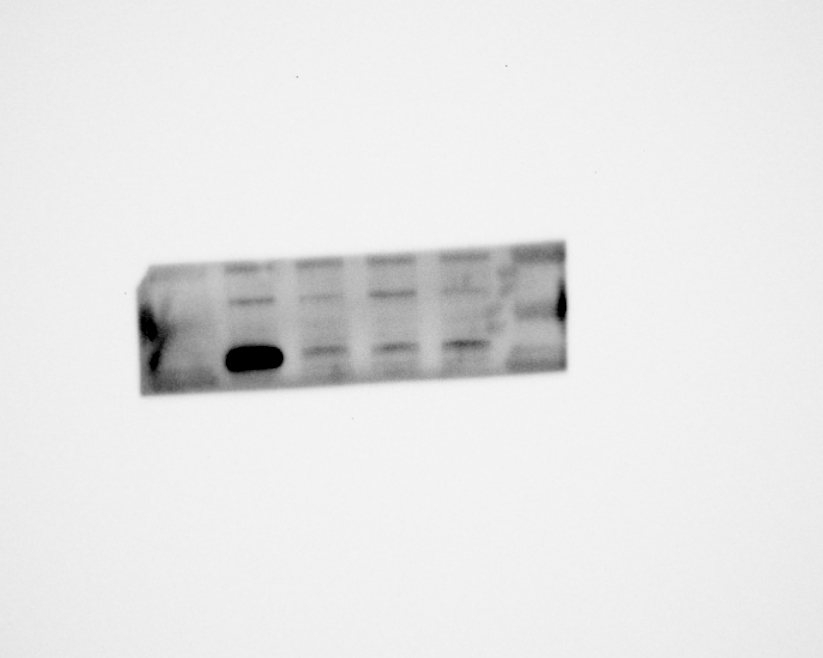


β-actin


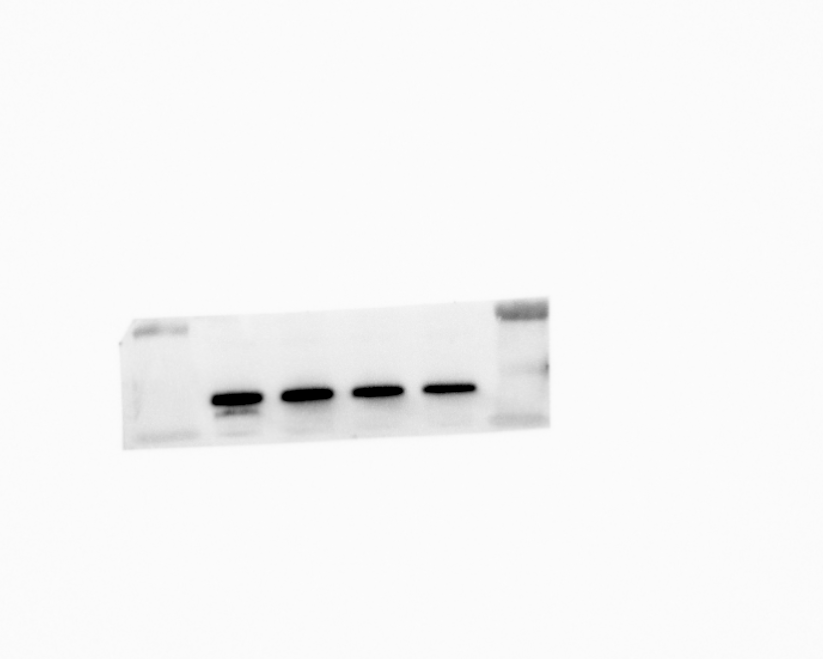


FigS2A

ANXA3 36KD,


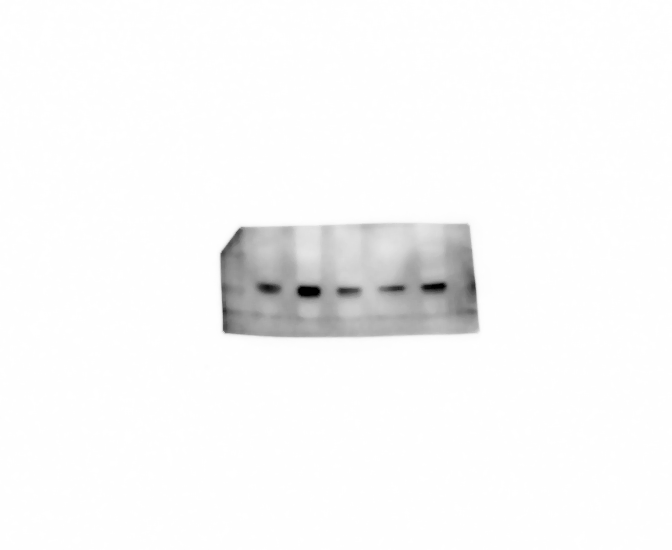


β-actin


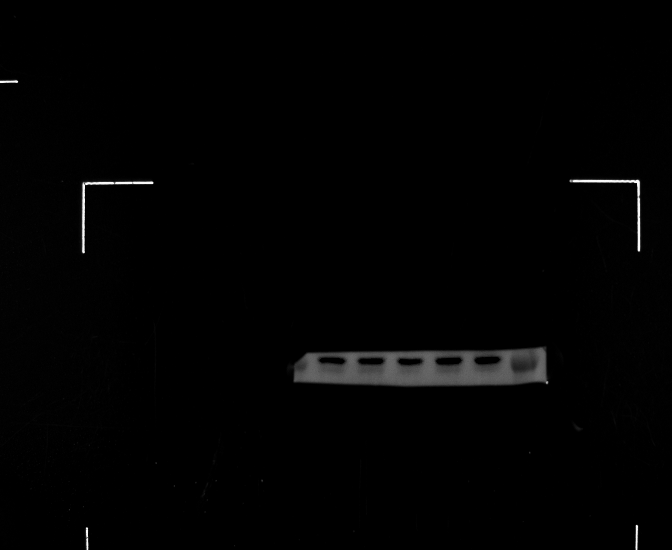


Fig3E

Left, Top

ANXA3 36KD,


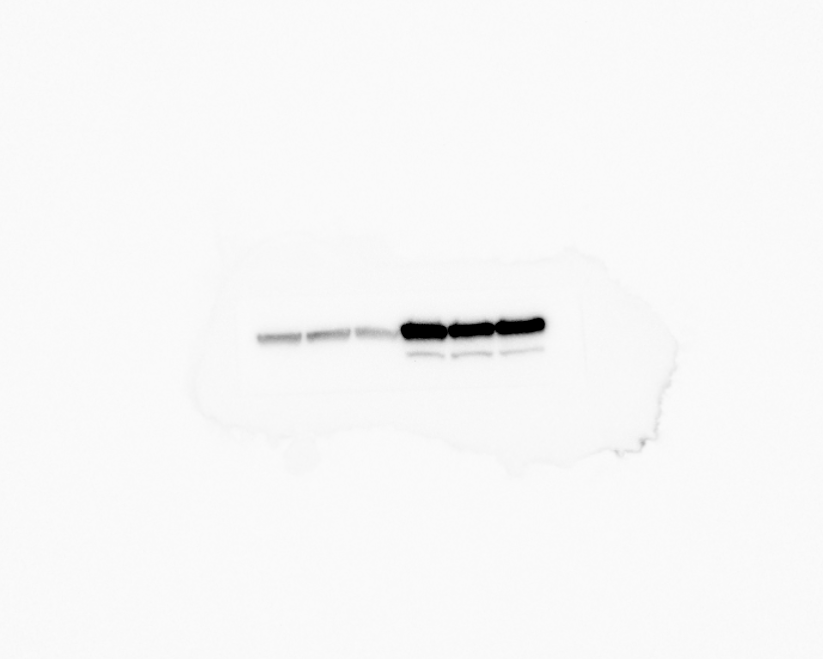


LC3B:


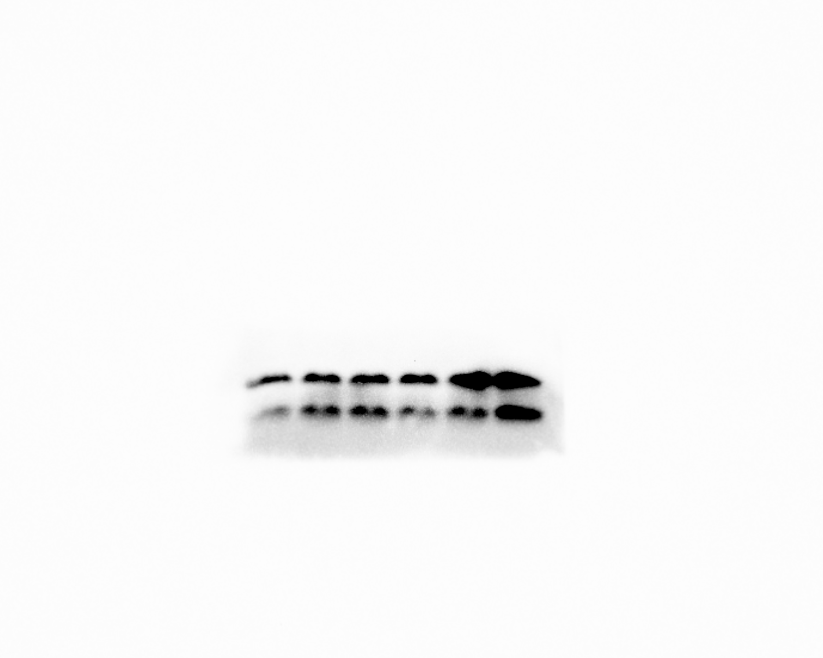


GAPDH:


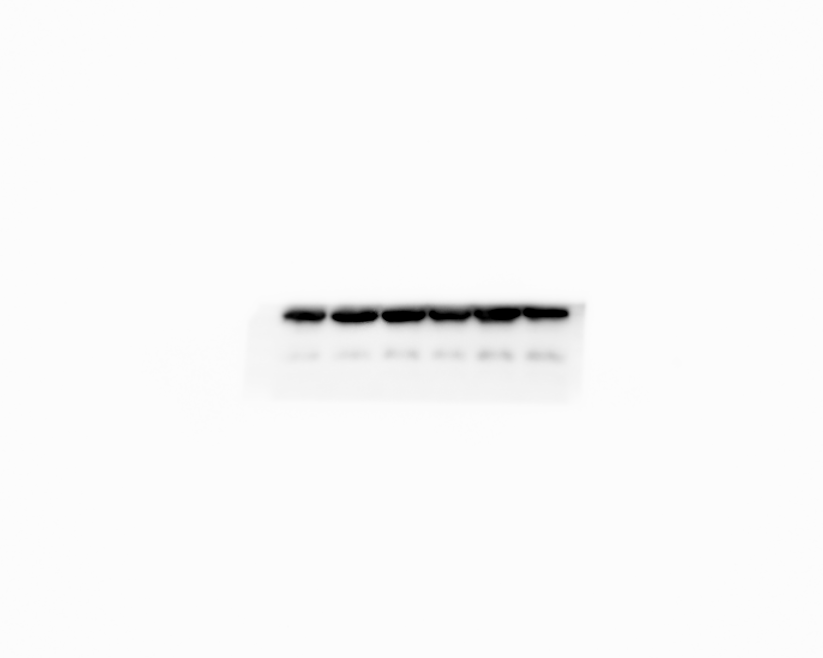


Right, Top

ANXA3 36KD,:


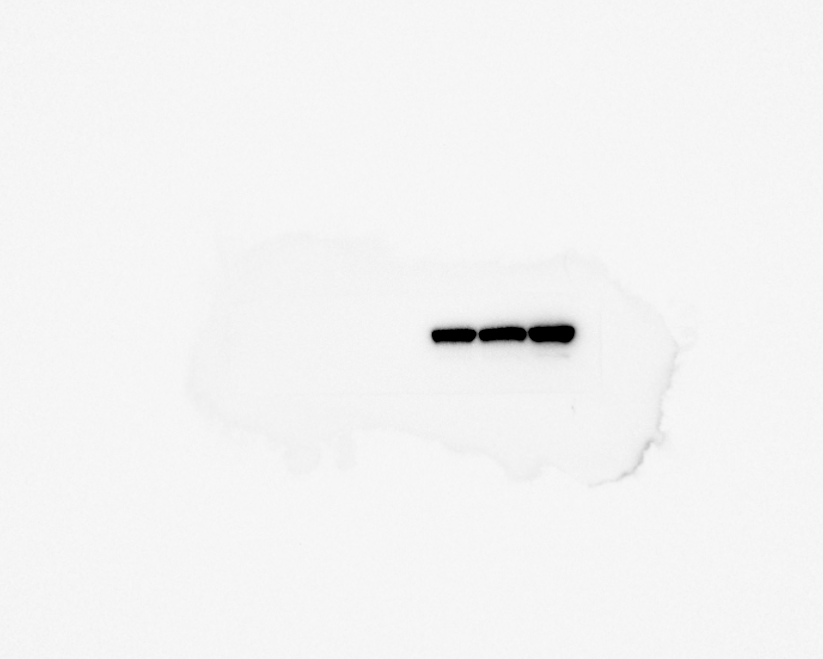


LC3B:


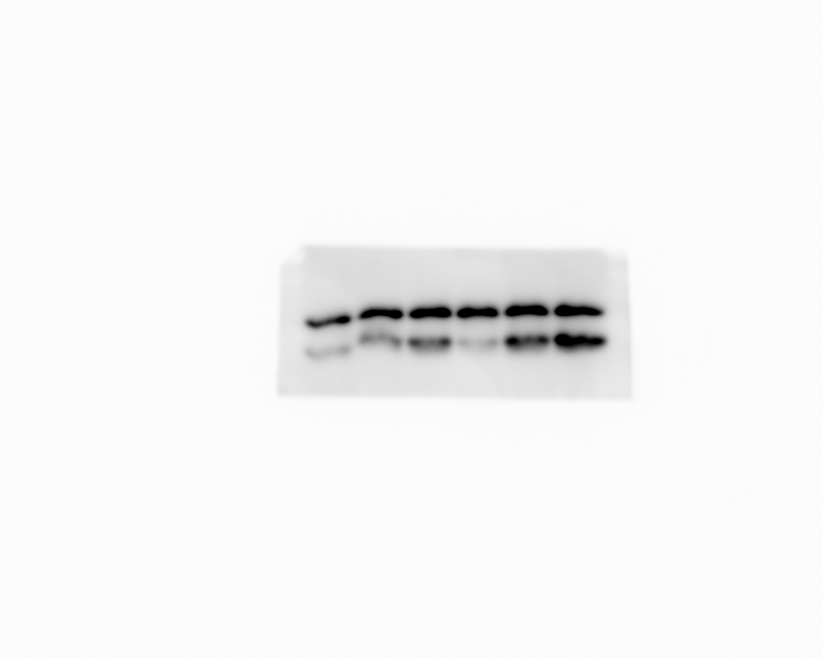


GAPDH:


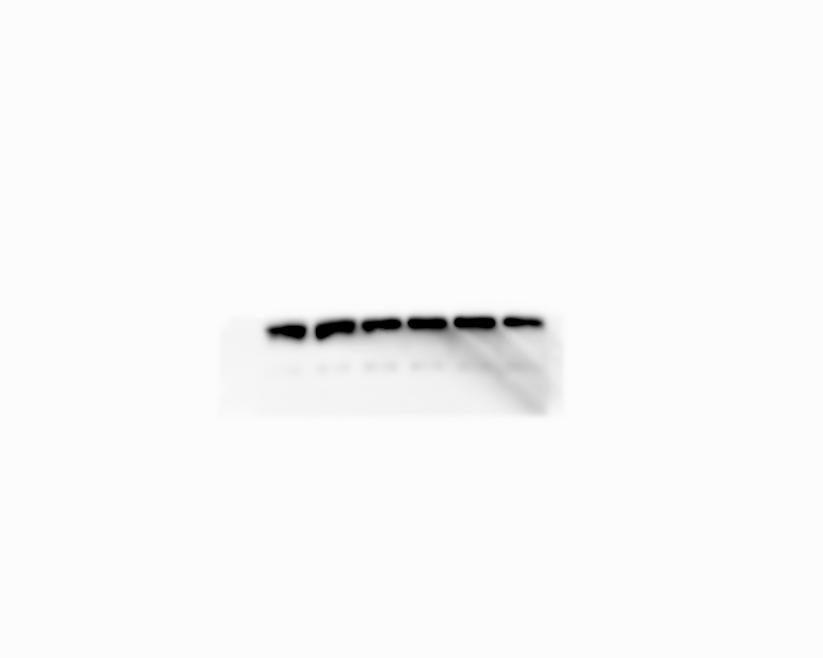


Bottom

ANXA3 36KD,


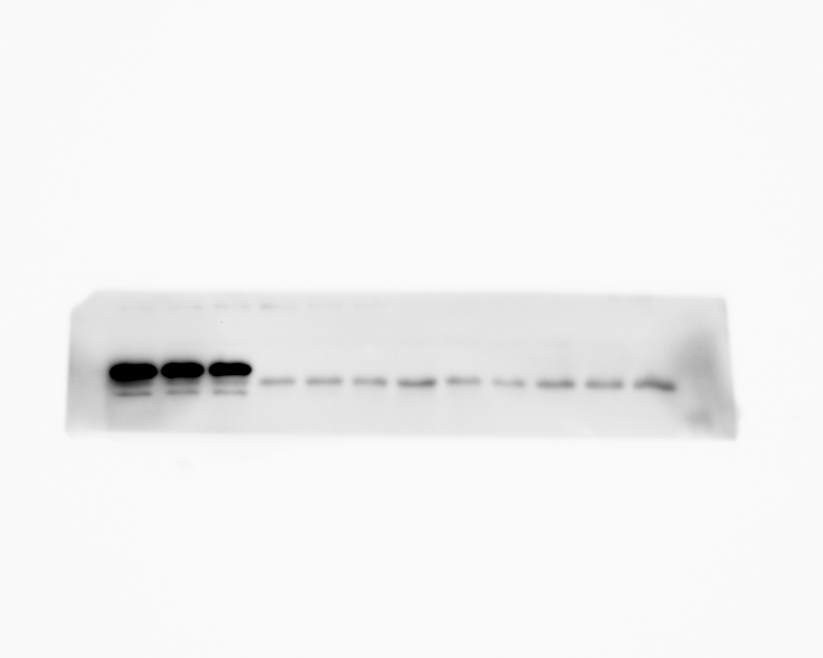


LC3B：


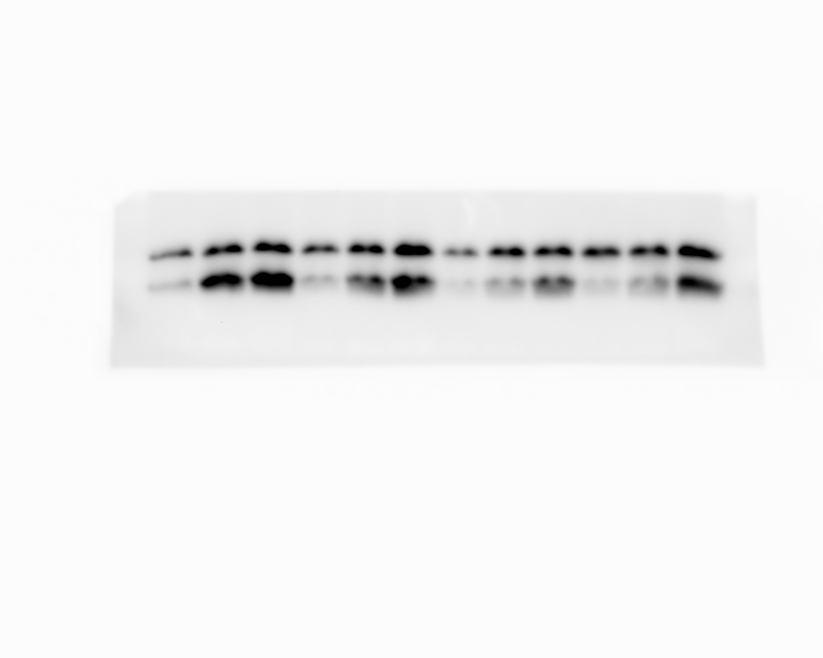


GAPDH:


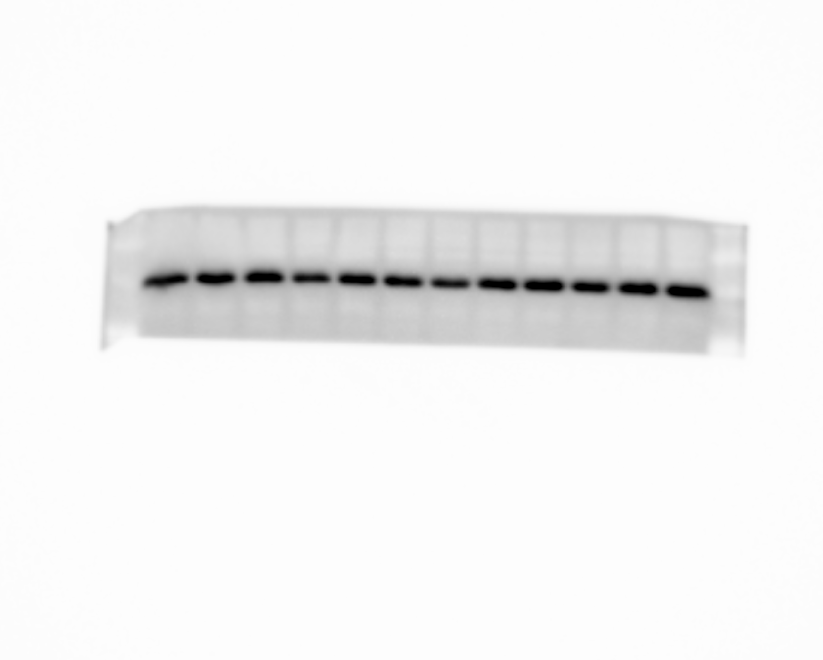


Fig3J:

ANXA3：


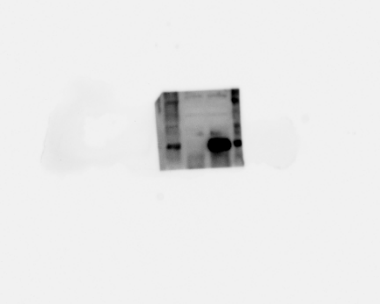

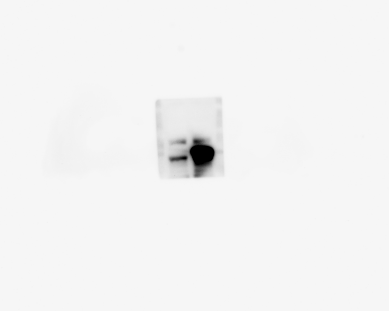

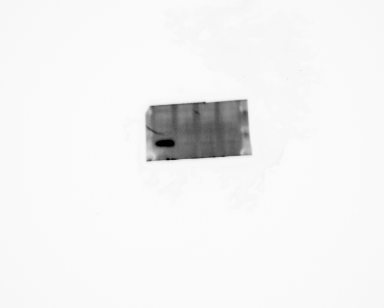


E-cadherin:


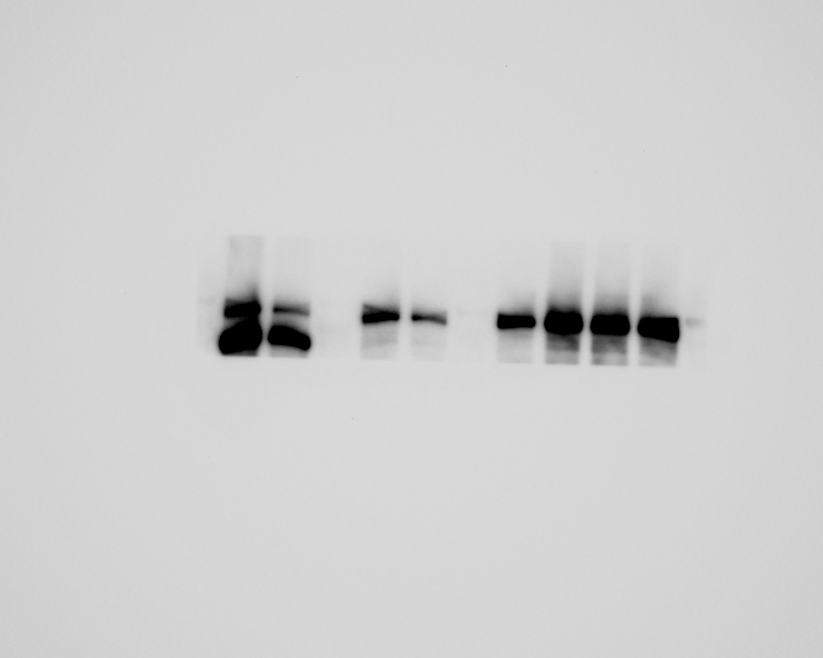


claudin-1：


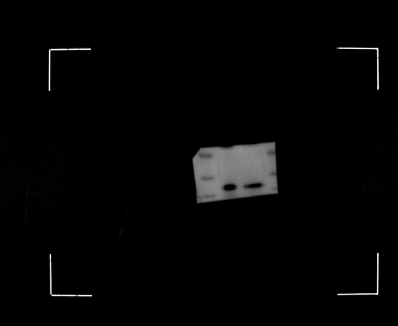

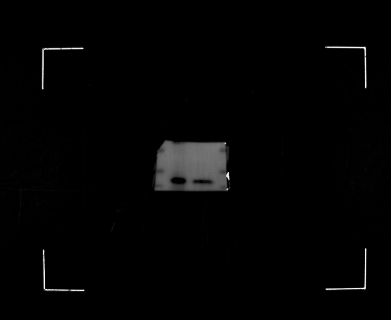

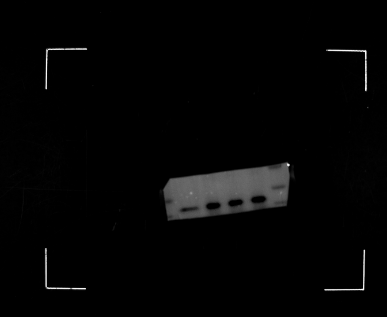


ZO-1：


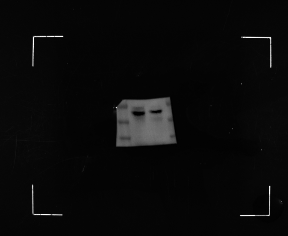

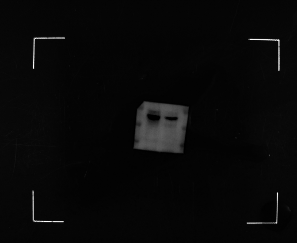

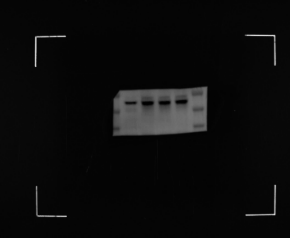


N-cadherin:


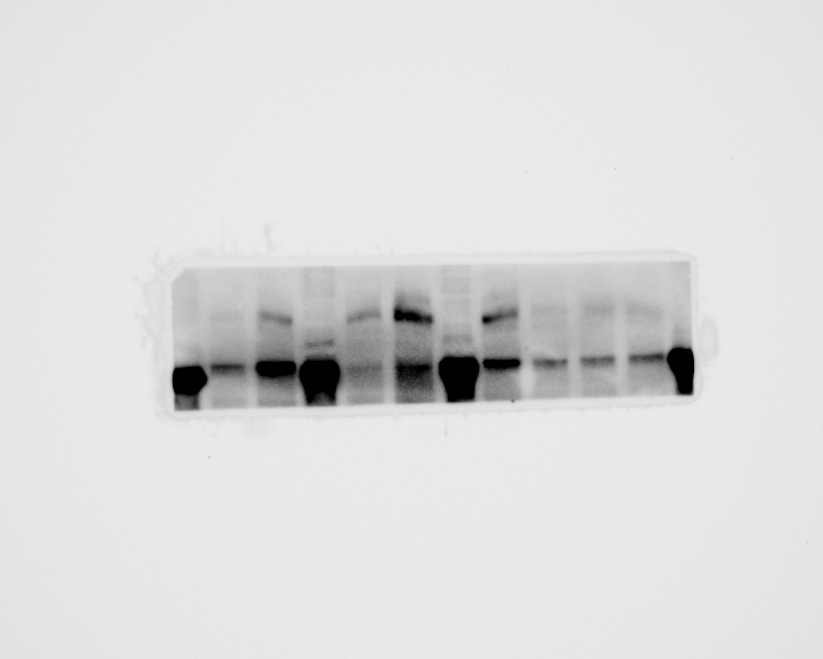


Vimentin:


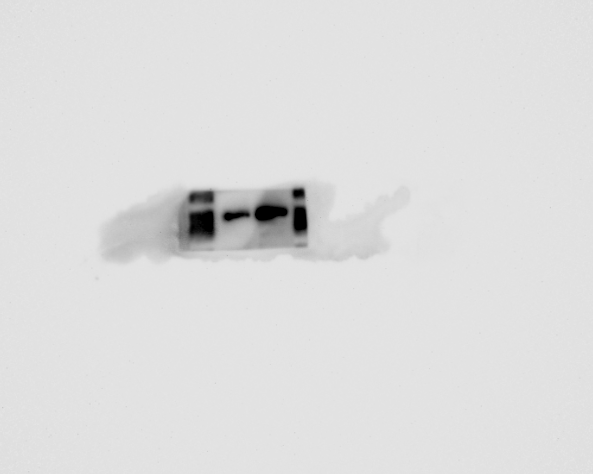

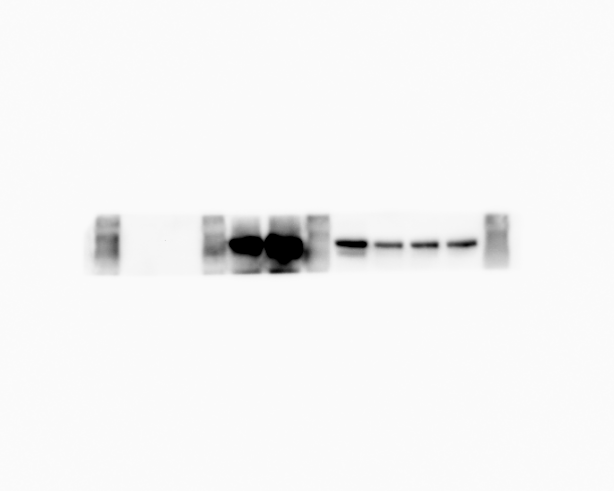


Slug:


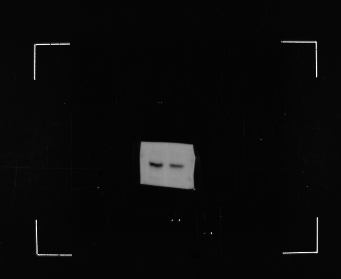

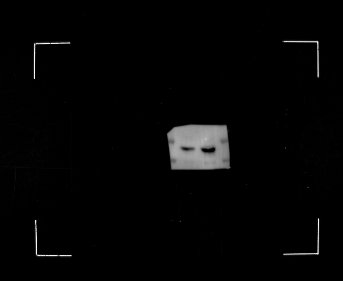

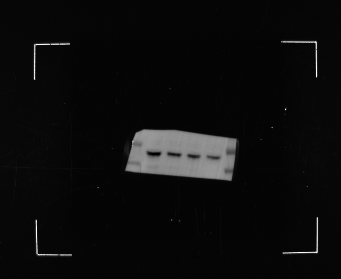


Snail:


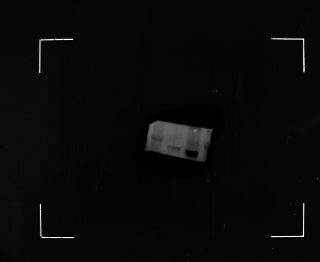

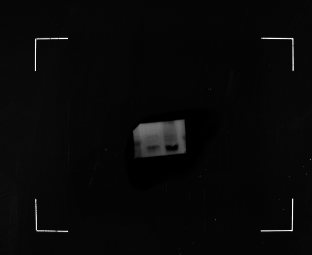

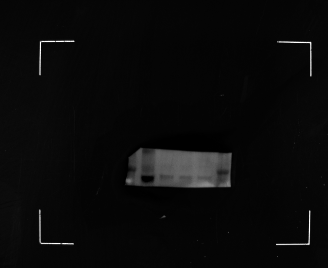


ZEB1:


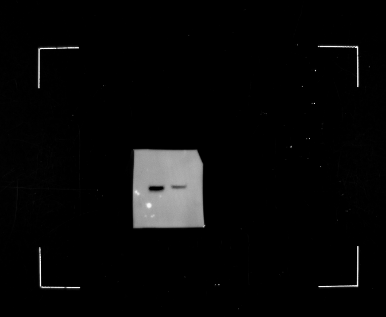

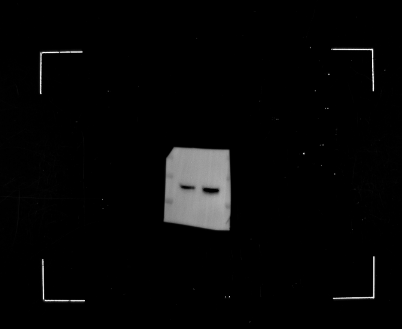

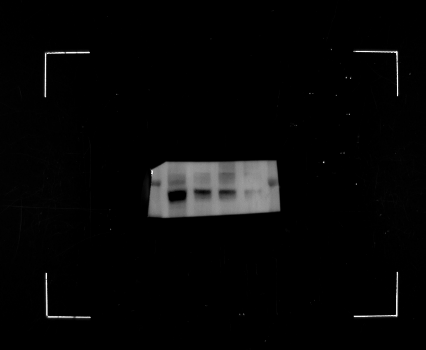


Fibronectin:


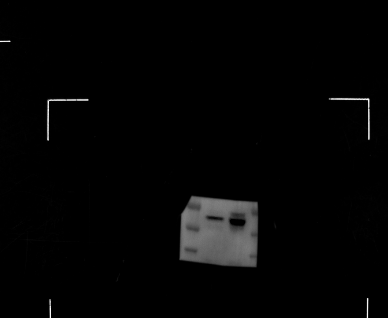

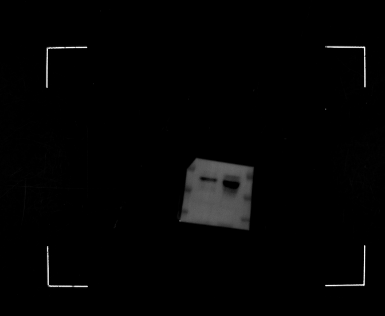

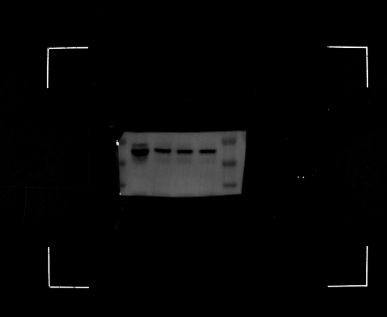


GAPDH:


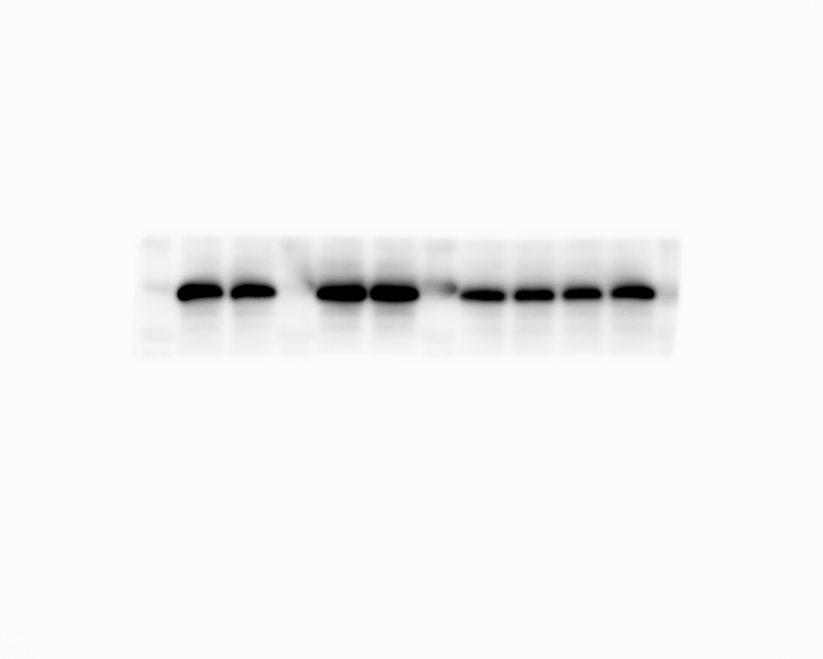


Fig 4. F

Left：

ANXA3：


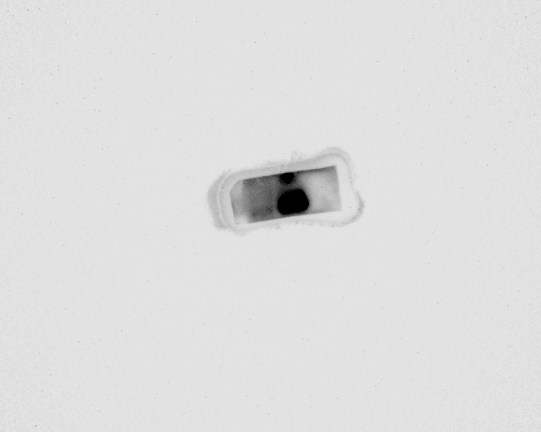

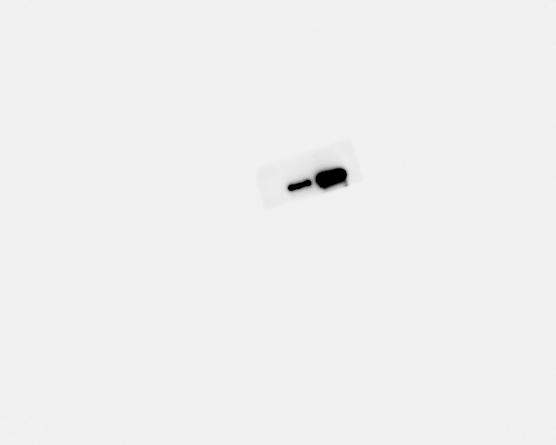


PDGFA：


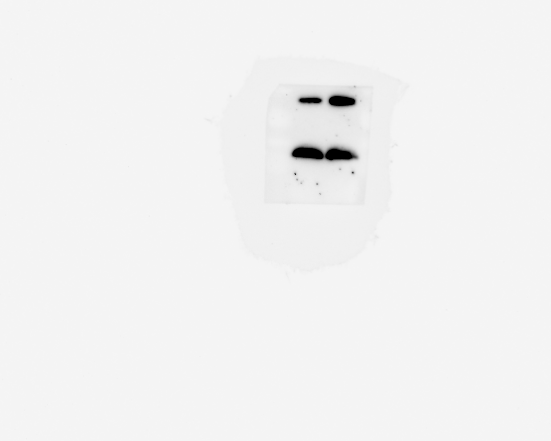

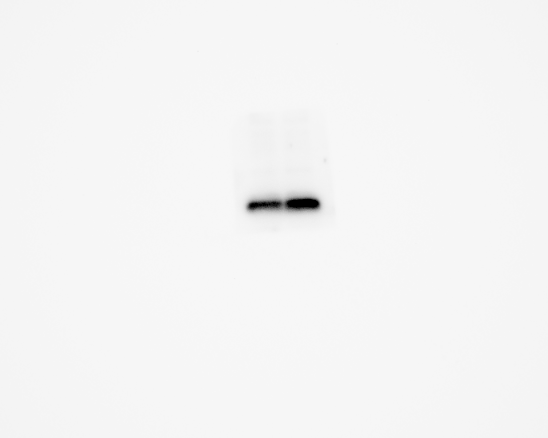


β-Actin:


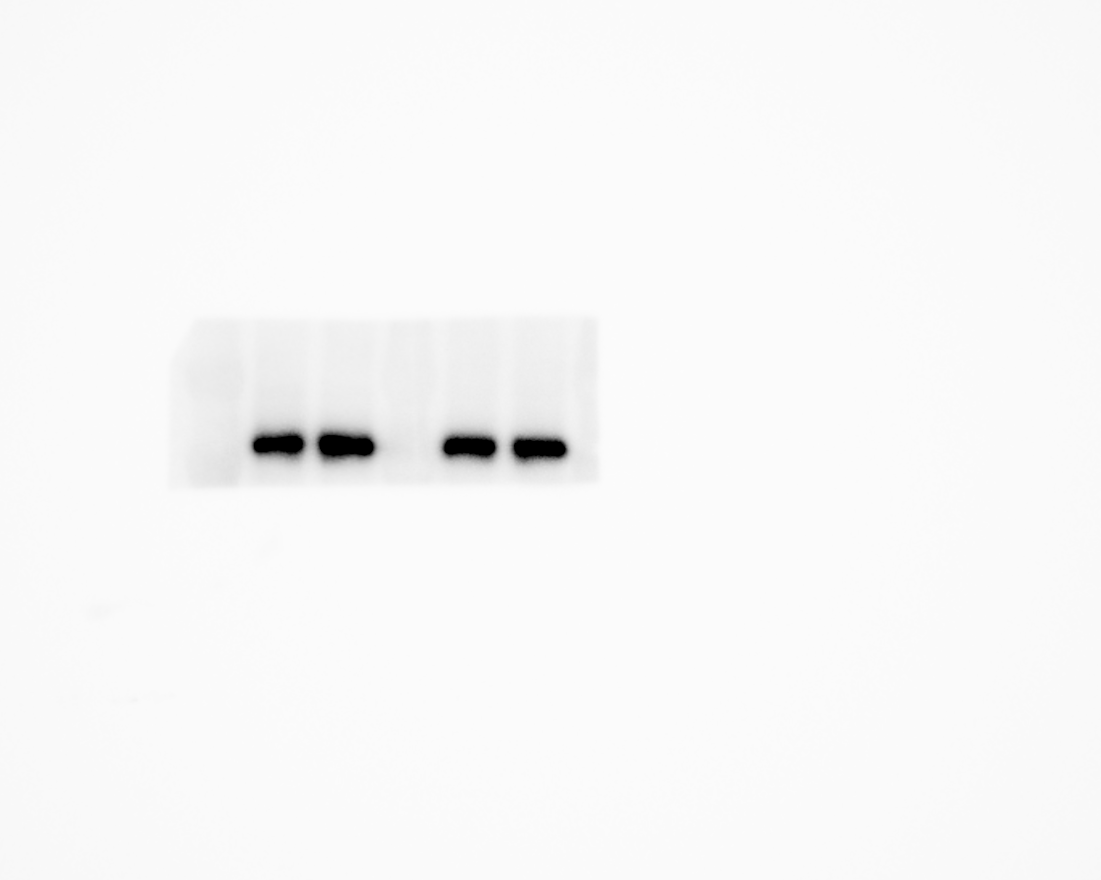


Right：

ANXA3：


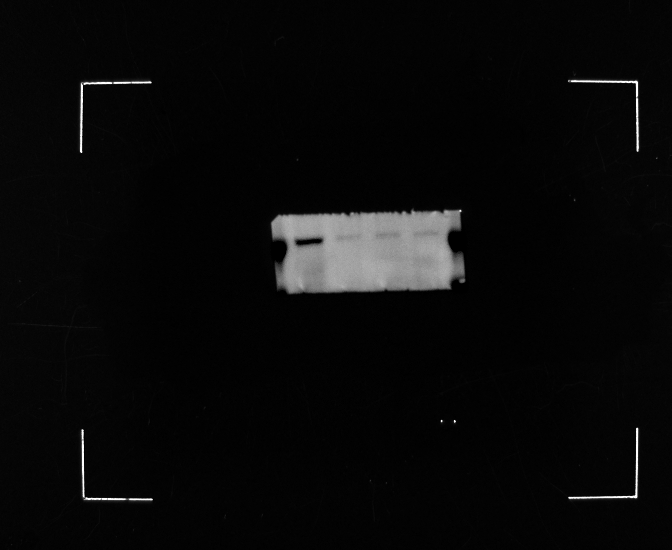


PDGFA（Right）：


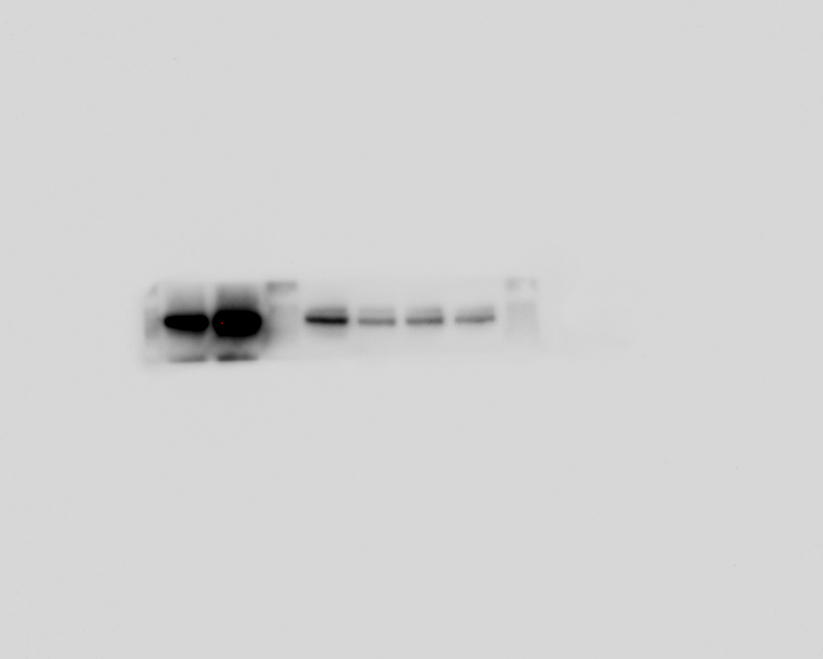


β-Actin:


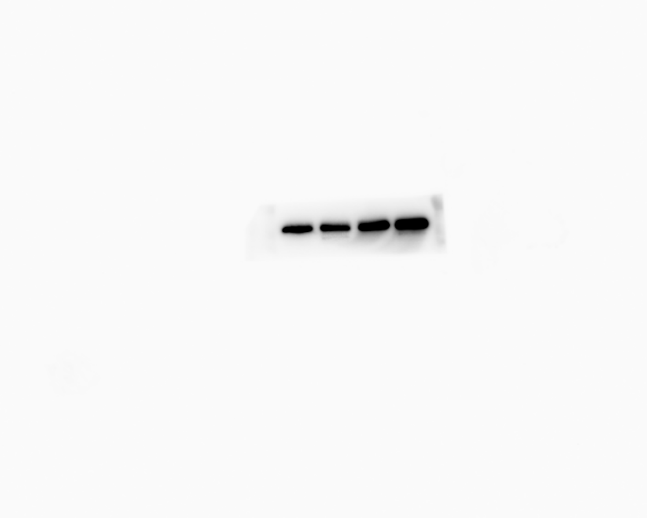


Fig5. B

ANXA3：


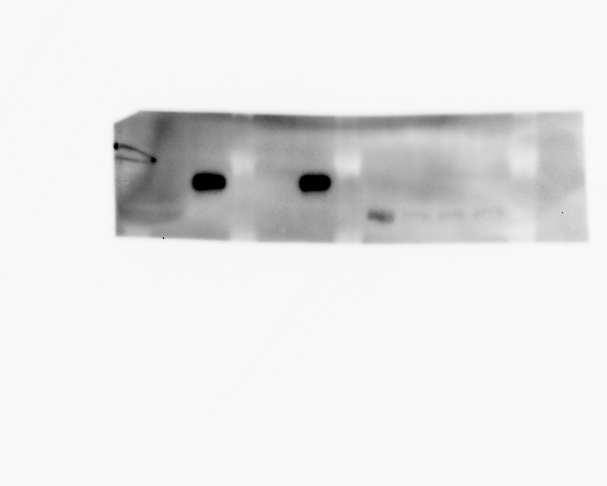

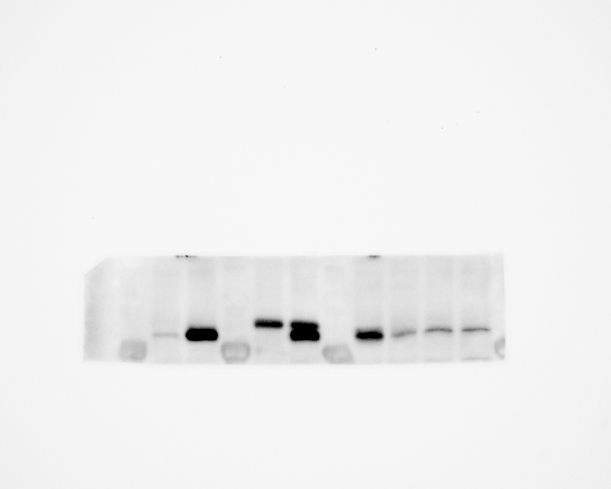


p-PI3K：


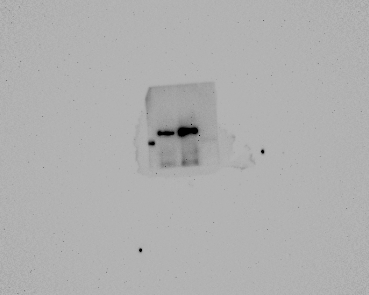

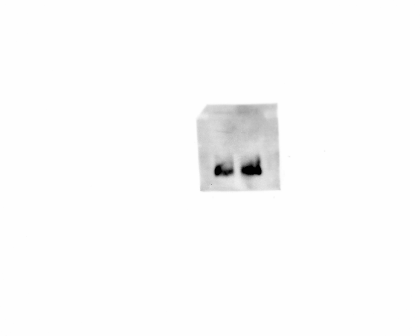

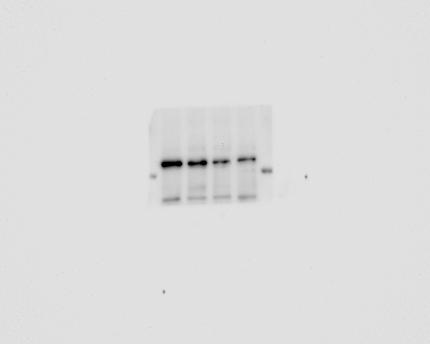


PI3K：


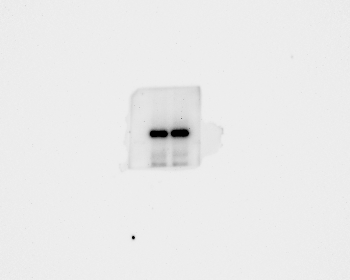

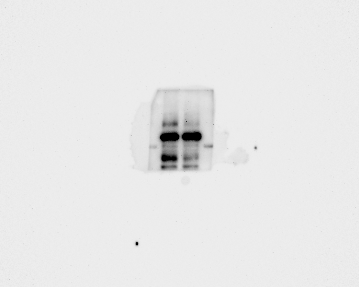

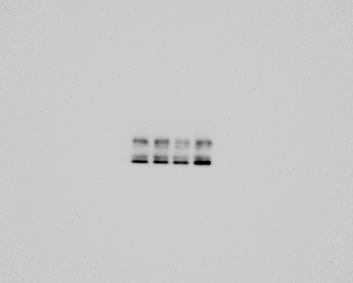


p-AKT:


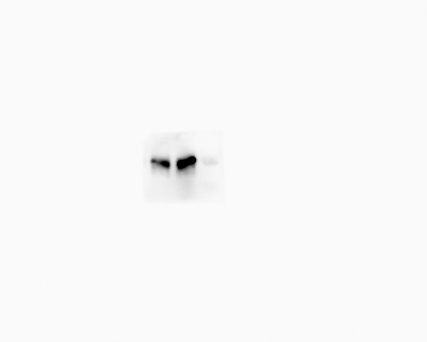

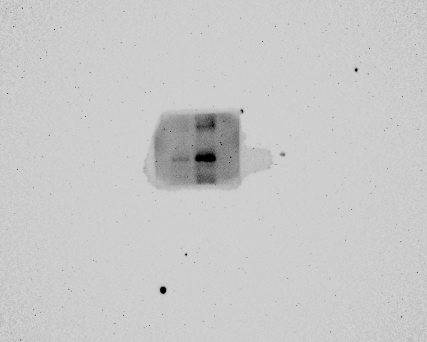

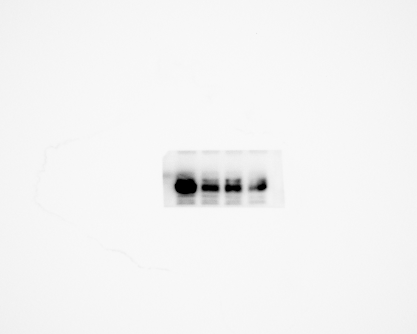


AKT:


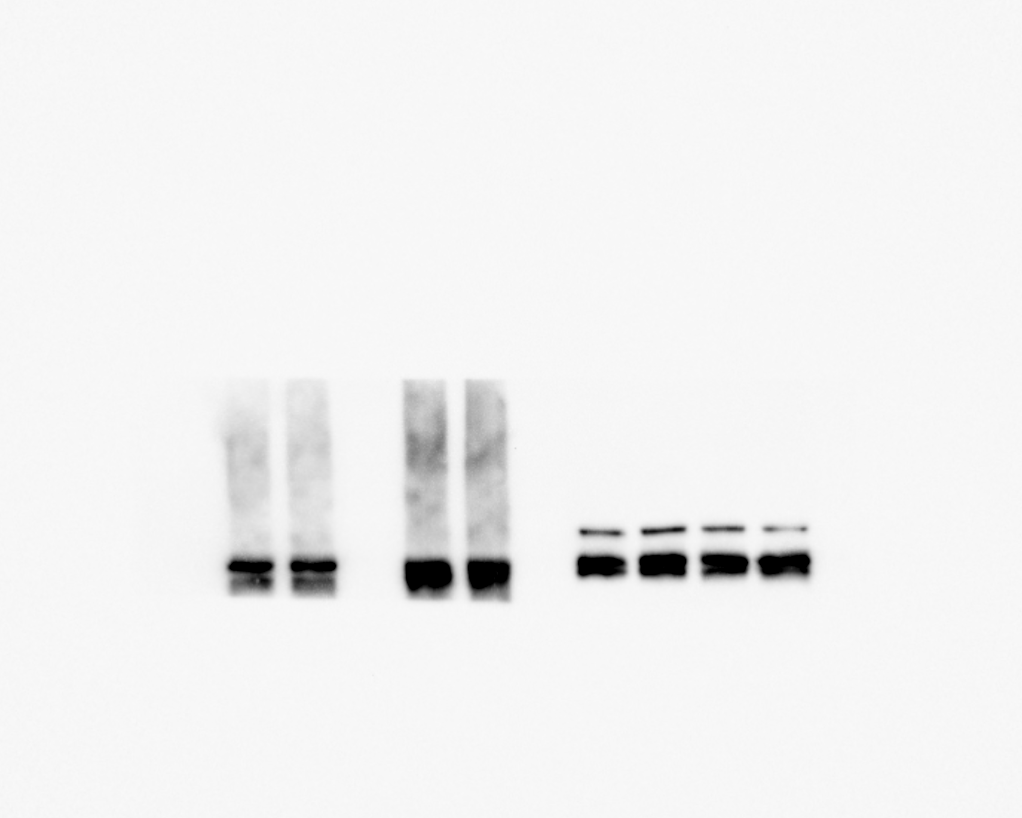


GAPDH:


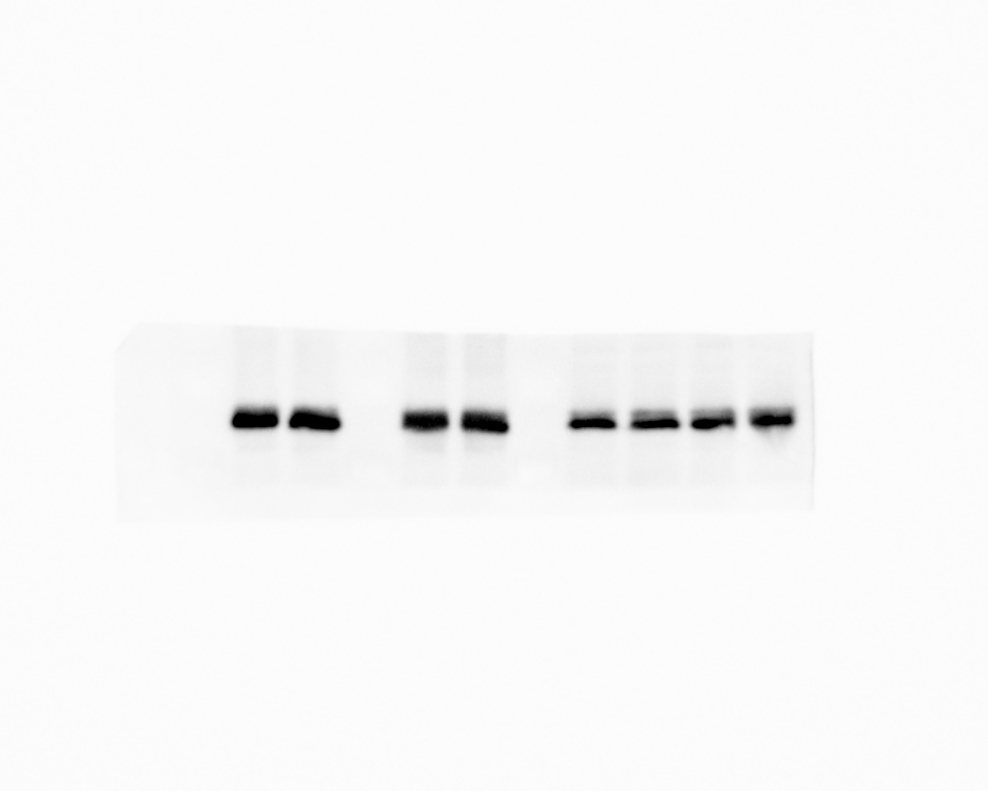


Fig5.D

ANXA3：


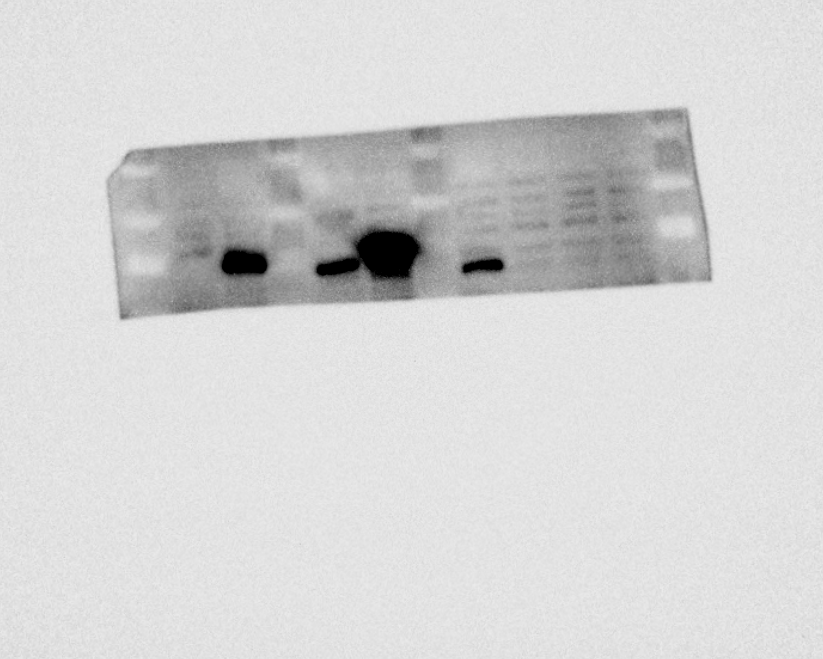


p-GSK


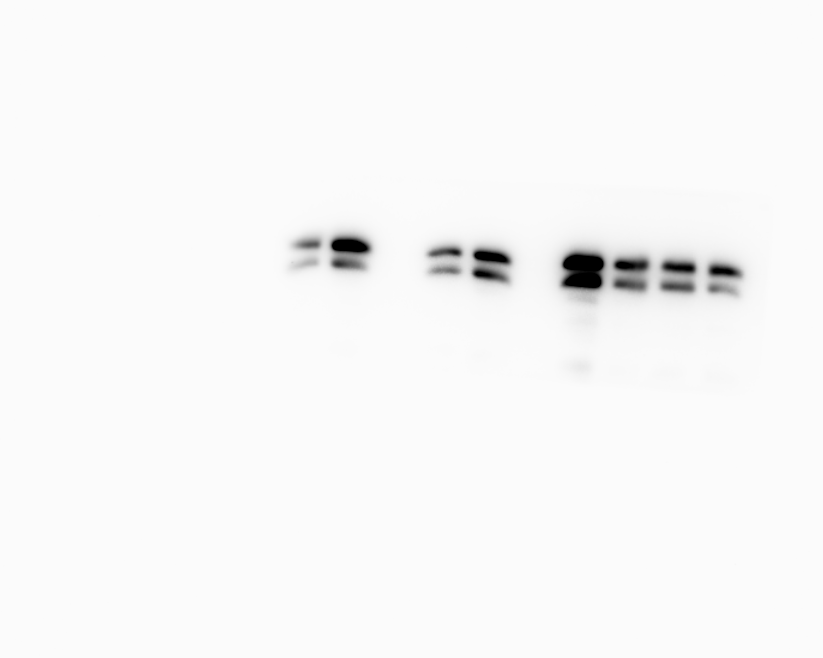


GSK:


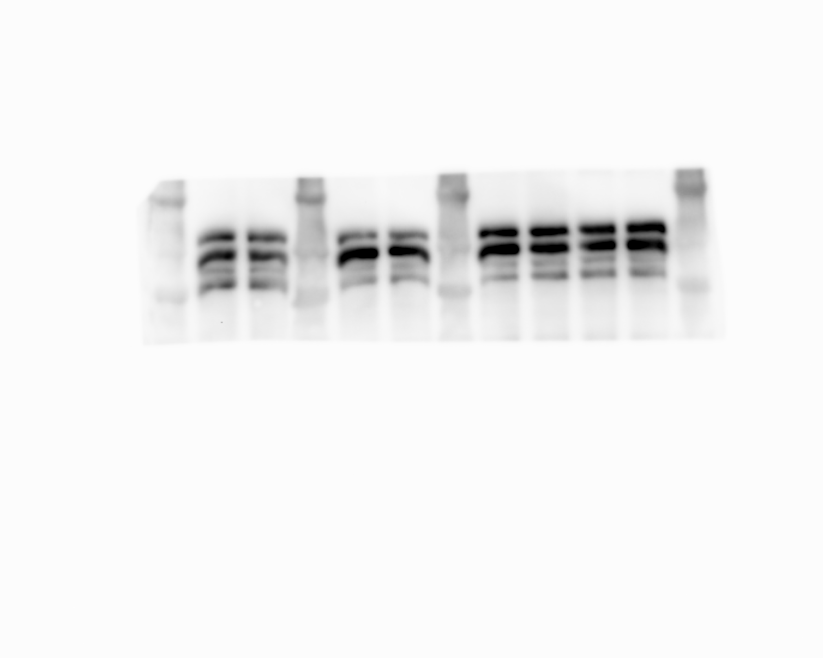


β-catenin:


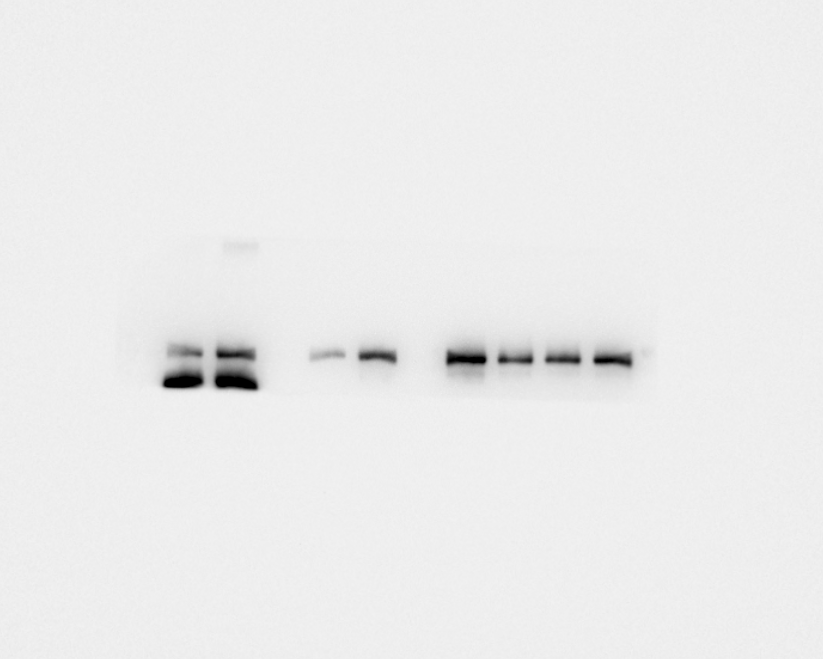


p-ERK


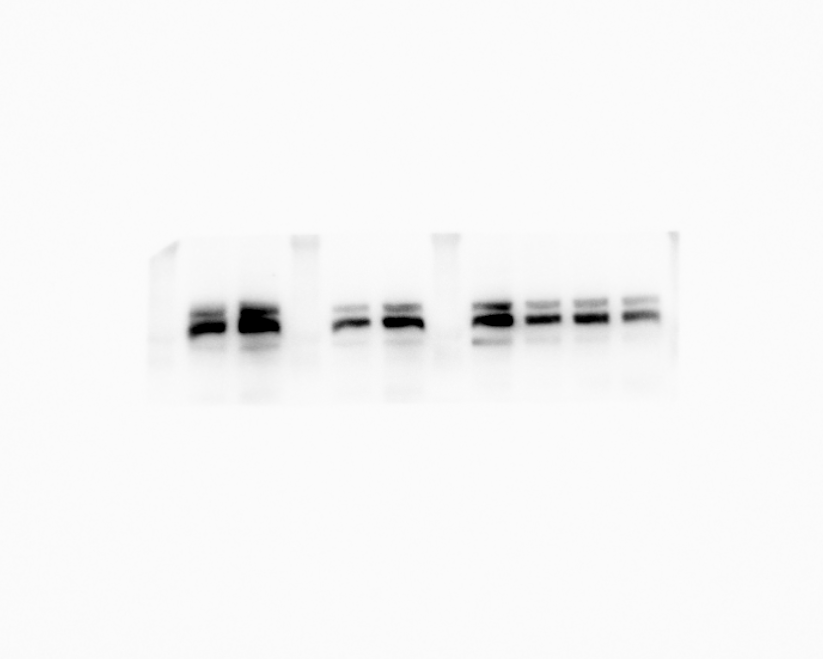


ERK


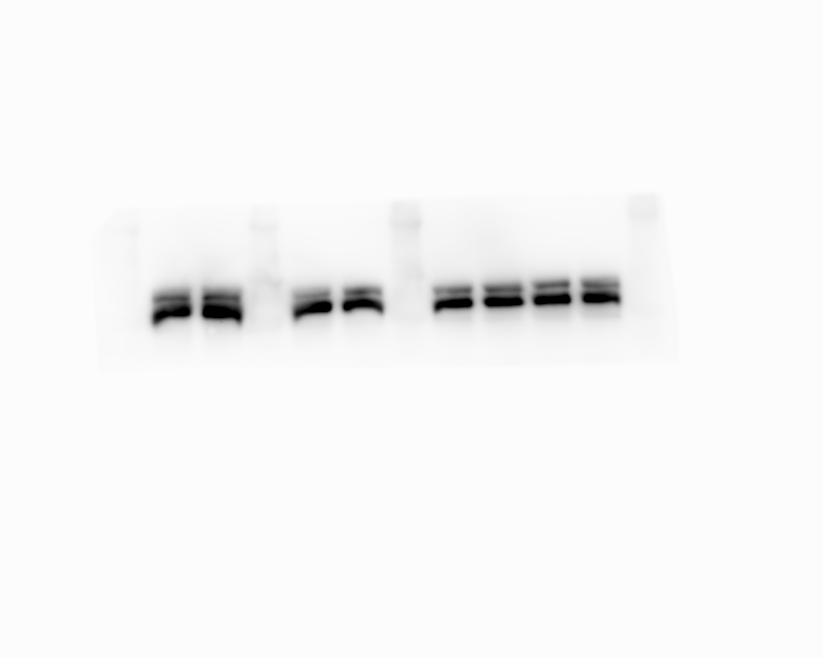


p-c-Jun


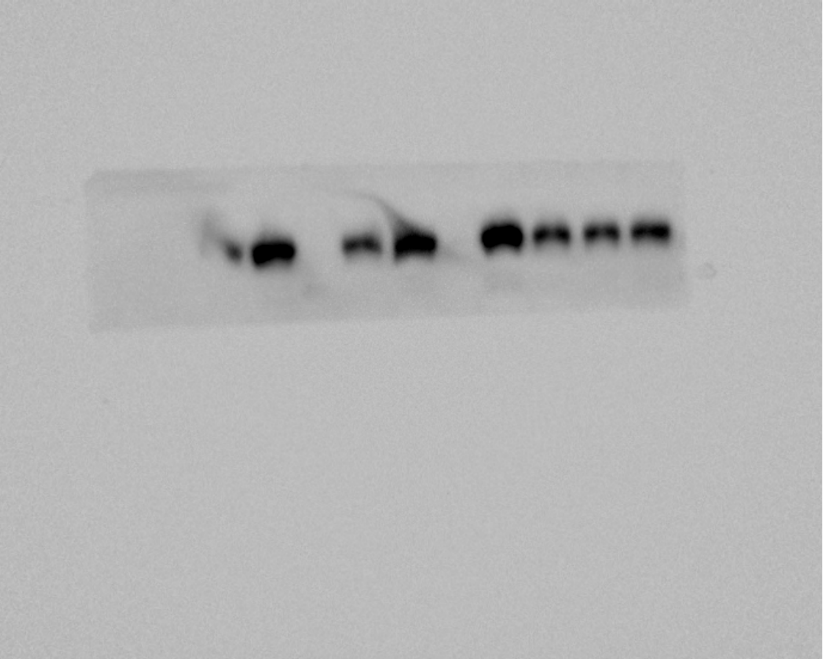


c-Jun


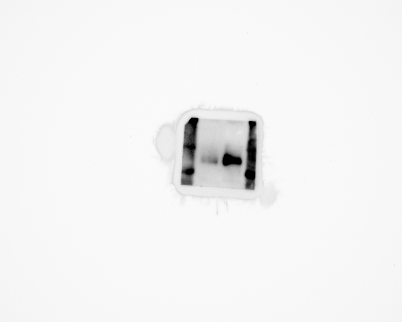

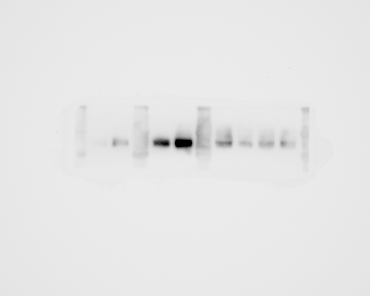

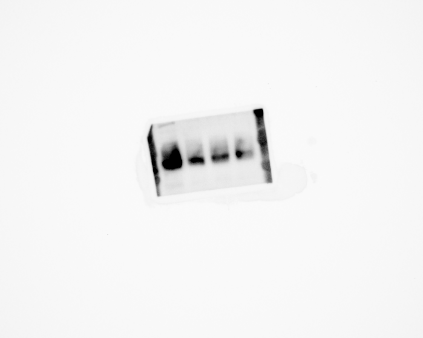


GAPDH（Bottom）:


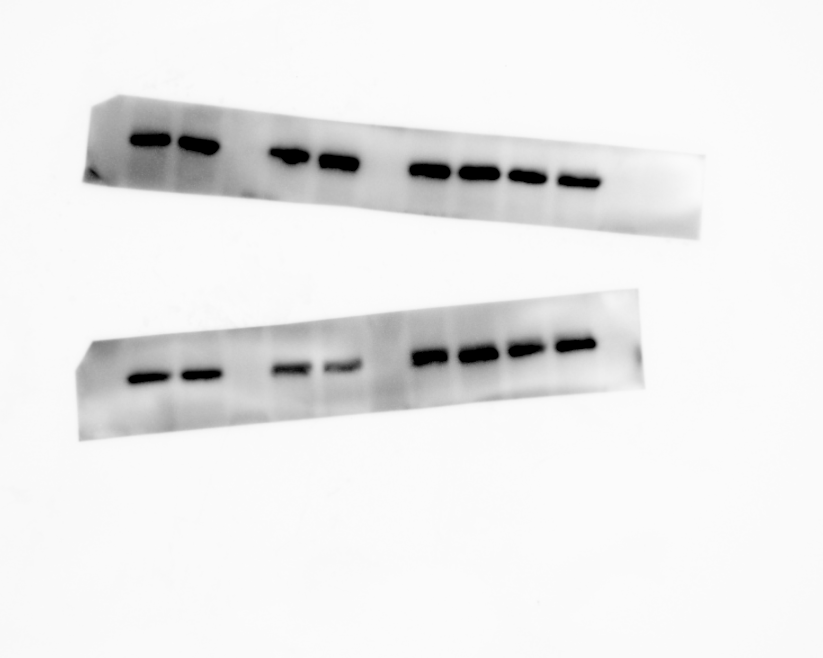
(down)

Fig5.E

ANXA3


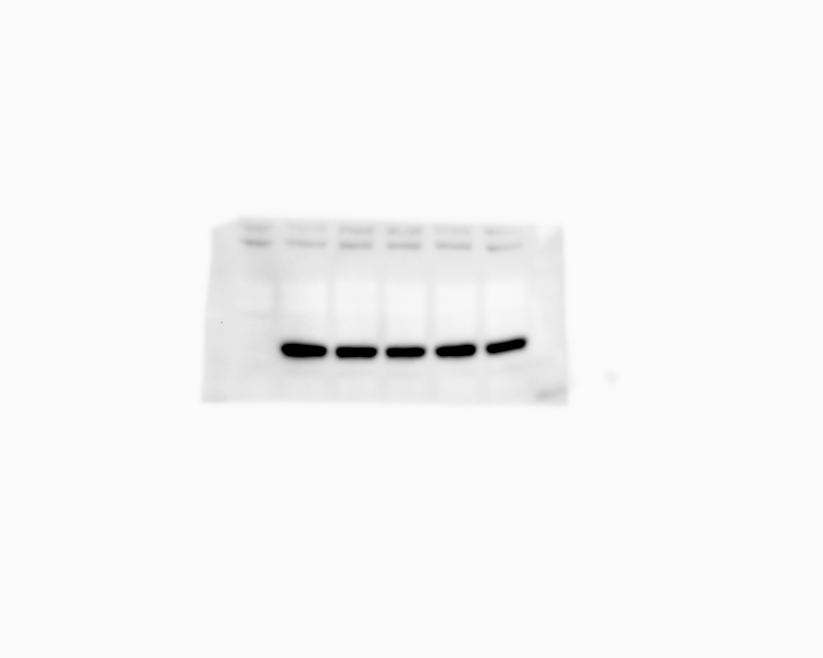


p-PI3K


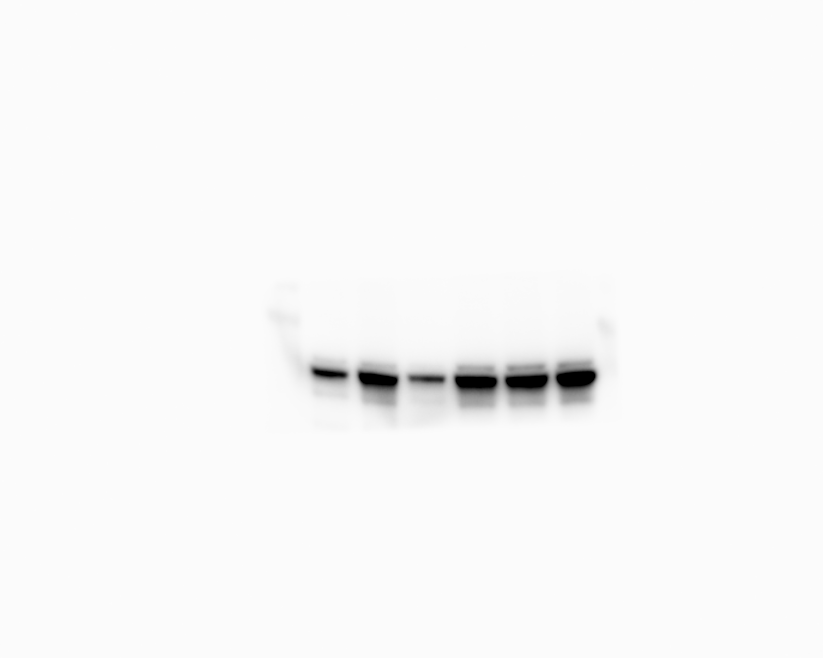


PI3K


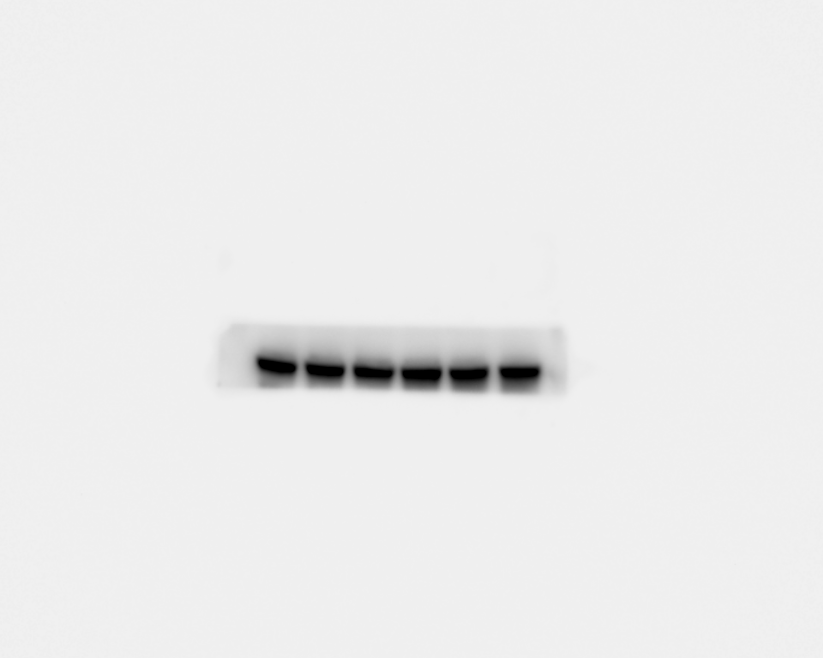


p-AKT


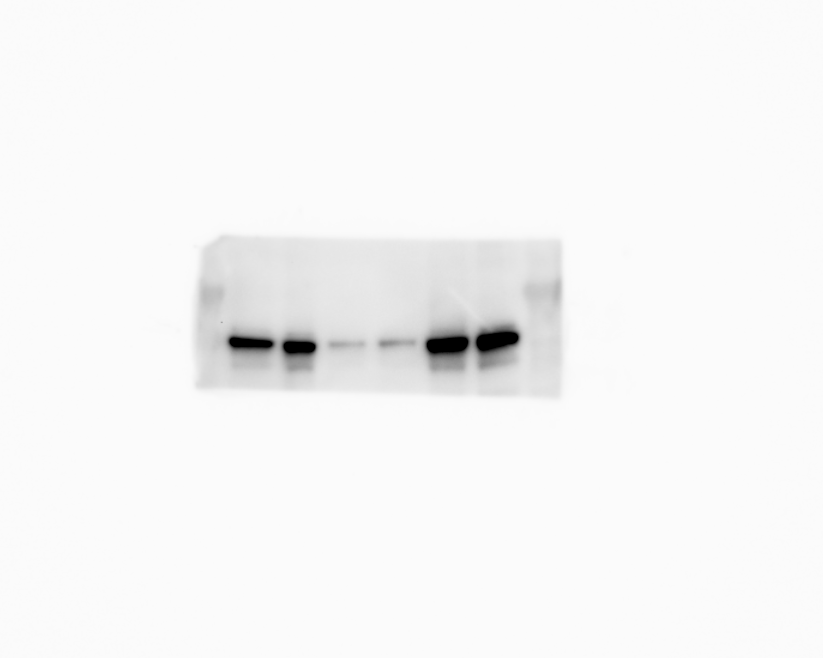


AKT


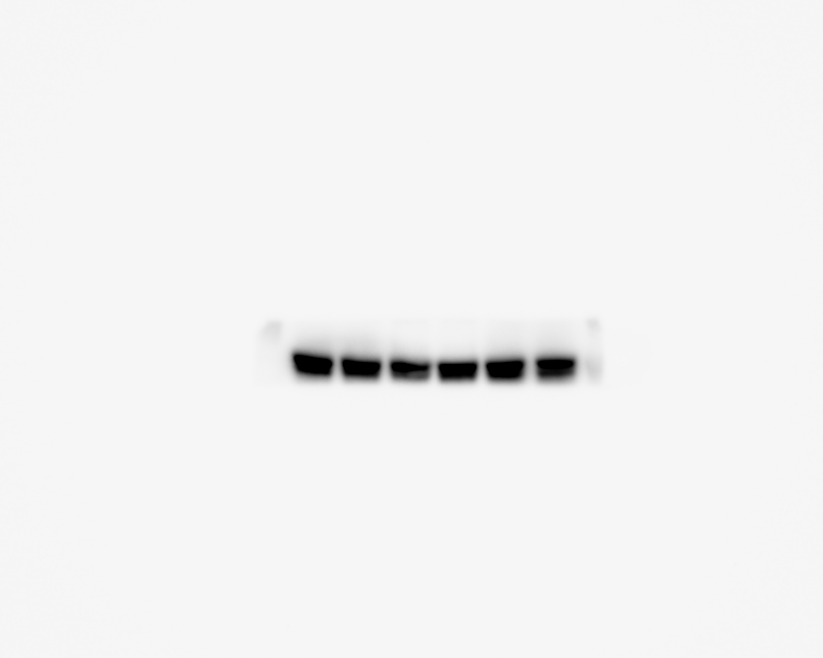


p-GSK


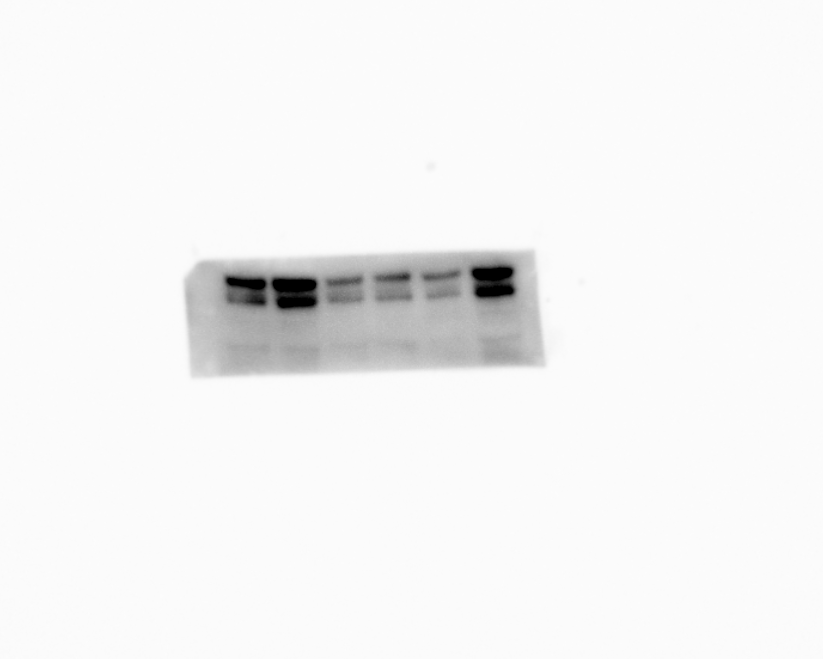


GSK


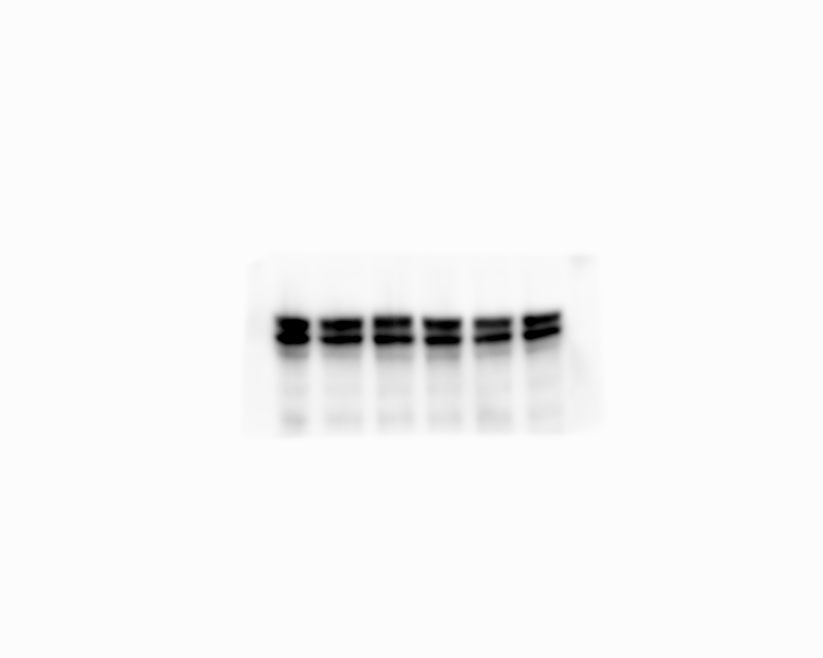


p-ERK


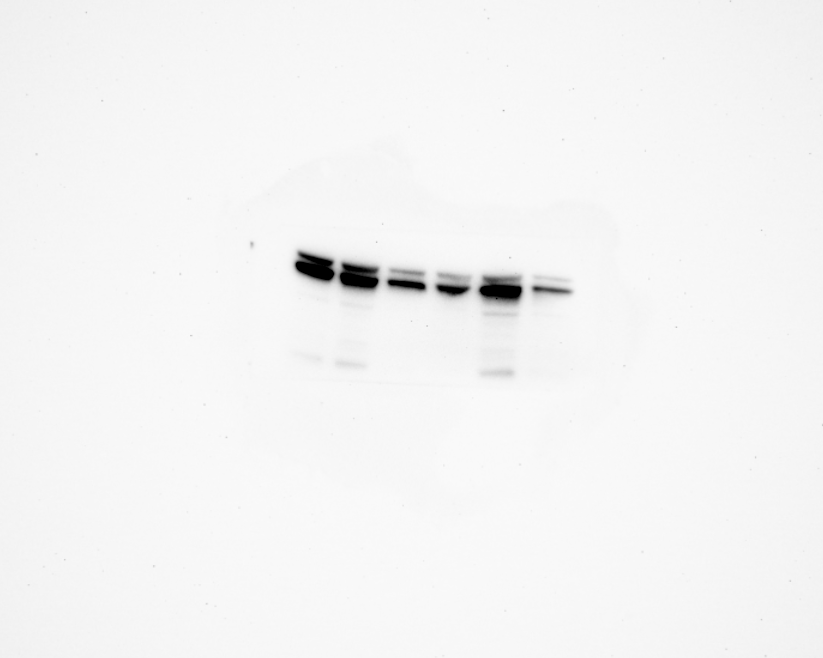


ERK


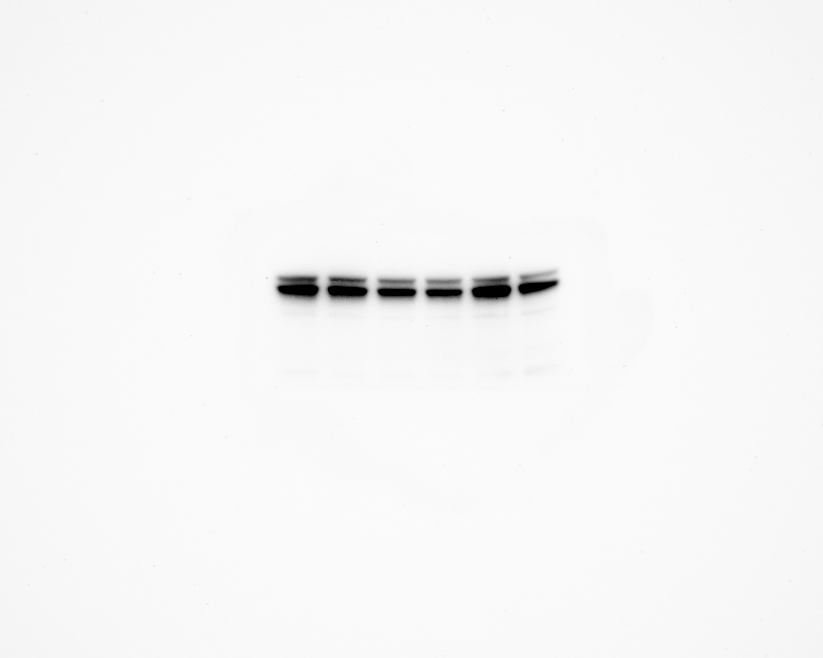


p-c-Jun


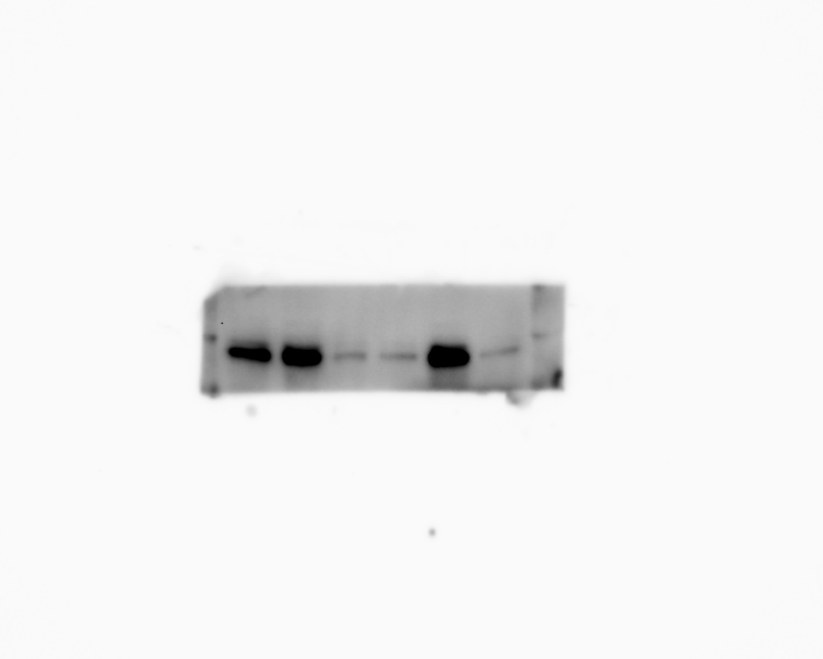


c-Jun


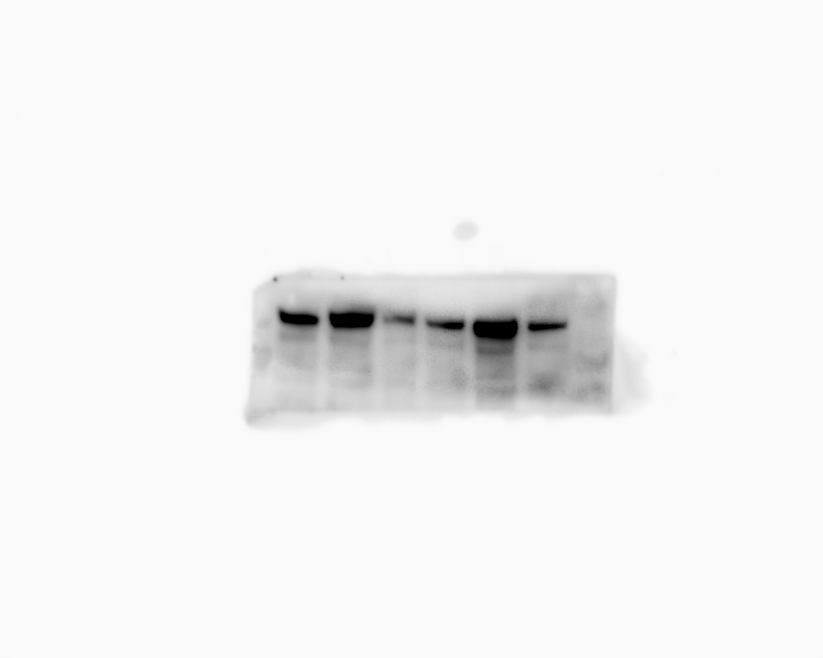


GAPDH


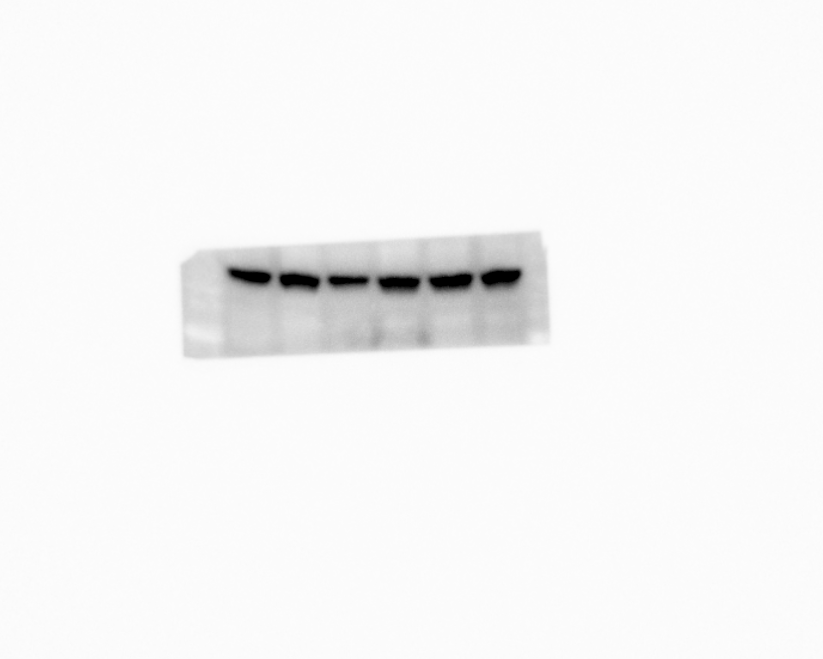


Fig5.F

Whole cell:

β-catenin


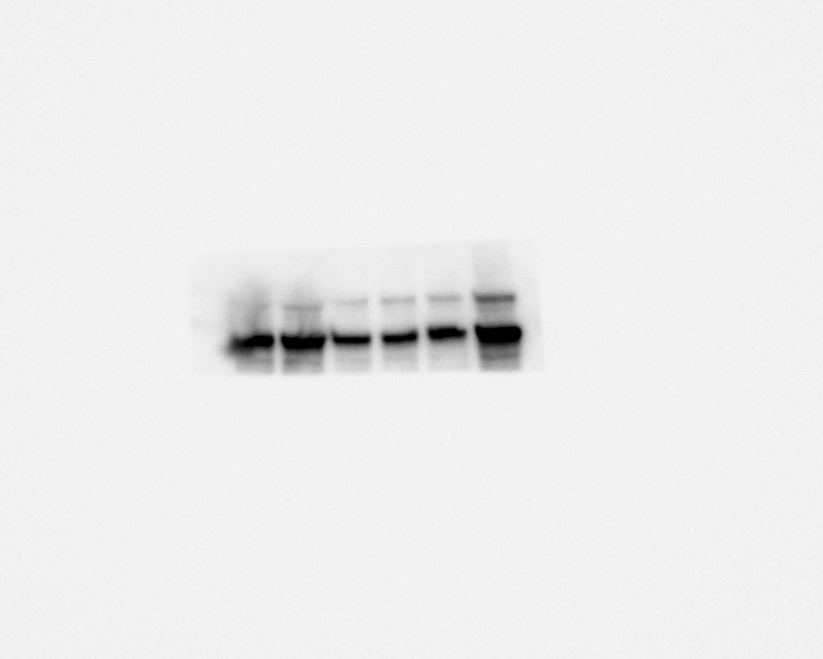


GAPDH


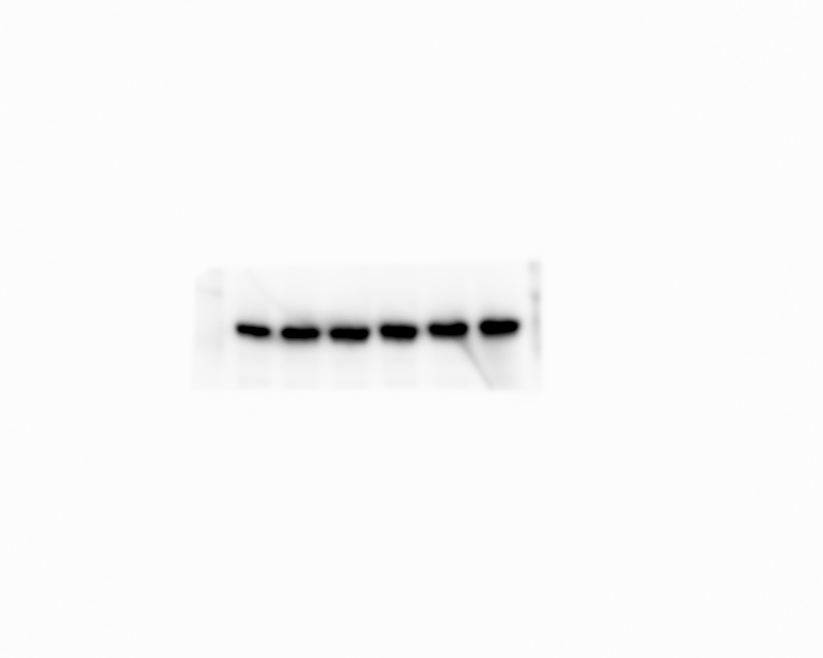


Cytoplasm:

β-catenin


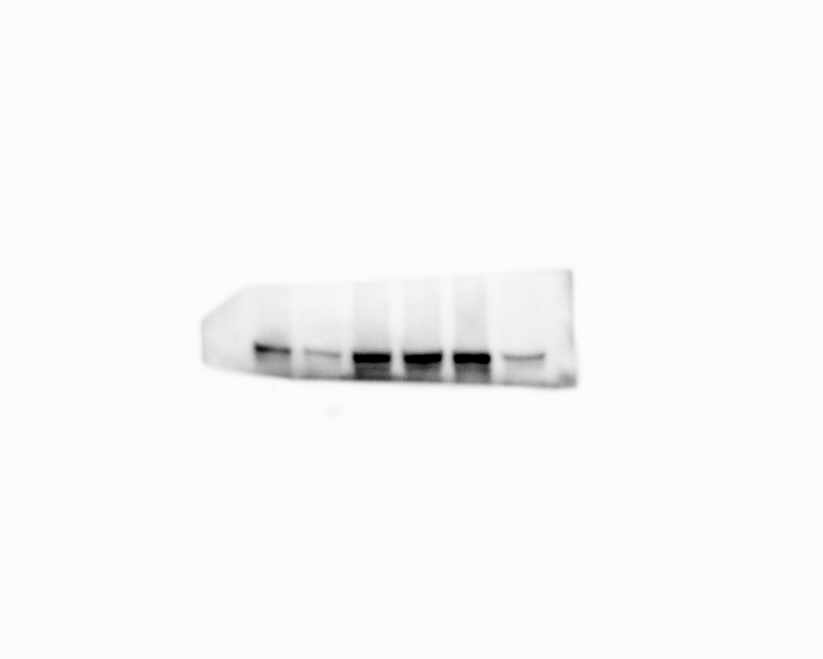


GAPDH


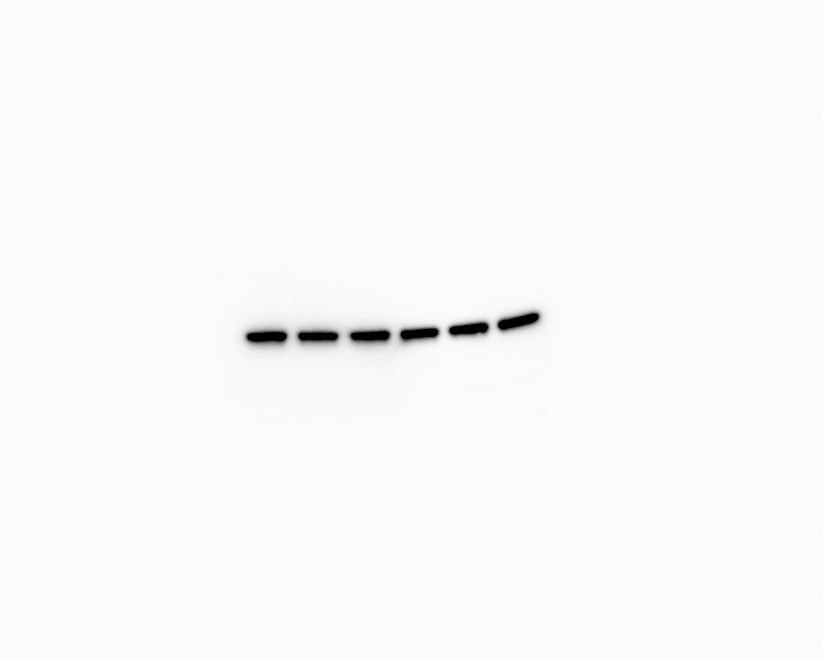


TBP


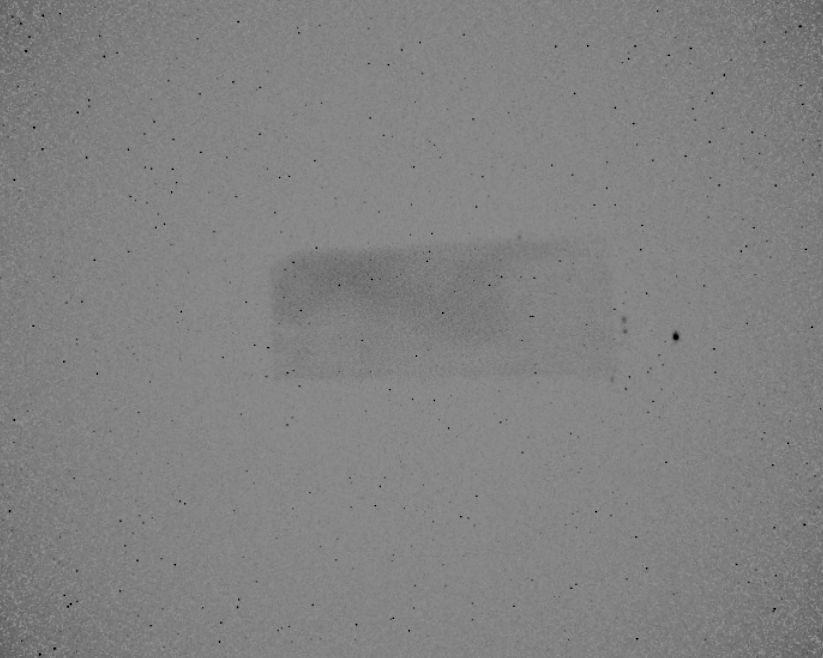


Nucleus:

β-catenin


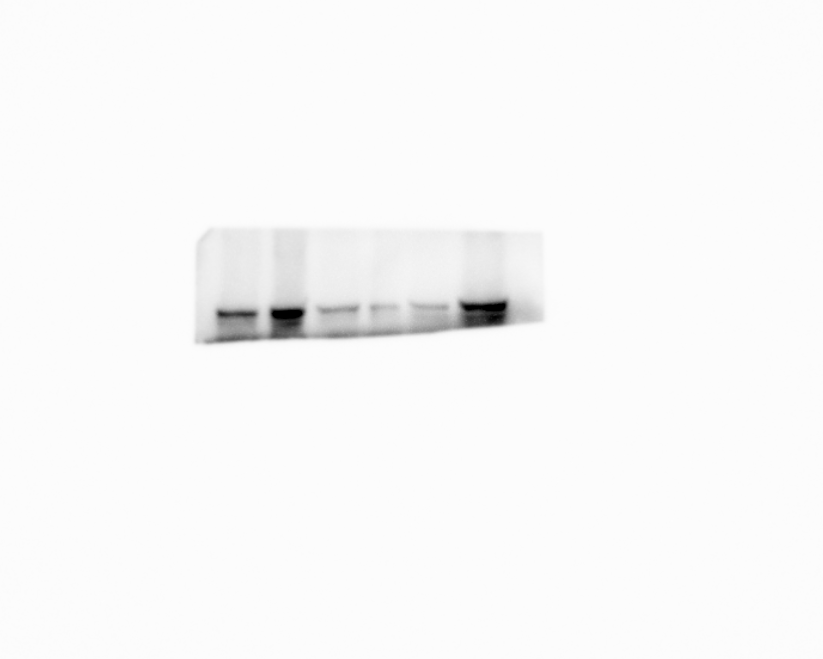


GAPDH


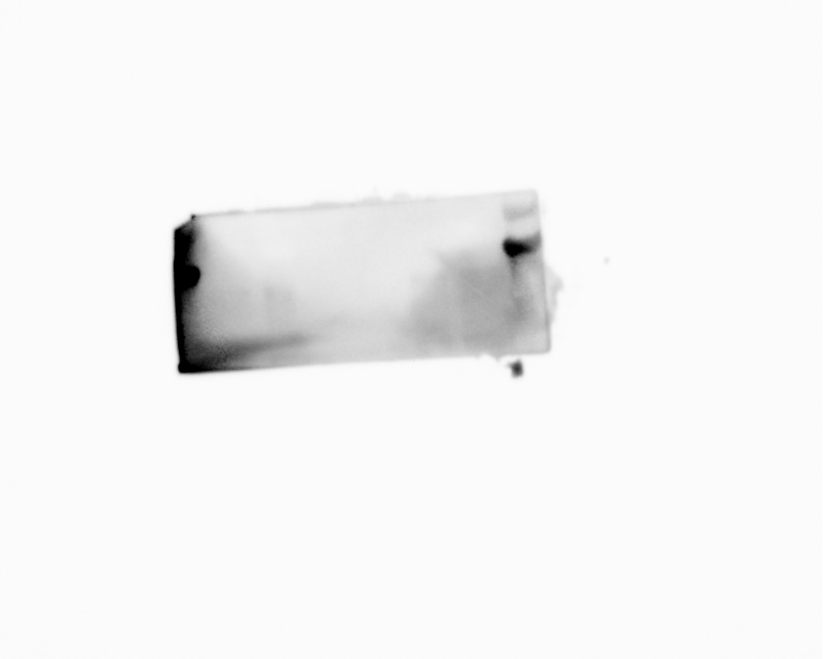


TBP


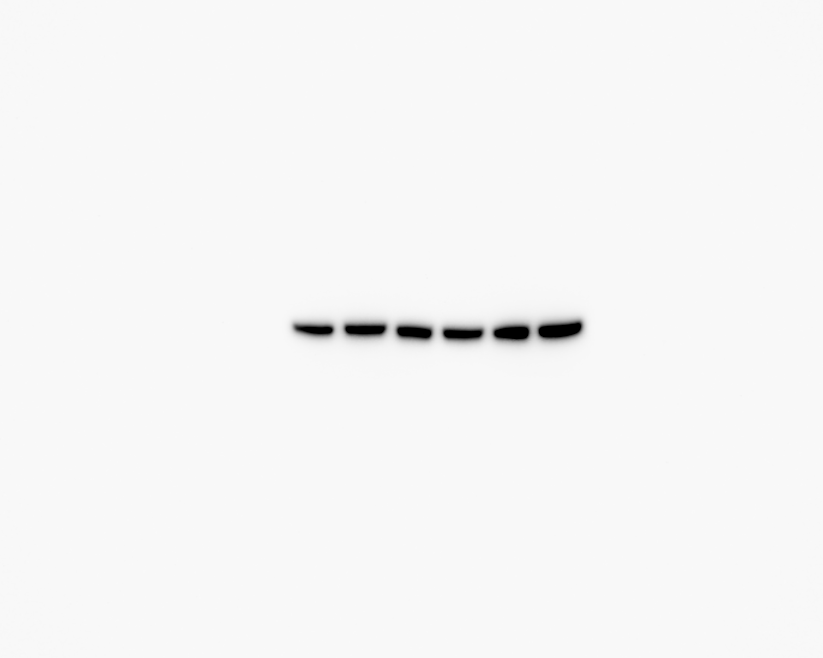


Fig6 A

p-PDGFRa


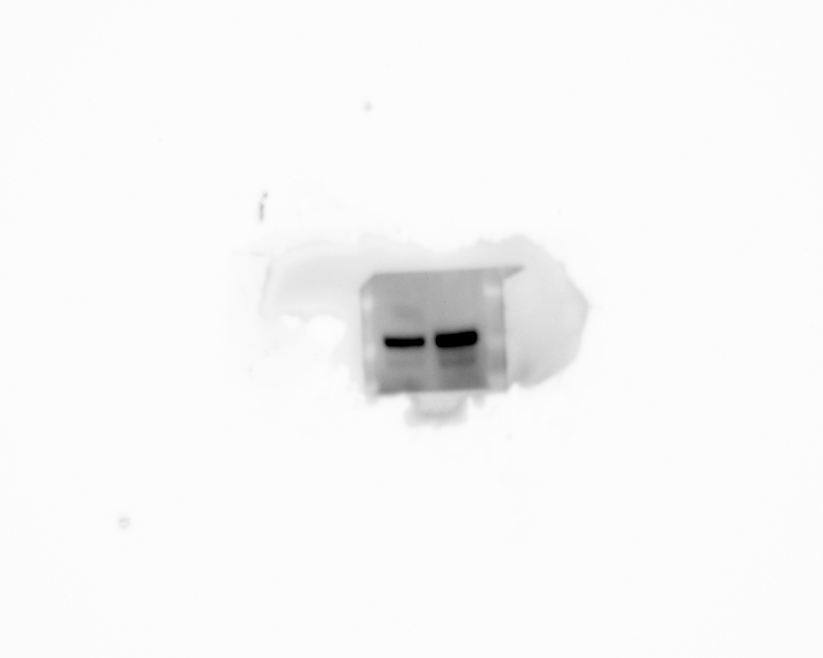


p-PI3K


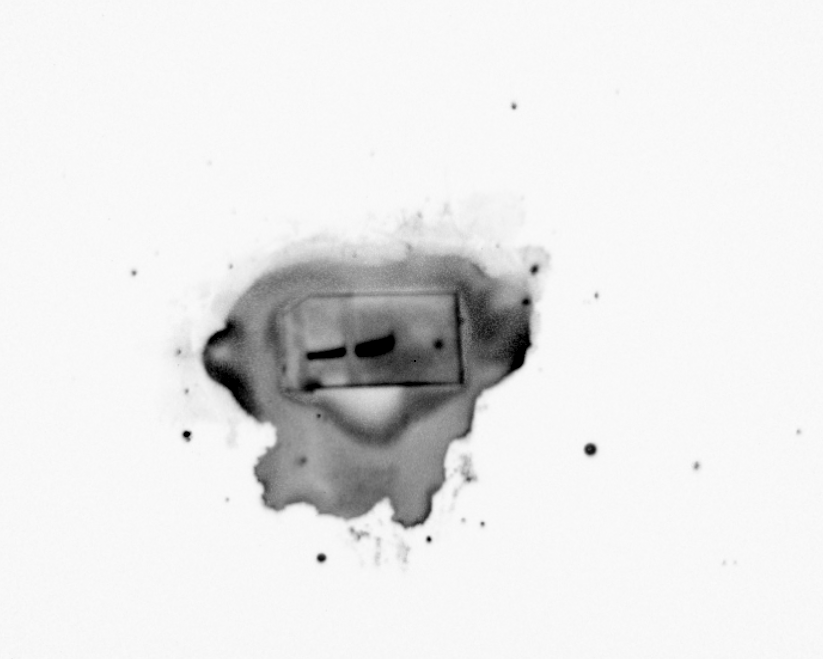


PI3K


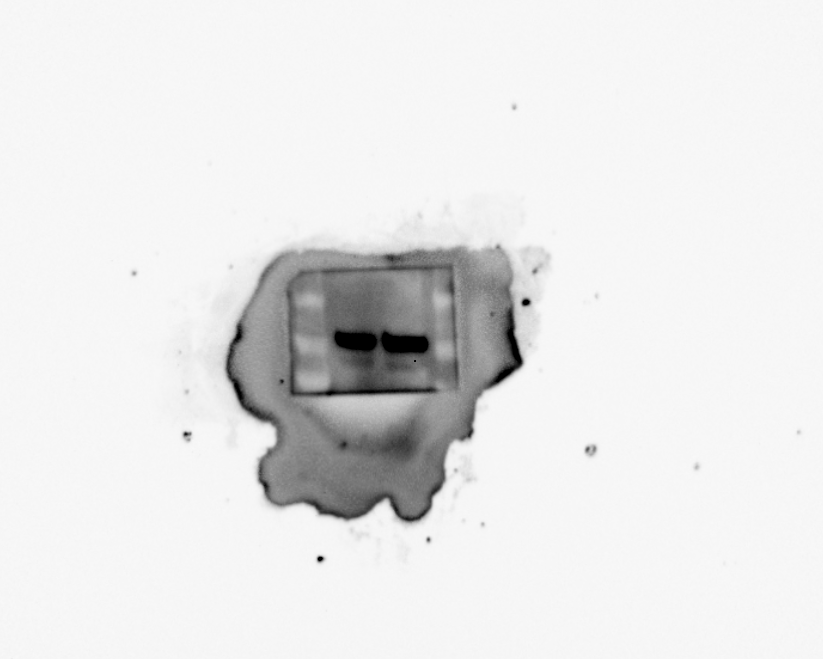


p-AKT


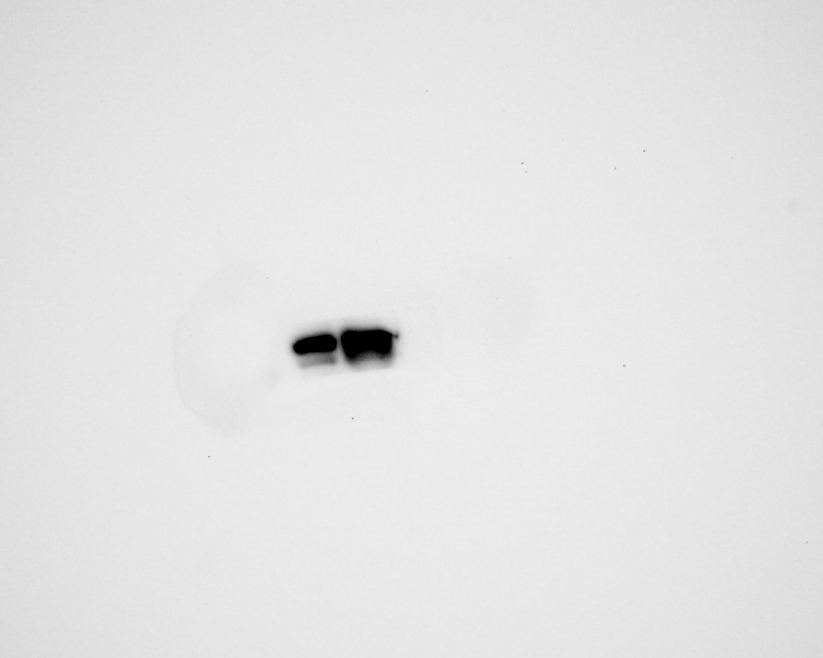


AKT


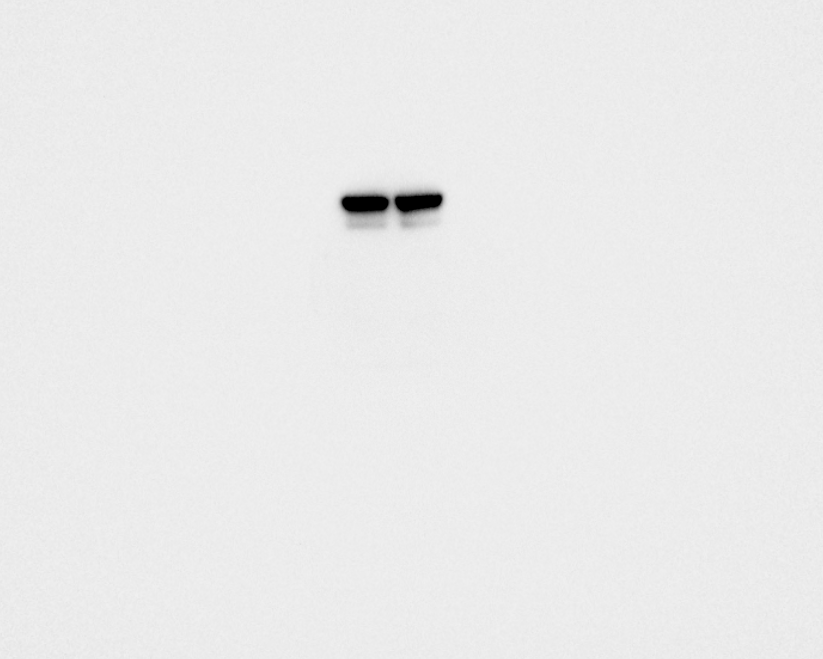


GAPDH


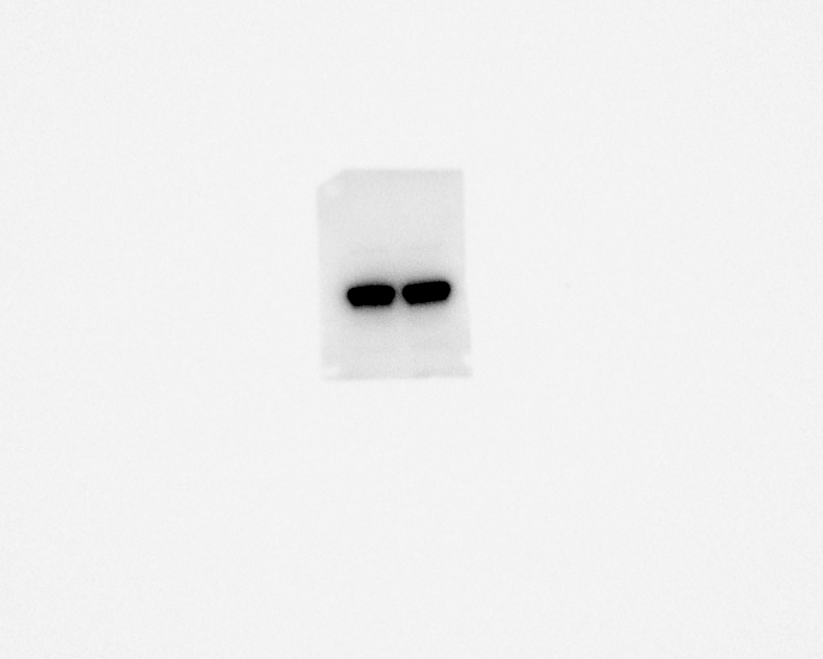


Fig6 C

Hep G2，Left

p-PDGFRa


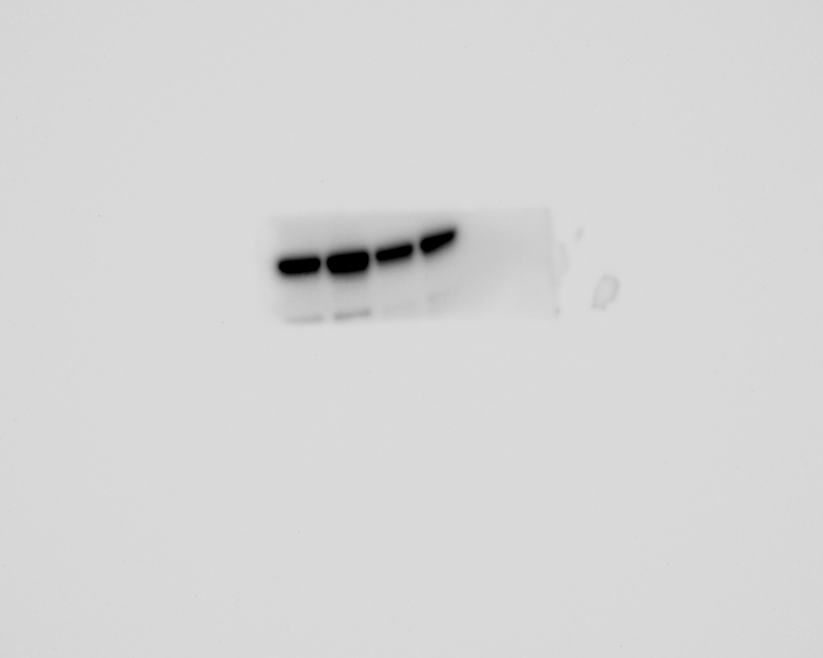


p-PI3K

PI3K

p-AKT

AKT

GAPDH

SK-Hep-1,

p-PDGFRa

p-PI3K

PI3K

p-AKT

AKT

GAPDH

Hep 3B, Righr

p-PDGFRa

p-PI3K

PI3K

p-AKT

AKT

GAPDH

Fig6 E

Hep G2

ANXA3

PDGF-AA

GAPDH

SK-Hep-1:

ANXA3

PDGF-AA

GAPDH

Hep3B:

ANXA3

PDGF-AA

GAPDH

Fig6 I

Hep G2

ANXA3

LC3B

GAPDH

SK-Hep-1:

ANXA3

LC3B

GAPDH

Hep3B

ANXA3

LC3B

GAPDH

Fig. S11

ANXA3

GAPDH:
